# QL1706 (anti-PD-1 IgG4/CTLA-4 antibody) plus chemotherapy with or without bevacizumab in advanced non-small cell lung cancer: a multi-cohort, phase II study

**DOI:** 10.1038/s41392-023-01731-x

**Published:** 2024-01-29

**Authors:** Yan Huang, Yunpeng Yang, Yuanyuan Zhao, Hongyun Zhao, Ningning Zhou, Yaxiong Zhang, Likun Chen, Ting Zhou, Gang Chen, Ting Wu, Lu Lu, Shilin Xue, Xiaoyan Kang, Li Zhang, Wenfeng Fang

**Affiliations:** 1 Department of Medical Oncology, Sun Yat-sen University Cancer Center, State Key Laboratory of Oncology in South China, Collaborative Innovation Center for Cancer Medicine, No. 651 Dongfeng East Road, Guangzhou, Guangdong 510060 China; 2 Department of Medicine, Qilu Pharmaceutical Co., Ltd., Jinan, China

**Keywords:** Lung cancer, Drug development

## Abstract

First-line chemoimmunotherapy (with or without bevacizumab) has improved outcomes in advanced non-small cell lung cancer (NSCLC). Here, this open-label, multi-cohort phase II study (NCT05329025) was done to investigate the safety and efficacy of QL1706 (a single bifunctional MabPair product against PD-1 and CTLA-4) and chemotherapy with or without bevacizumab in this population. Patients were enrolled into five different cohorts based on genotype (cohorts 1-4, epidermal growth factor receptor [EGFR] wild-type; cohort 5, EGFR-mutant and progressed on EGFR-tyrosine kinase inhibitors [TKIs]). Between June 11, 2021 and December 29, 2021, 91 patients were enrolled. Most frequent treatment-related adverse events (TRAEs) included decreased appetite (60 [65.9%]), anemia (60 [65.9%]), infusion-related reactions (48 [52.7%]), and pruritus (44 [48.4%]). Grade ≥ 3 TRAEs occurred in 30 (33.0%) patients. Twenty-seven (45%) patients with wild-type EGFR achieved partial response (PR) (objective response rate [ORR] = 45%) and had a median progression-free survival (mPFS) of 6.8 months (95% CI: 5.2-9.7). For 31 patients harboring mutated EGFR, 17 (54.8%) achieved PR (ORR = 54.8%), with an mPFS of 8.5 months (95% CI: 5.72-not evaluable). Overall, QL1706 plus chemotherapy, regardless of having bevacizumab, was generally tolerable and had promising antitumor activity for EGFR wild-type advanced NSCLC in first-line setting. Moreover, QL1706 plus chemotherapy and bevacizumab showed favorable antitumor activity for patients who had EGFR mutated NSCLC but failed in TKI therapy, demonstrating a potential for treating this population.

## Introduction

First-line PD-1/PD-L1 and CTLA-4 inhibitors have become a treatment backbone for driver-negative metastatic non-small-cell lung cancer (NSCLC). Several large-scale, phase III trials have revealed survival benefits of PD-1 inhibitors alone or plus CTLA-4 inhibitors over standard-of-care chemotherapy.^1,2^ Chemoimmunotherapy has also served as first-line options for various cancers (e.g., NSCLC). First-line pembrolizumab plus chemotherapy prolonged patients’ survival over placebo in KEYNOTE-189 and 407 trials.^3,4^ Furthermore, CheckMate-9LA trial also demonstrated overall survival (OS) advantages of dual immunotherapy plus chemotherapy versus chemotherapy (14.1 vs. 10.7 months).^5^ Based on the durable OS benefits at 2 years,^6^ nivolumab, ipilimumab and chemotherapy regimen gained the first approval as the dual immunotherapy-based regimen in advanced NSCLC.^7^ Despite the impressive survival benefits of this regimen, more than 50% of patients still had disease progression within 1 year and nearly 50% patients experienced severe adverse reactions.

EGFR tyrosine-kinase inhibitors (EGFR-TKIs) remain a standard therapy in EGFR-mutant advanced NSCLC.^8^ Despite the high initial objective response rate (ORR), tumors inevitably become resistant to EGFR-TKIs. The secondary EGFR mutations are considered one possible resistance mechanism. For instance, EGFR T790M mutation, which frequently occurs in 50%-60% of NSCLC patients who failed to EGFR-TKIs, impairs binding between first-/second-generation EGFR-TKIs and mutated EGFR.^9^ Osimertinib is a third-generation EGFR-TKI for treating NSCLC with EGFR T790M mutation. Currently, chemotherapy remains considered standard-of-care for patients progressing on osimertinib or those without EGFR T790M mutation but failed from first-/second-generation EGFR-TKIs; however, its survival benefit is still limited. Recently, there are numerous attempts to explore combinations of PD-1 inhibitors and chemotherapy in this population. PD-1 inhibitor sintilimab, anti-VEGF IBI305, and chemotherapy had positive results in the ORIENT-31 trial (hazard ratio [HR] = 0.46 compared to chemotherapy).^10^ Nevertheless, the KEYNOTE-789 and CheckMate-722 trials failed to show survival benefits with nivolumab and chemotherapy (HR = 0.75; *p* = 0.05).^11,12^

The IMpower 150 study that explored atezolizumab plus chemotherapy and bevacizumab regimen showed the prolonged survival in non-squamous NSCLC patients, irrespective of EGFR mutation status.^13,14^ Evidence from this study supported further investigations of chemoimmunotherapy plus bevacizumab in treatment-naïve NSCLC that progressed on prior EGFR-TKIs, regardless of EGFR mutation status.

We designed this trial according to the currently available evidences, aiming to investigate whether dual immunotherapy plus chemotherapy (2 or 4 cycles) with bevacizumab followed by maintenance therapy would provide durable response and survival benefit. QL1706 is a single bifunctional MabPair product, which have shown activity in advanced NSCLC from a phase I study on solid tumors.^15^ Therefore, this multi-cohort phase II study evaluated QL1706 combined with chemotherapy (with or without bevacizumab) in both EGFR wild-type and mutant advanced NSCLC.

## Results

### Patients

**Fig. 1:**
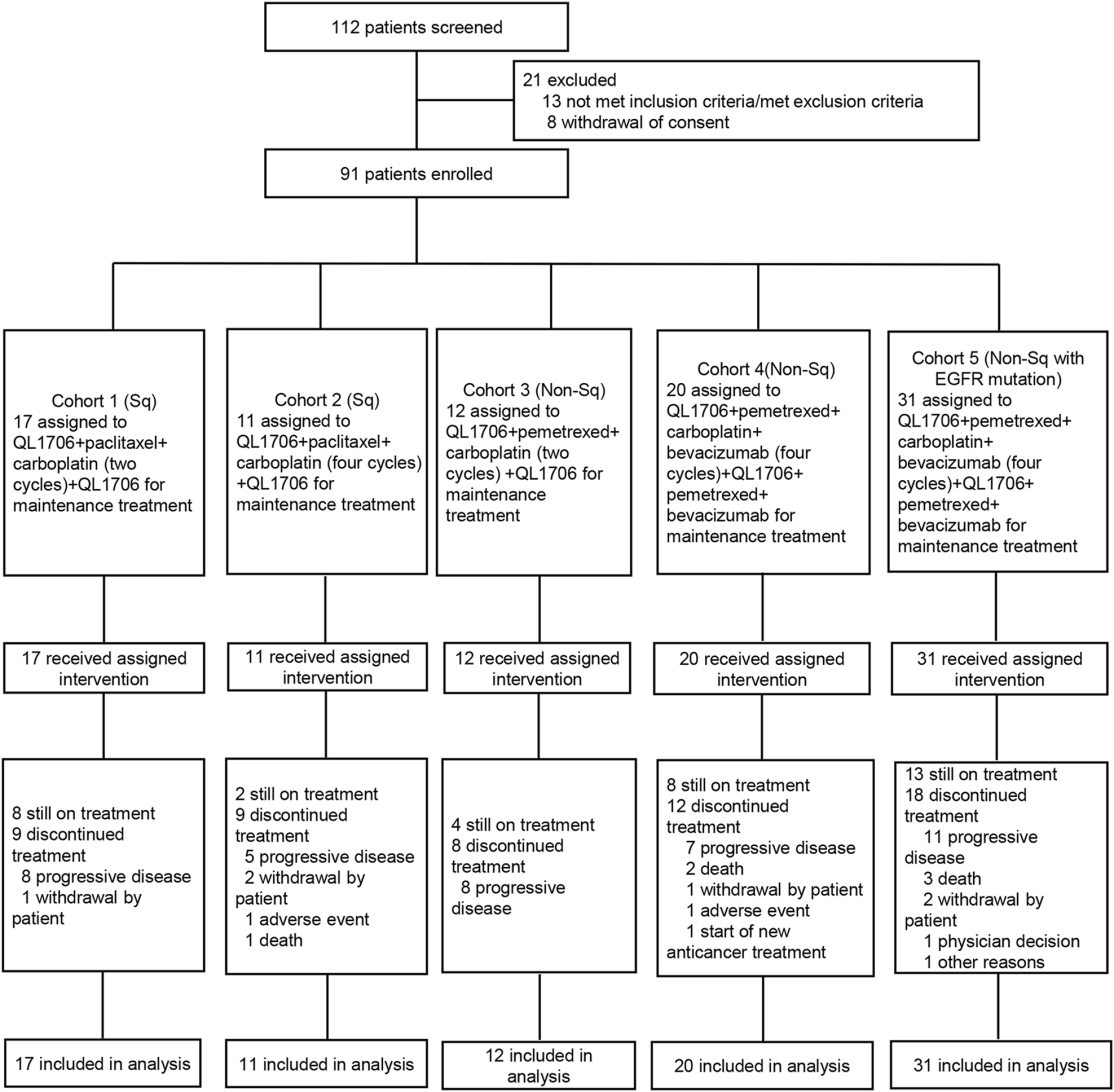
Patient disposition. Abbreviations: Sq squamous

From June 11, 2021 to December 29, 2021, among 112 screened patients, 91 were enrolled (Fig. 1). Table 1 summarized the baseline characteristics. Totally 60 patients had NSCLC harboring wild-type EGFR, of whom 28 (46.7%) had squamous and 32 (53.3%) had non-squamous histologic types. Most patients at stage IV (91.7%) and had Eastern Cooperative Oncology Group (ECOG) performance status of 1 (98.3%). Median follow-up of these patients was 12.6 months (range: 0.4-15.2) as of data cutoff (September 30, 2022). Total 22 (36.7%) patients were ongoing treatment. For each cohort, treatment discontinued mainly due to progressed disease (PD).

**Table 1:** Demographic and baseline characteristics

	Cohort 1 (*n* = 17)	Cohort 2 (*n* = 11)	Cohort 3 (*n* = 12)	Cohort 4 (*n* = 20)	Cohort 5 (*n* = 31)
Age, median (range), years	58 (28 to 72)	61 (49 to 70)	61 (37 to 74)	61.5 (50 to 74)	55 (39 to 74)
*Sex,* *n* (%)					
Male	14 (82.4)	10 (90.9)	8 (66.7)	17 (85.0)	17 (54.8)
Female	3 (17.6)	1 (9.1)	4 (33.3)	3 (15.0)	14 (45.2)
*Ethnicity,* *n* (%)					
Han Chinese	16 (94.1)	11 (100)	12 (100)	19 (95.0)	31 (100.0)
Other	1 (5.9)	0	0	1 (5.0)	0
*ECOG PS,* *n* (%)					
0	0	0	1 (8.3)	0	0
1	17 (100)	11 (100)	11 (91.7)	20 (100)	31 (100)
*Smoking status,* *n* (%)					
Never	3 (17.6)	2 (18.2)	4 (33.3)	6 (30.0)	15 (48.4)
Current or former	14 (82.4)	9 (81.8)	8 (66.7)	14 (70.0)	16 (51.6)
*Histologic subtype,* *n* (%)					
Squamous	16 (94.1)	11 (100)	NA	NA	NA
Non-squamous	NA	NA	12 (100)	20 (100)	31 (100)
Other	1 (5.9)	0	0	0	0
*Metastasis,* *n* (%)					
Bone	2 (11.8)	1 (9.1)	4 (33.3)	8 (40.0)	16 (51.6)
Brain	2 (11.8)	2 (18.2)	2 (16.7)	4 (20.0)	11 (35.5)
Liver	3 (17.6)	0	0	4 (20.0)	6 (19.4)
*Previous therapy*					
Antiangiogenics, *n* (%)	NA	NA	NA	NA	16 (51.6)
3rd generation EGFR TKIs, *n* (%)	NA	NA	NA	NA	19 (61.3)
*Disease stage at study entry*^*a*^*,* *n* (%)					
IIIB/C	2 (11.8)	1 (9.1)	1 (8.3)	1 (5.0)	0
IVA	8 (47.1)	6 (54.5)	6 (50.0)	8 (40.0)	9 (29.0)
IVB	7 (41.2)	4 (36.4)	5 (41.7)	11 (55.0)	22 (71.0)
*EGFR mutation types,* *n* (%)					
19 Del and T790M+	NA	NA	NA	NA	6 (19.4)
19 Del and T790M−	NA	NA	NA	NA	7 (22.6)
L858R and T790M+	NA	NA	NA	NA	5 (16.1)
L858R and T790M−	NA	NA	NA	NA	7 (22.6)
19 Del, L858R and T790M+	NA	NA	NA	NA	1 (3.2)
19 Del, L858R and T790M−	NA	NA	NA	NA	2 (6.5)
Others	NA	NA	NA	NA	3 (9.7)

Abbreviations: *ECOG* Eastern Cooperative Oncology Group, *EGFR* epidermal growth factor receptor, *PS* performance status, *TKI* tyrosine kinase inhibitor

^a^Staging was based on the American Joint Committee on Cancer (AJCC) eighth edition

In total 31 patients had NSCLC harboring mutated EGFR, of whom all patients were non-squamous histologic type, at stage IV and ECOG performance status of 1. Sixteen (51.6%) patients previously received antiangiogenics and 19 (61.3%) received prior third-generation EGFR-TKIs. This cohort had a median follow-up of 9.2 months (range: 1.1-12.0). Thirteen (41.9%) patients were still on treatment. Eleven (35.5%) patients discontinued study treatment because of PD.

### Efficacy

**Fig. 2:**
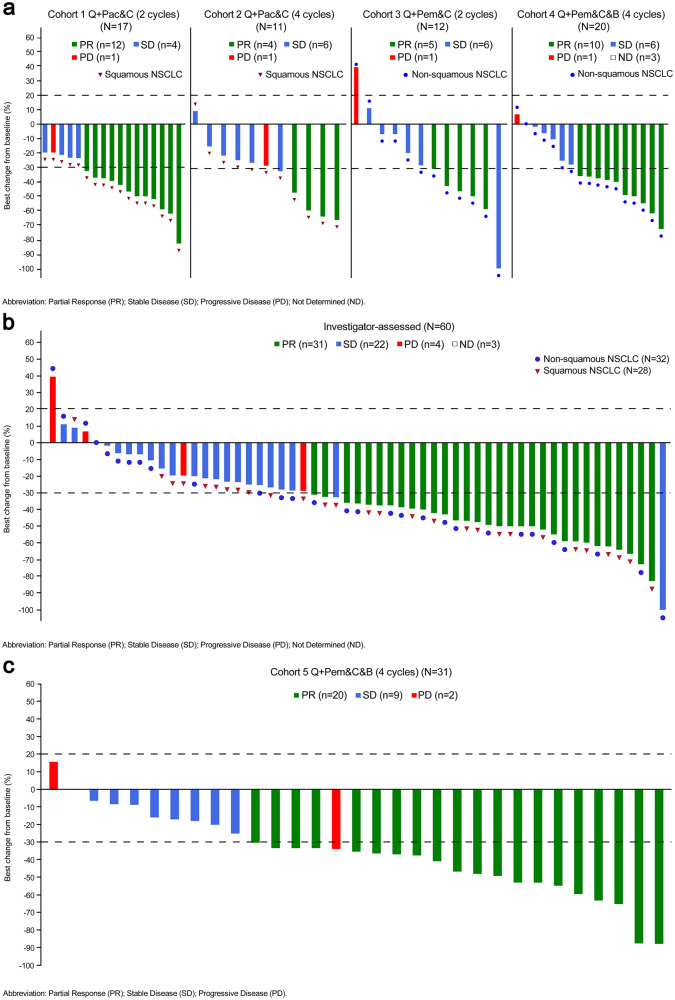
Antitumor activity in patients with EGFR wild-type and EGFR mutated tumors assessed per RECIST v1.1 by investigator. **a** Best percentage change from baseline in target lesion size in cohorts 1 to 4 is presented by cohort. **b** Best percentage change from baseline in target lesion size in cohorts 1 to 4 by histology. **c** Best percentage change from baseline in target lesion size in cohort 5. Abbreviations: Q QL1706, Pac paclitaxel, Pem pemetrexed, C carboplatin, B bevacizumab

**Fig. 3:**
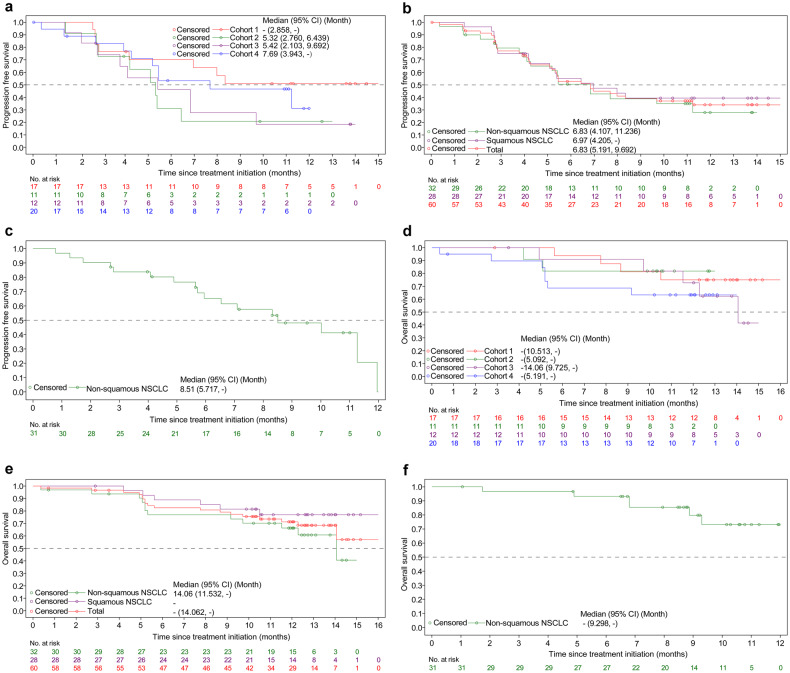
Progression-free survival in patients with EGFR wild-type and EGFR mutated tumors assessed per RECIST v1.1 by investigator and overall survival. **a** Progression-free survival in cohorts 1 to 4 by cohort. **b** Progression-free survival in cohorts 1 to 4 by histology and overall. **c** Progression-free survival in cohort 5. **d** Overall survival in cohorts 1 to 4 by cohort. **e** Overall survival in cohorts 1 to 4 by histology and overall. **f** Overall survival in cohort 5

In cohorts 1 to 4, 27 (45%) patients had a partial response (PR), resulting in an ORR of 45% (95% CI: 32.1-58.4) per Response Evaluation Criteria in Solid Tumors (RECIST) version 1.1 (Table 2). Reduction of target lesion size was observed in 56 (93.3%) patients (Fig. 2). Responders had a median time to response (mTTR) of 1.4 months (range: 1.2-4.4). At cutoff date, median duration of response (mDOR) was not reached (NR). Disease control rate (DCR) was 88.3% (95% CI: 77.4-95.2), including 26 (43.3%) patients with stable disease (SD). Median progression-free survival (mPFS) estimated from Kaplan-Meier analysis was 6.8 months (95% CI: 5.2-9.7), with estimated PFS rates of 39.1% (95% CI: 26.1-51.9) at 9 months and 34.1% (95% CI: 21.3-47.3) at 12 months (Fig. 3). Totally 18 (30.0%) patients had died at the time of analysis and median OS (mOS) was NR.

**Table 2:** Objective response and duration of response in total population

	Squamous		Non-squamous		Cohorts 1-4 population (*n* = 60)	Cohort 5 population (*n* = 31)
	Cohort 1 (*n* = 17)	Cohort 2 (*n* = 11)	Cohort 3 (*n* = 12)	Cohort 4 (n = 20)		
*Best overall response, n (%)*						
CR	0	0	0	0	0	0
PR	10 (58.8)	4 (36.4)	5 (41.7)	8 (40.0)	27 (45.0)	17 (54.8)
SD	6 (35.3)	6 (54.5)	6 (50.0)	8 (40.0)	26 (43.3)	12 (38.7)
PD	1 (5.9)	1 (9.1)	1 (8.3)	1 (9.1)	4 (6.7)	2 (6.5)
Not evaluable	0	0	0	0	0	0
Not able to be assessed^a^	0	0	0	3 (15.0)	3 (5.0)	0
ORR, *n* (%)	10 (58.8)	4 (36.4)	5 (41.7)	8 (40.0)	27 (45.0)	17 (54.8)
95% CI	(32.9, 81.6)	(10.9, 69.2)	(15.2, 72.3)	(19.1, 63.9)	(32.1, 58.4)	(36.0, 72.7)
DCR, *n* (%)	16 (94.1)	10 (90.9)	11 (91.7)	16 (80.0)	53 (88.3)	29 (93.5)
95% CI	(71.3, 99.9)	(58.7, 99.8)	(61.5, 99.8)	(56.3, 94.3)	(77.4, 95.2)	(78. 6, 99.2)
Median DOR, months (95% CI)	NR	NR	NR	9.9 (2.6-NE)	NR	7.0 (4.2-NE)
*Patients with a response who had ongoing responses*						
Rate (95% CI) at 3 months	83.3% (48.2, 95.6)	100% (100, 100)	75.0% (12.8, 96.1)	87.5% (38.7, 98.1)	85.7% (66.2, 94.4)	84.7% (59.7, 94.8)
Rate (95% CI) at 6 months	64.8% (31.0, 85.2)	50.0% (5.8, 84.5)	50.0% (5.8, 84.5)	75.0% (31.5, 93.1)	63.3% (42.5, 78.3)	53.5% (27.4, 74.0)
Rate (95% CI) at 9 months	55.6% (23.7, 78.7)	NR	50.0% (5.8, 84.5)	62.5% (22.9, 86.1)	55.9% (35.5, 72.1)	44.6% (19.1, 67.4)

Abbreviations: *CI* confidence interval, *CR* complete response, *DCR* disease control rate, *NR* not reached, *ORR* objective response rate, *PD* progressive disease, *PR* partial response, *SD* stable disease, *DOR* duration of response

^a^Three patients discontinued study treatment before the first scheduled post-baseline tumor assessment

The iRECIST efficacy results for cohorts 1 to 4 are presented in Supplementary Fig. 1. Thirty (50%) patients achieved iPR. The iORR was 50% (95% CI: 36.8-63.2). Responders had a median iDOR of 11.5 months (95% CI: 5.5-not evaluable [NE]). All these patients had a median iPFS of 8.3 months (95% CI: 5.4-12.9) (Supplementary Fig. 2). Three patients had iPR after iUPD and are still on treatment when data was locked for this analysis.

Among patients with squamous NSCLC, the ORR in the cohort 2 (QL1706 + 4-cycle chemotherapy) was numerically lower versus cohort 1 (QL1706 + 2-cycle chemotherapy) (36.4% versus 58.8%) (Table 2). The DCR in both cohorts was similar. The mPFS in the cohort 1 was NR and in the cohort 2 was 5.3 months (95% CI: 2.8-6.4). Similar ORR and DCR were observed between patients with non-squamous NSCLC in the cohort 3 (QL1706 + 2-cycle chemotherapy) and cohort 4 (QL1706 + 4-cycle chemotherapy+bevacizumab). Patients in the cohort 3 had a mPFS of 5.4 months (95% CI: 2.1- 9.7) and of 7.7 months (95% CI: 3.9-NE) in the cohort 4. Compared to cohort 3 (6-month, 46.3%; 9-month, 27.8%), apparently higher PFS rates at 6-month and 9-month were observed in cohort 4 (6-month, 53.3%; 9-month, 46.7%).

For patients in cohort 5 (QL1706 + 4 cycles of chemotherapy+bevacizumab) (EGFR-TKI resistant population), PR was observed in 17 (54.8%) patients, achieving an ORR of 54.8% (95% CI: 36.0-72.7) (Table 2). Reduction of target lesion size was observed in 29 (93.5%) patients (Fig. 2). Responders had a mTTR of 1.5 months (range: 1.3-6.9) and mDOR of 7.0 months (95% CI: 4.2-NE). Twelve (38.7%) patients achieved SD. The DCR was 93.5% (95% CI: 78.6-99.2). The mPFS was 8.5 months (95% CI: 5.7, -) (Fig. 3). Kaplan-Meier estimates for 9-month PFS rate was 48.1% (95% CI: 27.9-65.8). With 6 (19.4%) deaths occurred, mOS was NR at the time of this analysis.

The efficacy results per iRECIST in cohort 5 are presented in Supplementary Fig. 1. In cohort 5, 17 (54.8%) patients achieved iPR, with an iORR of 54.8% (95% CI: 36.0-72.7) and median iDOR of 7.0 months (95% CI: 4.2-NE). Median iPFS as per iRECIST was 8.5 months (95% CI: 6.0-NE) (Supplementary Fig. 2).

In cohort 5, 10 (52.6%) of 19 patients who received prior third-generation EGFR-TKIs achieved PR, with an ORR of 52.6% (95% CI: 28.9-75.6). Eight (42.1%) patients achieved SD. The DCR was 94.7% (95% CI: 74.0-99.9). A total of 16 patients previously received antiangiogenics (6 received bevacizumab and 12 received anlotinib). Nine (56.3%) patients achieved PR. The ORR was 56.3% (95% CI: 29.9-80.2). Seven (43.8%) patients achieved SD. The DCR was 100% (95% CI: 79.4-100.0).

### Safety

In cohorts 1 to 4, treatment-related adverse events (TRAEs) occurred in 58 (96.7%) patients (Table 3), with 19 (31.7%) being grade ≥ 3. No grade 4 or higher TRAEs occurred. All patients with squamous NSCLC in cohort 1 (QL1706 + 2 cycles of chemotherapy) and cohort 2 (QL1706 + 4 cycles of chemotherapy) experienced TRAEs. The following TRAEs had a higher incidence in cohort 2 (versus cohort 1) (difference>20%): hypoesthesia (90.9% vs 41.2%); neutrophil count decreased (54.5% vs 11.8%); white blood cell count decreased (45.5% vs 17.6%); and flushing (27.3% vs 5.9%). Treatment-related infectious pneumonia occurred in 1 patient each in cohort 1 (5.9%) and cohort 2 (9.1%). For patients who had non-squamous NSCLC, entire cohort 3 (QL1706 + 2 cycles of chemotherapy) and 90% patients in cohort 4 (QL1706 + 4 cycles of chemotherapy+bevacizumab) experienced TRAEs. The following TRAEs had a higher incidence in the cohort 4 (versus cohort 3) (difference>20%): amylase increased (25% vs 0); neutrophil count decreased (30% vs 8.3%); and white blood cell count decreased (30% vs 8.3%).

**Table 3:** Summary of Safety Results

	Squamous		Non-squamous			
	Cohort 1 (*n* = 17)	Cohort 2 (*n* = 11)	Cohort 3 (*n* = 12)		Cohort 4 (*n* = 20)	Cohort 5 (*n* = 31)
Treatment-related AEs, *n* (%)	17 (100)	11 (100)	12 (100)	18 (90.0)		30 (96.8)
Grade ≥ 3 treatment-related AEs, *n* (%)	3 (17.6)	4 (36.4)	2 (16.7)	10 (50.0)		11 (35.5)
Treated-related SAEs, *n* (%)	4 (23.5)	2 (18.2)	2 (16.7)	7 (35.0)		11 (35.5)
Treatment-related AEs leading to dose interruption	11 (64.7)	7 (63.6)	5 (41.7)	15 (75.0)		18 (58.1)
Treatment-related AEs leading to discontinuation of study treatment	0	1 (9.1)	0	4 (20.0)		1 (3.2)
Treatment-related AEs leading to death	0	0	0	0		2 (6.5)
*Treatment-related AEs* *≥* *20% in any group, n (%)*						
Decreased appetite	11 (64.7)	7 (63.6)	7 (58.3)	14 (70.0)		21 (67.7)
Anemia	8 (47.1)	7 (63.6)	7 (58.3)	14 (70.0)		24 (77.4)
Infusion-related reactions	11 (64.7)	7 (63.6)	7 (58.3)	11 (55.0)		12 (38.7)
Pruritus	12 (70.6)	8 (72.7)	8 (66.7)	7 (35.0)		9 (29.0)
Rash	11 (64.7)	6 (54.5)	5 (41.7)	4 (20.0)		8 (25.8)
Fatigue	6 (35.3)	6 (54.5)	4 (33.3)	9 (45.0)		6 (19.4)
Hypoesthesia	7 (41.2)	10 (90.9)	4 (33.3)	3 (15.0)		3 (9.7)
Constipation	8 (47.1)	2 (18.2)	6 (50.0)	7 (35.0)		14 (45.2)
Nausea	6 (35.3)	1 (9.1)	6 (50.0)	4 (20.0)		11 (35.5)
Arthralgia	7 (41.2)	5 (45.5)	2 (16.7)	2 (10.0)		4 (12.9)
Amylase increased	6 (35.3)	5 (45.5)	0	5 (25.0)		10 (32.3)
Neutrophil count decreased	2 (11.8)	6 (54.5)	1 (8.3)	6 (30.0)		9 (29.0)
White blood cell count decreased	3 (17.6)	5 (45.5)	1 (8.3)	6 (30.0)		9 (29.0)
AST increased	5 (29.4)	2 (18.2)	2 (16.7)	4 (20.0)		17 (54.8)
ALT increased	3 (17.6)	3 (27.3)	4 (33.3)	3 (15.0)		13 (41.9)
Hypothyroidism	5 (29.4)	2 (18.2)	1 (8.3)	4 (20.0)		11 (35.5)
Pyrexia	3 (17.6)	1 (9.1)	2 (16.7)	5 (25.0)		4 (12.9)
Diarrhea	4 (23.5)	2 (18.2)	2 (16.7)	3 (15.0)		6 (19.4)
Stomatitis	2 (11.8)	2 (18.2)	3 (25.0)	4 (20.0)		4 (12.9)
Pain in extremity	5 (29.4)	2 (18.2)	1 (8.3)	2 (10.0)		2 (6.5)
Hyperthyroidism	2 (11.8)	3 (27.3)	1 (8.3)	2 (10.0)		5 (16.1)
Hypoproteinemia	4 (23.5)	1 (9.1)	1 (8.3)	2 (10.0)		0
Vomiting	3 (17.6)	2 (18.2)	3 (25.0)	0		9 (29.0)
Platelet count decreased	2 (11.8)	1 (9.1)	1 (8.3)	3 (15.0)		10 (32.3)
Proteinuria	0	0	1 (8.3)	5 (25.0)		11 (35.5)
Flushing	1 (5.9)	3 (27.3)	2 (16.7)	0		2 (6.5)
Peripheral edema	1 (5.9)	1 (9.1)	3 (25.0)	1 (5.0)		4 (12.9)
Epistaxis	1 (5.9)	0	0	4 (20.0)		6 (19.4)
Immune-related AEs, *n* (%)	14 (82.4)	7 (63.6)	8 (66.7)	13 (65.0)		18 (58.1)
*Immune-related AEs* *≥* *10% in any group, n (%)*						
Pruritus	7 (41.2)	6 (54.5)	4 (33.3)	6 (30.0)		5 (16.1)
Rash	5 (29.4)	5 (45.5)	3 (25.0)	4 (20.0)		4 (12.9)
Infusion-related reactions	9 (52.9)	5 (45.5)	4 (33.3)	2 (10.0)		3 (9.7)
Amylase increased	4 (23.5)	5 (45.5)	0	3 (15.0)		3 (9.7)
Hypothyroidism	5 (29.4)	2 (18.2)	0	4 (20.0)		9 (29.0)
ALT increased	3 (17.6)	2 (18.2)	3 (25.0)	2 (10.0)		7 (22.6)
AST increased	4 (23.5)	1 (9.1)	2 (16.7)	1 (5.0)		8 (25.8)
Lipase increased	3 (17.6)	2 (18.2)	1 (8.3)	1 (5.0)		3 (9.7)
Hyperthyroidism	2 (11.8)	3 (27.3)	0	2 (10.0)		5 (16.1)
Immune-mediated lung disease	0	0	1 (8.3)	2 (10.0)		2 (6.5)
*Grade* *≥* *3 immune-related AEs, n (%)*						
Lipase increased	1 (5.9)	1 (9.1)	0	1 (5.0)		0
Rash	0	1 (9.1)	1 (8.3)	0		0
Immune-mediated myocarditis	0	0	0	0		1 (3.2)
Hyperlipidemia	0	1 (9.1)	0	0		0

Abbreviations: *AE* adverse event, *ALT* alanine aminotransferase, *AST* aspartate transaminase, *SAE* serious adverse event

In cohorts 1 to 4, 42 (70%) patients experienced immune-related adverse events (irAEs) (Table 3). For patients with squamous histologic type, 14 (82.4%) in the cohort 1 experienced irAEs and 7 (63.6%) in the cohort 2. The following irAEs had a higher incidence in the cohort 2 compared with cohort 1 (difference > 20%): amylase increased (45.5% vs 23.5%). For patients with non-squamous NSCLC, 8 (66.7%) patients in the cohort 3 and 13 (65.0%) patients in the cohort 4 experienced irAEs.

In cohort 5 (QL1706 + 4 cycles of chemotherapy+bevacizumab) (EGFR-TKI resistant population), 30 (96.8%) patients experienced TRAEs. Most frequent TRAEs (>40%) included anemia (77.4%), decreased appetite (67.7%), aspartate aminotransferase (AST) increased (54.8%), constipation (45.2%), and alanine aminotransferase (ALT) increased (41.9%). Four patients (12.9%) had treatment-related infectious pneumonia. Eleven (35.5%) patients reported grade ≥ 3 TRAEs and 2 (6.5%) reported grade 4 TRAEs: small intestinal perforation and neutrophil count decreased. Both patients received treatment for the adverse events and recovered. Besides, grade 5 pneumonia was observed in 2 patients (6.5%).

In cohort 5, 18 (58.1%) patients experienced irAEs. The most common irAEs (>20%) were hypothyroidism (29%); AST increased (25.8%); and ALT increased (22.6%). Immune-mediated myocarditis occurred in 1 (3.2%) patient.

During this study, 41 (45.1%) patients were not treated with bevacizumab and 50 (54.9%) patients were treated with bevacizumab (Supplementary Table 1). The following AEs were more common in patients with bevacizumab treatment compared to those without (difference > 10%): anemia (76% vs 53.7%); AST increased (42% vs 22%); platelet count decreased (26% vs 9.8%); hypothyroidism (30% vs 19.5%); epistaxis (20% vs 2.4%); and proteinuria (32% vs 2.4%). Grade ≥ 3 TRAEs occurred more frequently in bevacizumab-treated patients, with anemia being most common (20%, versus 4.9% in patients not treated with bevacizumab).

## Discussion

This phase II study demonstrated encouraging activity of first-line QL1706 plus chemotherapy with or without bevacizumab for advanced NSCLC, regardless of patient’s histologic type. This regimen reported an encouraging and durable response, with an ORR of 45% (95% CI: 32.1-58.4) and mDOR of NR. Our ORR and mPFS results in EGFR wild-type patients was comparable to results from dual immunotherapy (nivolumab and ipilimumab) and chemotherapy in the CheckMate-9LA study.^5^ Moreover, in population with non-squamous NSCLC, mPFS slightly favored the regimen of 4 cycles QL1706 plus chemotherapy with bevacizumab, supporting its further investigations in phase III studies.

Results from the EGFR-mutant subgroup progressing on EGFR-TKIs suggested the clinical benefit of QL1706 plus chemotherapy with bevacizumab, with a favorable response rate (ORR = 54.8%), even in those previously received antiangiogenics or third-generation EGFR-TKIs. Moreover, our mPFS (8.5 months) seems numerically longer than those reported with sintilimab, chemotherapy and bevacizumab in the ORIENT-31 trial (mPFS = 6.9 months; ORR = 45%),^10^ implying the potential advantages of our regimens over standard PD-1 inhibitor plus chemotherapy in this population. Here, mOS was not yet reached, with estimated rates of 93.2% and 79.8% at 6 and 9 months. Long-term follow up for OS is still ongoing.

Based on the KEYNOTE 189 study, the observed shorter mOS and mPFS in PD-L1-negative patients over ITT population highlighted the unmet medical need for this PD-L1-negative population.^16^ Subsequent large-scale randomized trials revealed that PD-1 and CTLA-4 dual blockade may have survival benefit for this population, as evidenced by the encouraging mOS improvement with nivolumab and ipilimumab over chemotherapy in PD-L1 < 1% subgroup (CheckMate-227, 17.4 vs. 12.2 months; HR, 0.65; CheckMate-9LA, 17.7 vs. 9.8 months; HR, 0.67).^6,17^ So far, several trials (KEYNOTE-789, CheckMate-722, and IMpower151) have investigated the chemoimmunotherapy with or without bevacizumab in patients with EGFR-mutant NSCLC after prior EGFR-TKIs, while none showed PFS improvement.^18,19^ Additionally, the ORIENT-31 study only showed PFS benefit of sintilimab plus bevacizumab and chemotherapy, but without prolonged OS.^20^ Here, mPFS (8.5 months) and ORR (54.8%) with QL1706 plus chemotherapy and bevacizumab seem favorable versus available evidences with similar regimens in EGFR-mutant tumors. Our regimen was manageable, with a similar safety profile to other regimens with chemotherapy, bevacizumab, and PD-1/CTLA-4 inhibitor. TRAEs leading to treatment discontinuation were infrequent (cohorts 1 to 4, 8.3%; cohort 5, 3.2%). Based on the encouraging efficacy and acceptable safety, dual immunotherapy plus chemotherapy and bevacizumab may be a safe and effective option for this population.

Despite the survival benefit of chemoimmunotherapy for advanced NSCLC, it usually causes severe toxicity, particularly in the dual immunotherapy regimen (e.g., POSEIDON trial of durvalumab and tremelimumab),^21^ limiting the clinical application. Modifying chemotherapy cycles is a common strategy to minimize toxicities of combination therapy. The CheckMate-9LA study preliminarily demonstrated the improved benefit-risk profile of short-course chemotherapy, nevertheless, few direct comparisons were made between dual immunotherapy plus limited-course or full-course of chemotherapy. Here, although frequent hematologic toxicities and infusion-related reactions were observed with 4 cycles of QL1706 plus chemotherapy with bevacizumab, TRAEs causing treatment discontinuation were generally comparable between cohort 1 (2-cycle chemotherapy; 0/17) and cohort 2 (4-cycle chemotherapy; 1/11[9.1%]). Besides, 20% patients in cohort 4 (QL1706 + 4-cycle chemotherapy+bevacizumab) discontinued treatment due to TRAEs while none in cohort 3 (QL1706 + 2-cycle of chemotherapy). Regarding efficacy, short-course regimens (2-cycle) did not affect tumor control, with similar ORRs to 4-cycle regimens (cohorts 1 vs. 2: 58.8% vs. 36.4%; cohorts 3 vs. 4: 41.7% vs. 40.0%). Nonetheless, the 4-cycle regimen was found to be associated with longer PFS than 2-cycle regimen (mPFS: 7.7 vs 5.4 months; 9-month PFS, 46.7% vs 27.8%). Because of limited sample-size in each cohort, investigations in larger population remain needed. Besides, our study was limited by the unavailability of tumor tissue samples, lacking subgroup efficacy analyses by PD-L1 status. Finally, owing to small sample size in each cohort, it was underpowered to make statistical comparisons across the groups.

In conclusion, QL1706 plus chemotherapy (with or without bevacizumab) showed manageable safety profile for advanced NSCLC and encouraging activity in first-line treatment of EGFR wild-type NSCLC. Moreover, QL1706 plus chemotherapy and bevacizumab is active for EGFR-mutated mutant patients progressing on prior EGFR-TKIs, showing potential to treat this patient population.

## Materials and methods

### Study design and patients

This is a multi-cohort, open-label, single-arm phase II trial conducted in China. Ethics committee of Sun Yat-sen University Cancer Center approved the study protocol. The study obtained written informed consent from all participators before enrolment and was conducted according to the Good Clinical Practice guidelines and Declaration of Helsinki.

Key inclusion criteria included aged ≥ 18 years; histologically/cytologically confirmed NSCLC at stage IIIB/C-IV per American Joint Committee on Cancer (AJCC) 8th edition and not suitable to receive radical surgery or other local radical therapy; systemic treatment-naïve except for (neo)adjuvant or radical chemoradiotherapy discontinued for over 6 months before study treatment; ECOG performance status of 0-1; and ≥1 target lesions per RECIST 1.1. Non-squamous NSCLC patients were required to confirm EGFR, ALK, and ROS1 wild-type status by histology or cytology tests; and those with EGFR-sensitizing mutation were eligible if they had progressed on prior EGFR-TKIs or cannot tolerate EGFR-TKIs, and those with EGFR T790M mutation were required to be prior third-generation EGFR-TKIs failure or intolerable. Main exclusion criteria were symptomatic brain metastases or active autoimmune disease in the previous 2 years; active infection in the previous 14 days requiring systemic antibiotics therapy; had disease requiring systemic corticosteroids therapy or other immunosuppressant in the previous 2 weeks; previously received immunotherapy (including immune checkpoint inhibitors or agonists, and immune cell therapy); received >30 Gy radiotherapy to thoracic cavity or lungs in the previous 6 months, or palliative radiotherapy to other sites or other local therapy in the previous 2 weeks and did not recover; high bleeding tendency or coagulation dysfunction, or were on anticoagulant or thrombolytic therapy.

### Procedures

Patients were recruited into 5 different cohorts (cohort 1-5) based on their histologic type and EGFR status. All drugs were given on day 1 in a 21-day cycle (every 3 weeks) intravenously at protocol-defined doses: QL1706 (5 mg/kg), paclitaxel (175 mg/m^2^), carboplatin (AUC 5/6), pemetrexed (500 mg/m^2^), and bevacizumab (15 mg/kg). Patients having squamous NSCLC received QL1706, paclitaxel, and carboplatin for 2 (cohort 1) or 4 cycles (cohort 2), and followed by QL1706 maintenance. Patients with non-squamous wild-type EGFR NSCLC received QL1706, pemetrexed, and carboplatin for 2 cycles, followed by QL1706 maintenance (cohort 3); or QL1706, pemetrexed, carboplatin, and bevacizumab for 4 cycles, then maintenance with QL1706, pemetrexed, and bevacizumab (cohort 4). Patients with EGFR-sensitizing mutations progressing on EGFR-TKIs received QL1706, pemetrexed, carboplatin, and bevacizumab for 4 cycles, then maintenance with QL1706, pemetrexed, and bevacizumab (cohort 5).

Dose adjustment was not permitted for QL1706 and bevacizumab but permitted for chemotherapy. Treatment continued until disease progression, no clinical benefit, completion of 2-year treatment, or other protocol-specified conditions, whichever occurred first.

Tumor imaging were performed by MRI (for brain imaging) or CT (for abdomen, pelvis and chest) at baseline, every 6 weeks at the first 48 weeks, and every 12 weeks thereafter. For patients with brain metastasis at baseline, radiographic scan to head was performed every 12 weeks. Confirmation of response was required at 4-8 weeks after initial response as per RECIST 1.1. Adverse events were monitored during treatment period until 90 days after last dose of study drugs, or starting new anti-tumor therapy and graded per National Cancer Institute Common Terminology Criteria for Adverse Events (NCI-CTCAE) version 5.0. Other details are listed in the protocol provided in supplemental material.

### Outcomes

The primary endpoint was the safety and tolerability assessed by AEs, TRAEs, laboratory results, and vital signs. Secondary endpoints were ORR per RECIST 1.1 (investigators-assessed confirmed complete response [CR] or PR); DOR (time from first evidence of CR/PR to first documented PD or death); PFS (time from treatment initiation to first documented PD or death due to any cause); and OS (time from the treatment initiation to any death).

### Statistical analysis

Because no specific hypothesis testing was needed, sample size of each cohort was determined as per an estimation design. Cohort 1-4 each targeted to enroll 15 patients. As more patients were expected to have been pretreated with EGFR-TKIs in Asia, the target enrolment for cohort 5 was set to 30 patients. Total 80 patients were planned for cohorts 5 and 3 since they were regarded as similar originally.

Efficacy population consists of patients who received ≥1 doses of study drugs. Response rates with corresponding 95% CIs were estimated by Clopper-Pearson exact method. Time-to-event endpoints were estimated with Kaplan-Meier method. Safety assessments were performed for patients who received ≥1 doses of study drugs and had ≥1 post-dose safety result. Baseline characteristics and safety were descriptively summarized. SAS 9.4 (SAS Institute, Cary, NC) was used for statistical analyses.

### Supplementary information

Supplementary material

Study protocol

## Data Availability

Dataset of this study could be obtained from corresponding author with a reasonable request.
