# Supplementary material for: QL1706 (anti-PD-1 IgG4/CTLA-4 antibody) plus chemotherapy with or without bevacizumab in advanced non-small cell lung cancer: a multi-cohort, phase II study
Source: Signal Transduct Target Ther. 2024 Jan 29;9:23. doi: 10.1038/s41392-023-01731-x (PMC10822847; doi:10.1038/s41392-023-01731-x)
Supplement: Supplementary file 1 — Supplementary material [file 41392_2023_1731_MOESM1_ESM.docx]

Supplementary Materials for

QL1706 (anti-PD-1 IgG4/CTLA-4 antibody) plus chemotherapy with or without bevacizumab in advanced non-small cell lung cancer: a multi-cohort, phase II study

Yan Huang, Yunpeng Yang, Yuanyuan Zhao, Hongyun Zhao, Ningning Zhou, Yaxiong Zhang, Likun Chen, Ting Zhou, Gang Chen, Ting Wu, Lu Lu, Shilin Xue, Xiaoyan Kang, Li Zhang*, Wenfeng Fang*

Correspondence to: fangwf@sysucc.org.cn, zhangli@sysucc.org.cn

**This PDF file includes:**

Figures S1 to S2

Tables S1

**Other Supplementary Materials for this manuscript include the following:**

Study protocol

**Figure S1**

**
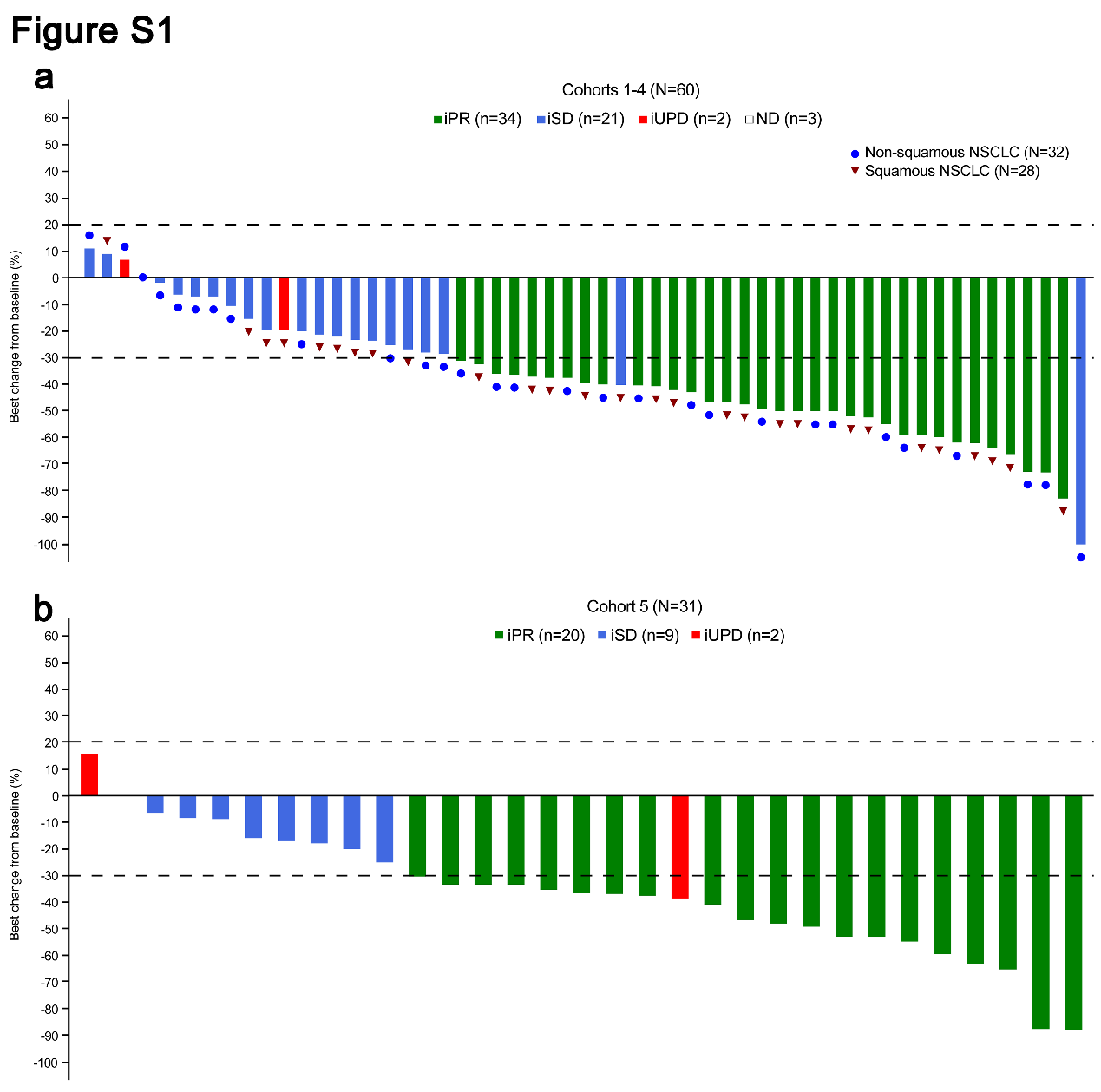
**

**Supplementary Figure S1.** Antitumor activity in patients with EGFR wild-type and EGFR mutated tumors assessed per iRECIST by investigator. **a** Best percentage change from baseline in target lesion size in cohorts 1 to 4. **b** Best percentage change from baseline in target lesion size in cohort 5. Abbreviations: iPR, immune partial response; iSD, immune stable disease; iUPD, immune unconfirmed progressive disease; ND, not determined; NSCLC, non-small cell lung cancer.

**Figure S2**


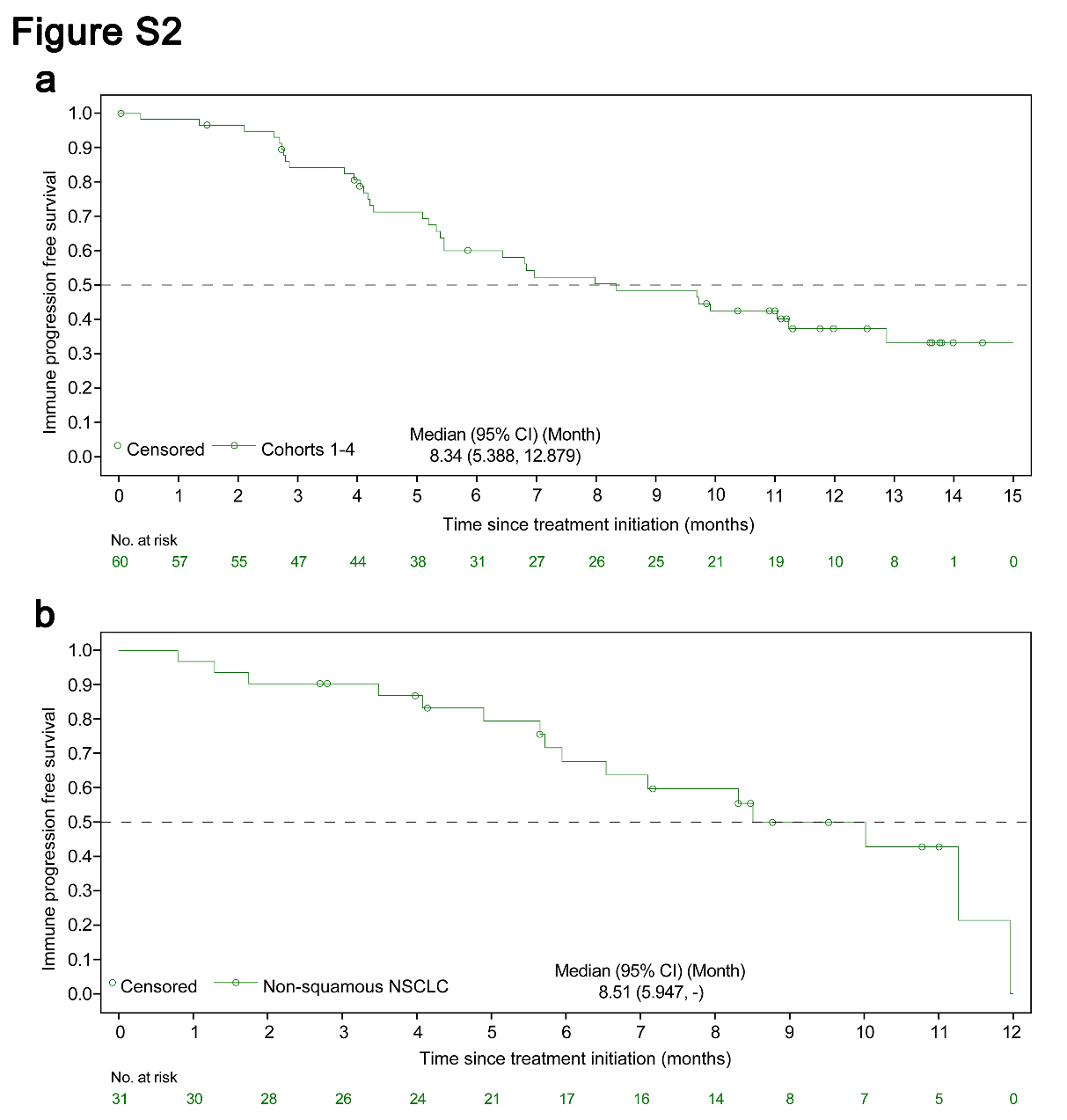


**Supplementary Figure S2.** Progression-free survival in patients with EGFR wild-type and EGFR mutated tumors assessed per iRECIST by investigator. **a** Progression-free survival in cohorts 1 to 4. **b** Progression-free survival in cohort 5. Abbreviations: NSCLC, non-small cell lung cancer.

**Table S1.**

Summary of Treatment-Related Adverse Events in Patients without or with Bevacizumab Use during the Study (Safety Population)

|  | **Without bevacizumab use (n=41)** | | **With bevacizumab use (n=50)** | |
| --- | --- | --- | --- | --- |
|  | **Total** | **Grade≥3** | **Total** | **Grade≥3** |
| Treatment-related adverse events≥20% in any group, n (%) |  |  |  |  |
| Decreased appetite | 25 (61.0) | 0 | 35 (70.0) | 0 |
| Anemia | 22 (53.7) | 2 (4.9) | 38 (76.0) | 10 (20.0) |
| Infusion-related reactions | 25 (61.0) | 0 | 23 (46.0) | 1 (2.0) |
| AST increased | 9 (22.0) | 0 | 21 (42.0) | 0 |
| ALT increased | 10 (24.4) | 0 | 16 (32.0) | 2 (4.0) |
| Amylase increased | 11 (26.8) | 0 | 15 (30.0) | 0 |
| WBC count decreased | 9 (22.0) | 2 (4.9) | 15 (30.0) | 2 (4.0) |
| Neutrophil count decreased | 9 (22.0) | 3 (7.3) | 15 (30.0) | 6 (12.0) |
| Platelet count decreased | 4 (9.8) | 0 | 13 (26.0) | 5 (10.0) |
| Lipase increased | 7 (17.1) | 2 (4.9) | 10 (20.0) | 1 (2.0) |
| Constipation | 16 (39.0) | 0 | 21 (42.0) | 0 |
| Nausea | 13 (31.7) | 0 | 15 (30.0) | 0 |
| Fatigue | 16 (39.0) | 0 | 15 (30.0) | 0 |
| Pruritus | 28 (68.3) | 0 | 16 (32.0) | 0 |
| Rash | 22 (53.7) | 2 (4.9) | 12 (24.0) | 0 |
| Arthralgia | 14 (34.1) | 0 | 6 (12.0) | 0 |
| Hypoesthesia | 21 (51.2) | 0 | 6 (12.0) | 0 |
| Hypothyroidism | 8 (19.5) | 0 | 15 (30.0) | 0 |
| Epistaxis | 1 (2.4) | 0 | 10 (20.0) | 0 |
| Proteinuria | 1 (2.4) | 0 | 16 (32.0) | 1 (2.0) |

Abbreviations: ALT, alanine aminotransferase; AST, aspartate transaminase; WBC, white blood cell
