# Supplementary material for: QL1706 (anti-PD-1 IgG4/CTLA-4 antibody) plus chemotherapy with or without bevacizumab in advanced non-small cell lung cancer: a multi-cohort, phase II study
Source: Signal Transduct Target Ther. 2024 Jan 29;9:23. doi: 10.1038/s41392-023-01731-x (PMC10822847; doi:10.1038/s41392-023-01731-x)
Supplement: Supplementary file 2 — Study protocol [file 41392_2023_1731_MOESM2_ESM.docx]

NMPA Clinical Trial Receiving No.: CXSL1900118 (State)
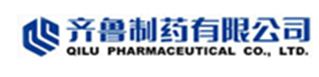


CLINICAL STUDY OF THE SAFETY AND EFFICACY OF QL1706 IN COMBINATION WITH CHEMOTHERAPY IN FIRST-LINE TREATMENT OF PATIENTS WITH STAGE IIIB/C AND STAGE IV NON-SMALL CELL LUNG CANCER

Protocol No.: QL1706-201

Study Phase: II

Drug No.: QL1706

Drug Name: QL1706 Injection

Leading Site: Sun Yat-Sen University Cancer Center

Principal Investigator: Li Zhang

Version No.: 1.2

Version Date: Jul. 8, 2021

**Sponsor:** Qilu Pharmaceutical Co., Ltd.

| Confidentiality Statement  The information contained in this protocol is confidential and is for the use of clinical investigators only. The disclosures are not permitted unless requested by current laws or regulations. The copyright is owned by Qilu Pharmaceutical Co., Ltd. Any copies or distributions of the information herein to any individuals are not allowed unless a confidentiality agreement has been signed with Qilu Pharmaceutical Co., Ltd. |
| --- |

Version History/Amendment History

| **Document** | **Version date** | **Reasons for amendment and summary of changes** |
| --- | --- | --- |
| 1.0 | Dec. 8, 2020 | Not applicable |
| 1.1 | Jan. 28, 2021 | Revisions based on comments from the Ethics Committee:  (1) For subjects who showed PD per RECIST v1.1 but continued to receive QL1706, if confirmed PD (iCPD as per iRECIST) was observed, QL1706 should be discontinued. Revised corresponding content.  (2) QL1706 should be permanently discontinued when grade 2 immune-related myocarditis was observed. |
| 1.2 | Jul. 8, 2021 | To evaluate the safety and efficacy of QL1706 combined with chemotherapy ± bevacizumab in the treatment of driver gene-positive non-squamous non-small cell lung cancer (NSCLC):  (1) Added new population to Cohort 2: EGFR-positive advanced NSCLC patients who failed EGFR-TKI therapy, with a total of 40 subjects enrolled to Cohort 2; treatment: QL1706 + pemetrexed + carboplatin ± bevacizumab. |

**General Information**

The following is general information other than the "protocol title, number, version number, and date", and change of such information will not involve the update of protocol version number and date.

**Name and address of sponsor**

| **Sponsor name** | **Sponsor address** |
| --- | --- |
| Qilu Pharmaceutical Co., Ltd. | 8888 Lvyou Road, Jinan, Shandong, China |

**Personnel authorized by the sponsor to sign and revise the protocol**

| **Name** | **Affiliation** | **Position** | **Responsibility** |
| --- | --- | --- | --- |
|  |  |  |  |
|  |  |  |  |

**Sponsor's medical expert**

| **Name** | **Affiliation** | **Position** | **Address** | **Tel.** |
| --- | --- | --- | --- | --- |
|  |  |  |  |  |

**Information of participating sites and investigators**

| **S/N** | **Site name** | **Site address** | **Tel.** | **Investigator name** | **Investigator title** | **Investigator position** |
| --- | --- | --- | --- | --- | --- | --- |
| 01 |  |  |  |  |  |  |

**Information of other participating institutions**

| **S/N** | **Institution name** | **Institution address** |
| --- | --- | --- |
| 01 |  |  |
| 02 |  |  |

**Sponsor's Signature Page**

I have read and confirmed the clinical trial protocol (protocol No.: QL1706-201, version No.: 1.2, version date: Jul. 08, 2021). I agree to carry out relevant duties in accordance with related Chinese laws and regulations, the Declaration of Helsinki, the GCP, and this protocol.

**Sponsor:** Qilu Pharmaceutical Co., Ltd.

|  |  |  |
| --- | --- | --- |
| Study Director (Print) | Study Director (Signature) | Date (MM/DD/YYYY) |

**Principal Investigator's Signature Page (Leading Site)**

I have read and confirmed the clinical trial protocol (protocol No.: QL1706-201, version No.: 1.2, version date: Jul. 08, 2021). I agree to carry out relevant duties in accordance with related Chinese laws and regulations, the Declaration of Helsinki, the GCP, and this protocol.

**Study Site:**

| Principal Investigator (Print) |  | Principal Investigator (Signature) |  | Date (MM/DD/YYYY) |
| --- | --- | --- | --- | --- |

**Principal Investigator's Signature Page (Participating Site)**

I have read and confirmed the clinical trial protocol (protocol No.: QL1706-201, version No.: 1.2, version date: Jul. 08, 2021). I agree to carry out relevant duties in accordance with related Chinese laws and regulations, the Declaration of Helsinki, the GCP, and this protocol.

**Study Site:**

| Principal Investigator (Print) |  | Principal Investigator (Signature) |  | Date (MM/DD/YYYY) |
| --- | --- | --- | --- | --- |

**Protocol Signature Page (Statistics Contractor)**

I have read and confirmed the clinical trial protocol (protocol No.: QL1706-201, version No.: 1.2, version date: Jul. 08, 2021). I agree to carry out relevant duties in accordance with related Chinese laws and regulations, the Declaration of Helsinki, the GCP, and this protocol.

**Statistics Institution:** Qilu Pharmaceutical Co., Ltd.

| Responsible Person (Print) |  | Responsible Person (Signature) |  | Date (MM/DD/YYYY) |
| --- | --- | --- | --- | --- |

**TABLE OF CONTENTS**

[Version History/Amendment History 2](#_Toc124874461)

[SYNOPSIS 6](#_Toc124874462)

[Study Schedule 17](#_Toc124874463)

[Detailed Description of Each Test Item in the Study Schedule 22](#_Toc124874464)

[Study Window Management 24](#_Toc124874465)

[1 INTRODUCTION 26](#_Toc124874466)

[1.1 Study Background 26](#_Toc124874467)

[1.1.1 Disease background 26](#_Toc124874468)

[1.1.2 Drug background 31](#_Toc124874469)

[1.2 Potential Risks and Benefits 64](#_Toc124874470)

[2 STUDY OBJECTIVES AND ENDPOINTS 65](#_Toc124874471)

[2.1 Study Objectives 65](#_Toc124874472)

[2.1.1 Primary objective 65](#_Toc124874473)

[2.1.2 Secondary objectives 65](#_Toc124874474)

[2.1.3 Exploratory objectives 65](#_Toc124874475)

[2.2 Study Endpoints 65](#_Toc124874476)

[2.2.1 Primary endpoint 65](#_Toc124874477)

[2.2.2 Secondary endpoints 66](#_Toc124874478)

[2.2.3 Exploratory endpoints 66](#_Toc124874479)

[3 STUDY DESIGN 66](#_Toc124874480)

[4 STUDY POPULATION 67](#_Toc124874481)

[4.1 Inclusion Criteria 67](#_Toc124874482)

[4.2 Exclusion Criteria 69](#_Toc124874483)

[4.3 Subject Withdrawal Criteria 71](#_Toc124874484)

[4.4 Study Premature Termination or Discontinuntion Criteria 72](#_Toc124874485)

[5 INVESTIGATIONAL PRODUCTS 72](#_Toc124874486)

[5.1 Drug Distribution 72](#_Toc124874487)

[5.2 Compliance 73](#_Toc124874488)

[5.3 Overview of Investigational Products 73](#_Toc124874489)

[5.3.1 General information 73](#_Toc124874490)

[5.3.2 Dose regimen 74](#_Toc124874491)

[5.3.3 Supply, packaging, and labeling of investigational products 76](#_Toc124874492)

[5.3.4 Management of investigational products 76](#_Toc124874493)

[5.4 Dose Modification 76](#_Toc124874494)

[5.4.1 Dose modification for QL1706 76](#_Toc124874495)

[5.4.2 Dose modification of chemotherapeutic drugs 81](#_Toc124874496)

[5.4.3 Dose modification of bevacizumab 81](#_Toc124874497)

[5.5 Concomitant Treatments 85](#_Toc124874498)

[5.5.1 Previous medications and other treatments 85](#_Toc124874499)

[5.5.2 Concomitant medications and other treatments 85](#_Toc124874500)

[6 STUDY PROCESS 87](#_Toc124874501)

[6.1 Overview of the Study Process 87](#_Toc124874502)

[6.1.1 Imaging examination of tumor lesions 87](#_Toc124874503)

[6.2 Study Procedures at Various Time Points 87](#_Toc124874504)

[6.2.1 Study visit 87](#_Toc124874505)

[6.2.2 Re-treatment after PD confirmed by imaging 87](#_Toc124874506)

[6.2.3 Unscheduled visit 88](#_Toc124874507)

[6.3 Voluntary Withdrawal by the Subject 88](#_Toc124874508)

[6.4 Study Termination and End of Study 89](#_Toc124874509)

[6.4.1 Study termination 89](#_Toc124874510)

[7 SAFETY EVALUATION 89](#_Toc124874511)

[7.1 Adverse Event 89](#_Toc124874512)

[7.1.1 Definitions 89](#_Toc124874513)

[7.1.2 Adverse event evaluation 91](#_Toc124874514)

[7.1.3 Recording of adverse events 94](#_Toc124874515)

[7.1.4 Progressive disease 95](#_Toc124874516)

[7.1.5 Reporting of serious adverse events 95](#_Toc124874517)

[7.1.6 Safety reference documents 95](#_Toc124874518)

[7.2 Pregnancy 96](#_Toc124874519)

[8 OTHER EVALUATIONS 96](#_Toc124874520)

[8.1 Efficacy Evaluation 96](#_Toc124874521)

[8.1.1 Efficacy evaluation criteria 96](#_Toc124874522)

[8.1.2 Efficacy endpoints 96](#_Toc124874523)

[8.2 Evaluations of Pharmacokinetics and Immunogenicity 97](#_Toc124874524)

[8.2.1 Blood sampling 97](#_Toc124874525)

[8.2.2 Sample processing and storage 97](#_Toc124874526)

[8.2.3 Analysis and evaluation 97](#_Toc124874527)

[8.3 Exploratory Evaluation 97](#_Toc124874528)

[9 DATA MANAGEMENT 97](#_Toc124874529)

[10 STATISTICAL ANALYSIS 99](#_Toc124874530)

[10.1 Sample Size Determination 99](#_Toc124874531)

[10.2 Statistical Analysis Datasets 99](#_Toc124874532)

[10.3 Statistical Analysis Plan 100](#_Toc124874533)

[10.3.1 General analysis 100](#_Toc124874534)

[10.3.2 Management of missing data 100](#_Toc124874535)

[10.3.3 Summary of study completion 100](#_Toc124874536)

[10.3.4 Safety analysis 100](#_Toc124874537)

[10.3.5 Efficacy analysis 101](#_Toc124874538)

[10.3.6 Evaluation of pharmacokinetics/pharmacodynamics data 101](#_Toc124874539)

[10.3.7 Immunogenicity analysis 101](#_Toc124874540)

[10.3.8 Other analyses 102](#_Toc124874541)

[10.3.9 End of study 102](#_Toc124874542)

[11 INFORMED CONSENT, ETHICAL STANDARDS, AND REGULATIONS 102](#_Toc124874543)

[11.1 Informed Consent 102](#_Toc124874544)

[11.2 Ethical Standards 103](#_Toc124874545)

[11.3 Regulations 103](#_Toc124874546)

[12 STUDY MANAGEMENT 104](#_Toc124874547)

[12.1 Quality Control and Assurance 104](#_Toc124874548)

[12.2 Document Storage 104](#_Toc124874549)

[12.3 Publication of Study Results 105](#_Toc124874550)

[13 RISK CONTROL 105](#_Toc124874551)

[13.1 Measures for Minimizing Conventional Risks 105](#_Toc124874552)

[13.2 Possible Important Risks and Risk Minimization Measures 107](#_Toc124874553)

[13.2.1 Possible important risks 107](#_Toc124874554)

[14 REFERENCES 110](#_Toc124874555)

[15 APPENDICES 112](#_Toc124874556)

[15.1 Appendix 1 Eastern Cooperative Oncology Group Performance Status
(ECOG PS) Score 113](#_Toc124874557)

[15.2 Appendix 2 New York Heart Association (NYHA) Functional Classification 114](#_Toc124874558)

[15.3 Appendix 3 Cockcroft-Gault Equation and Body Surface
Area Calculation Formula 115](#_Toc124874559)

[15.4 Appendix 4 Response Evaluation Criteria in Solid Tumors Version 1.1
(RECIST 1.1) 116](#_Toc124874560)

[15.5 Appendix 5 Comparison between iRECIST and RECIST 1.1 131](#_Toc124874561)

**List of Abbreviations**

| **Abbreviation** | **Definition** |
| --- | --- |
| ADA | Anti-drug Antibody |
| AE | Adverse Event |
| ALT | Alanine Aminotransferase |
| ANC | Absolute Neutrophil Count |
| AST | Aspartate Aminotransferase |
| BEV | Bevacizumab |
| BUN | Blood Urea Nitrogen |
| CBC | Complete Blood Count |
| CxDy | Cycle x Day y |
| CI | Confidence Interval |
| C_max_ | Maximum Observed Plasma Concentration |
| CrCl | Creatinine Clearance |
| ccRCC | Clear Cell Renal Cell Carcinoma |
| CD | Cluster of Differentiation |
| CNS | Central Nervous System |
| CR | Complete Response |
| CRA | Clinical Research Associate |
| CT | Computed Tomography |
| CTCAE | Common Terminology Criteria for Adverse Events |
| CTLA-4 | Cytotoxic T lymphocyte-associated antigen-4 |
| DCR | Disease Control Rate |
| DLT | Dose-Limiting Toxicity |
| DOR | Duration of Response |
| ECG | Electrocardiogram |
| ECOG | Eastern Cooperative Oncology Group |
| eCRF | Electronic Case Report Form |
| EDC | Electronic Data Capture |
| FcRn | Fc receptor |
| FIH | First-in-Human |
| GCP | Good Clinical Practice |
| h | hour(s) |
| HBsAg | Hepatitis B Surface Antigen |
| HC | Heavy Chain |
| HCV | Hepatitis C Virus |
| HED | Human Equivalent Dose |
| HIV | Human Immunodeficiency Virus |
| IB | Investigator's Brochure |
| ICF | Informed Consent Form |
| ICH | The International Council for Harmonisation of Technical Requirements for Pharmaceuticals for Human Use |
| IFNγ | Interferon gamma |
| Ig | Immunoglobulin |
| IgG | Immunoglobulin G |
| IL | Interleukin |
| irAE | Immune-Related Adverse Event |
| IRB | Institutional Review Board |
| IV | Intravenous |
| LC | Light Chain |
| MABEL | Minimally Anticipated Biologic Effect Level |
| MAD | Maximum Administered Dose |
| MedDRA | Medical Dictionary for Regulatory Activities |
| MRI | Magnetic Resonance Imaging |
| MRSD | Maximum Recommended Starting Dose |
| MTD | Maximum Tolerated Dose |
| NCI | National Cancer Institute |
| NMPA | National Medical Products Administration |
| NOAEL | No Observed Adverse Effect Level |
| NSCLC | Non-Small-Cell Lung Carcinoma |
| ORR | Objective Response Rate |
| PAD | Pharmacologically Active Dose |
| PBMC | Peripheral Blood Mononuclear Cell |
| PD | Pharmacodynamic |
| PD | Progressive disease |
| PD-1 | Programmed cell death protein 1 |
| PD-L1 | Programmed cell death–ligand 1 |
| PFS | Progression-free Survival |
| PFS6 | Progression-free Survival 6 months |
| PK | Pharmacokinetic |
| PR | Partial Response |
| QTcF | QT interval corrected for heart rate according to Fridericia’s formula |
| Q3W | Every three weeks |
| RBC | Red Blood Cell |
| RECIST | Response Evaluation Criteria for Solid Tumors |
| RP2D | Recommended Phase 2 Dose |
| SAE | Serious Adverse Event |
| SAP | Statistical Analysis Plan |
| SD | Stable Disease |
| SOP | Standard Operating Procedure |
| SUSAR | Suspected Unexpected Serious Adverse Reaction |
| T_1/2_ | Elimination half-life |
| TEAE | Treatment-Emergent Adverse Event |
| TMB | Tumor Mutation Burden |
| TERE | Treatment-Related Adverse Event |
| TNFα | Tumor Necrosis Factor alpha |
| TSH | Thyroid Stimulating Hormone |
| ULN | Upper Limit of Normal |
| WBC | White Blood Cell |

SYNOPSIS

| Study title | Clinical study of the safety and efficacy of QL1706 in combination with chemotherapy in first-line treatment of patients with Stage IIIB/C and Stage IV non-small cell lung cancer |
| --- | --- |
| Protocol No. | QL1706-201 |
| Version No. | 1.2 |
| Sponsor | Qilu Pharmaceutical Co., Ltd. |
| Leading site | Sun Yat-Sen University Cancer Center |
| Principal investigator |  |
| Investigational drug | QL1706 |
| Study objectives | **Primary objective**  To evaluate the safety of QL1706 combined with chemotherapy ± bevacizumab in patients with advanced non-small cell lung cancer (NSCLC).  **Secondary objectives**   1. To evaluate the efficacy of QL1706 combined with chemotherapy ± bevacizumab in patients with NSCLC. 2. To evaluate the pharmacokinetic (PK) characteristics of QL1706 combined with chemotherapy ± bevacizumab in patients with NSCLC. 3. To evaluate the immunogenicity of QL1706 combined with chemotherapy ± bevacizumab in patients with NSCLC.   **Exploratory objectives**   1. To explore the efficacy as per iRECIST. 2. To explore the correlation between certain biomarkers and the anti-tumor activity of QL1706. 3. To explore the correlation between QL1706 exposure and clinical efficacy, safety, and immunogenicity in patients with advanced NSCLC. |
| Study endpoints | **Primary endpoint**  Safety endpoints: AE, TEAE occurrence, vital signs, and laboratory test parameters.  **Secondary endpoints**   1. Objective response rate (ORR, per RECIST v1.1). 2. Progression-free survival (PFS, per RECIST v1.1). 3. Duration of response (DOR, per RECIST v1.1). 4. Disease control rate (DCR, per RECIST v1.1). 5. Overall survival (OS). 6. 1-year OS. 7. The PK characteristics of QL1706 combined with chemotherapy ± bevacizumab. 8. The immunogenicity of QL1706 combined with chemotherapy ± bevacizumab.   **Exploratory endpoints**   1. The ORR/PFS/DOR/DCR per iRECIST. 2. To explore whether PD-L1 and TMB are predictive biomarkers of clinical efficacy. 3. The correlation between QL1706 exposure and clinical efficacy, safety, and immunogenicity in patients with advanced NSCLC. |
| Study population | Patients with advanced NSCLC |
| Sample size | This study plans to enroll 60 subjects, namely 20 to Cohort 1 and 40 to Cohort 2. Sample size estimation is not based on statistical assumptions. During the study, the number of subjects enrolled in each cohort will be promptly adjusted according to the efficacy and safety signals of each regimen. |
| Study design | This is a single-arm, non-randomized, open-label, multi-center phase II clinical study to investigate the safety and efficacy of QL1706 combined with chemotherapy ± bevacizumab in first-line treatment of patients with advanced NSCLC.  This study consists of two cohorts: Cohort 1: advanced squamous NSCLC, dosing: QL1706 + paclitaxel + carboplatin; Cohort 2: advanced non-squamous NSCLC, dosing: QL1706 + pemetrexed + carboplatin ± bevacizumab.  The study plans to enroll 20 subjects to Cohort 1 and 40 subjects to Cohort 2. Twenty subjects are enrolled directly to Cohort 1; In Cohort 2, 3 subjects are first enrolled to receive QL1706 + pemetrexed + carboplatin and are subject to safety observation from the start of dosing to the end of Cycle 1 (21 days after the first dose). Adverse events (AEs) are evaluated by investigators as per NCI-CTCAE 5.0. When all first enrolled subjects completed Cycle 1 treatment, safety and tolerability can be confirmed if the following drug-related AEs are reported among < 1/3 of the subjects only. Thereafter, 3 more subjects can be enrolled to receive QL1706 + bevacizumab + pemetrexed + carboplatin and are subject to safety observation for 4-drug combination regimen. Similarly, safety and tolerability can be confirmed if the following drug-related AEs are reported among < 1/3 of the subjects only. Following discussion and confirmation by investigator and sponsor, more subjects can be enrolled to receive the combination of QL1706 + bevacizumab + pemetrexed + carboplatin, until 40 subjects are enrolled to Cohort 2, including at least 20 EGFR-positive subjects. If drug-related AEs are reported in ≥ 1/3 of the subjects, three other subjects will be enrolled for further evaluation of safety and tolerability, or dose adjustment or study termination will be considered for safety following discussion between investigator and sponsor. During the study, the number of subjects enrolled in each cohort will be promptly adjusted according to the efficacy and safety data of each treatment regimen.  Tolerability of combination therapy will be evaluated based on the occurrence of the following drug-related AEs:   - Grade ≥ 3 non-hematological adverse reactions (excluding transient electrolyte abnormality, diarrhea, nausea, and vomiting that could resolve to grade 2 or below within 3 days after best supportive care, and asthenia that could resolve to grade 2 or below within 7 days after best supportive care) and grade ≥ 2 cardiac insufficiency; - Grade 4 thrombocytopenia or grade 3 thrombocytopenia with significant clinical hemorrhagic diathesis, grade 4 neutropenia that persists for ≥ 7 days or grade 3 neutropenia with pyrexia ≥ 38.3 °C; - Other grade 4 hematological toxicities; - Other circumstances of subject intolerance assessed by the investigator.   Clinical tumor imaging evaluation will be performed once every 6 weeks (± 7 days) as per RECIST v1.1/iRECIST. From 48 weeks after the first dose, clinical tumor imaging evaluation will be performed once every 12 weeks (± 7 days). If the subjects develop PD (PD) or have no clinical benefits during the dosing period, treatment is ended and the subjects enter the follow-up period.  The subjects will receive study treatment until PD (judged by the investigator as per RECIST v1.1/iRECIST), with no clinical benefits, or meeting other withdrawal criteria for subjects, whichever occurs first. The subjects will receive treatment with QL1706 for 2 years in maximum.  The subjects who develop PD (PD per RECIST v1.1 is currently iUP per iRECIST) may continue treatment with the investigational drug if, the subjects are clinically stable and the benefits of continuing treatment outweigh the risks according to the investigator's judgment. PD should be confirmed by imaging examination (with an interval of ≥ 4 weeks and ≤ 8 weeks from the previous imaging examination as per iRECIST). Treatment with QL1706 should be discontinued in case of confirmed PD (iCPD as per iRECIST criteria). Note: Chemotherapeutic drugs and bevacizumab should be discontinued at the first occurrence of PD. |
| Dose regimen | All investigational products are administered via intravenous infusion, every 21 days for a cycle, once on the first day of each cycle (Q3W, D1). If the full dose cannot be completed on the first day, the remaining drug can be administered on the next day. Treatment regimens are detailed as follows:   \| **Treatment group** \| **Histologic type** \| **Run-in period** \| **Post run-in period** \| \| --- \| --- \| --- \| --- \| \| Cohort 1 \| Squamous NSCLC \| QL1706 (5 mg/kg) + paclitaxel (175 mg/m^2^) + carboplatin (AUC5/6), 4 cycles \| QL1706 (5 mg/kg) \| \| Cohort 2 \| Non-squamous NSCLC \| QL1706 (5 mg/kg) + pemetrexed (500 mg/m^2^) + carboplatin (AUC5/6) ± bevacizumab (15 mg/kg),  4 cycles \| QL1706 (5 mg/kg) + pemetrexed  (500 mg/m^2^) ± bevacizumab  (15 mg/kg) \| |
| Subject population | **Inclusion criteria**  Subjects must meet all of the following inclusion criteria to be eligible for study participation:   1. Subjects voluntarily participate in the study and sign the ICF. 2. Subjects are aged ≥ 18 years when signing the ICF, with no gender restrictions. 3. Eastern Cooperative Oncology Group (ECOG) performance status score of 0–1. 4. Expected survival time ≥ 3 months. 5. Histologically- or cytologically-confirmed Stage ⅢB/C or IV NSCLC (classified according to 8th edition of AJCC) patients, including squamous and non-squamous cancer. Details can be found below. 6. Cohort 1: Stage ⅢB/C (unsuitable for radical surgery or other radical local therapy) and Stage IV squamous NSCLC patients, without previous systemic anti-cancer treatment for Stage IIIB/C or metastatic disease. Those who have previously received neoadjuvant/adjuvant therapy for non-metastatic disease, or radical chemoradiotherapy, may be enrolled if the last treatment is > 6 months from the start of study treatment. 7. Cohort 2: Stage IIIB/C (unsuitable for radical surgery or other radical local therapy) and Stage IV non-squamous NSCLC patients, without previous systemic anti-cancer treatment for Stage IIIB/C or metastatic disease. Those who have previously received neoadjuvant/adjuvant therapy for non-metastatic disease, or radical chemoradiotherapy, may be enrolled if the last treatment is > 6 months from the start of study treatment. But the following are excluded: (1) patients carrying sensitive EGFR gene mutation should meet any of the following conditions: experiencing no exon 20 T790M mutation after failing (treatment failure: occurrence of radiographic progression or toxicity intolerance during treatment or after treatment completion as judged by the investigator as per RECIST v1.1) the first or second-generation EGFR-TKI therapies (including gefitinib, erlotinib, icotinib, afatinib, etc.); experiencing exon 20 T790M mutation after failing the first or second-generation EGFR-TKI monotherapy, followed by recurrence of PD or toxicity intolerance after receiving osimertinib or other third-generation EGFR-TKI therapies; failing the prior osimertinib or other third-generation EGFR-TKI therapies (as first-line treatment) (regardless of EGFR T790M mutation status). If the subjects show other sensitive mutations (except exon 19 deletion mutation and exon 21 L858R mutation), including 18G719X, 20S786I, and 21L861Q mutations, apart from the aforementioned conditions, subjects should also have failed EGFR-TKI treatment after previous response (per Jackman criteria: CR/PR or SD ≥ 6 months) to EGFR-TKI treatment. (2) Patients carrying anaplastic lymphoma kinase (ALK)-fused oncogene have to experience PD or be intolerant to ALK-TKI during or after ALK-TKI (e.g., crizotinib) therapy. Note: TKI wash-out period for all participants is 1 week or 5 half-lives after the last treatment, whichever is longer. 8. Patients with at least one imaging measurable lesion per RECIST v1.1. Tumor lesions with clear radiographic progression after previous radiotherapy (or other local treatments) are also considered measurable lesions. 9. Cohort 2 subjects are required to provide test reports of EGFR/ALK and other genes prior to enrollment. 10. Organs must exhibit adequate functional level prior to the first dose of study treatment (transfusion with any blood components and drugs that stimulate the growth of WBC or platelets are not allowed within 7 days before obtaining laboratory test results). 11. WBC ≥ 3 × 10^9^/L. 12. Absolute neutrophil count ≥ 1.5 × 10^9^/L. 13. Platelet count ≥ 100 × 10^9^/L. 14. Hemoglobin ≥ 90 g/L. 15. Serum creatinine ≤ 1.5 × ULN or creatinine clearance (CLcr) ≥ 50 mL/min calculated according to the Cockcroft-Gault equation. 16. Total bilirubin ≤ 1.5 × ULN (< 3 × ULN is permitted for patients with Gilbert's syndrome). 17. AST and ALT ≤ 2.5 × ULN (≤ 5 × ULN is permitted for patients with liver metastases). 18. INR or APTT ≤ 1.5 × ULN. 19. Left ventricular ejection fraction (LVEF) > 50%. 20. Subjects (both female and male) must employ effective contraceptive measures from the signing of the ICF to 180 days after the last dose of the investigational products. Women who are pregnant or lactating (who can be enrolled if she agrees to stop breastfeeding during this period) from the signing of the ICF to 180 days after the last dose of the investigational products.   **Exclusion criteria**  Subjects who meet any of the following criteria will not be enrolled in this study.   1. CNS metastases with symptoms. Subjects may be enrolled if they fulfill the following conditions at least 2 weeks prior to the first dose of study treatment: Adequate treatment of CNS metastases and resolution of nervous system symptoms (other than residual signs and symptoms related to CNS treatment), and discontinuation or dose reduction of systemic corticosteroids (≤ 10 mg prednisone per day or equivalent). 2. Presence of carcinomatous meningitis prior to the first dose of study treatment. 3. Known active autoimmune disorder that requires systemic treatment within 2 years prior to the first dose of study treatment. But the following are excluded: related replacement therapies (such as thyroxine, insulin, or physiological corticosteroid replacement therapy for renal/pituitary insufficiency); skin diseases not requiring systemic treatment, such as vitiligo and psoriasis. 4. Diseases that require treatment with systemic corticosteroids (> 10 mg prednisone per day or equivalent) or other immunosuppressive drugs (cyclophosphamide, azathioprine, methotrexate, thalidomide, TNF-alpha inhibitors, etc.) within 2 weeks prior to the first dose of study treatment. Local corticosteroids, nasal spray, and inhalational steroids are allowed. Systemic corticosteroids as prophylaxis for contrast media allergy are allowed. Low-dose corticosteroids for orthostatic hypotension are allowed. 5. Concurrent diseases that may seriously affect patient safety or impact the completion of the study as determined by the investigator, such as hypertension that cannot be controlled by 2 or more types of anti-hypertensive drugs (systolic blood pressure ≥ 160 mmHg and/or diastolic blood pressure ≥ 100 mmHg) and severe diabetes. 6. Subjects with any of the following cardiovascular diseases shall be excluded. 7. Acute myocardial infarction within 6 months prior to the first dose of the study treatment or patients with NYHA class III or IV heart failure. 8. Poorly uncontrolled cardiovascular disease prior to the first dose of the study treatment, including angina, pulmonary arterial hypertension, or severe arrhythmia or conduction disorders. 9. 12-lead ECG shows that mean QT interval (QTcF) > 450 ms (males) or > 470 ms (females) prior to the first dose of the study treatment. 10. Patients with previous or current interstitial lung disease, pneumoconiosis, radiation pneumonia, or severe lung function impairment, which may interfere with the detection and treatment of suspected treatment-related lung toxicities deemed by the investigator. 11. History of intestinal fistula, gastrointestinal perforation, or intra-abdominal abscess within 6 months prior to screening. 12. Uncontrolled pleural and peritoneal effusions, or pericardial effusion requiring repeated drainage (≥ 1 drainage/month). 13. Uncontrolled or symptomatic hypercalcemia. 14. Grade ≥ 2 peripheral neuropathy (CTCAE v5.0) prior to the first dose of study treatment. 15. Previous immunotherapies, including immune checkpoint inhibitory antibodies (anti-PD-1, PD-L1, CTLA-4 antibodies, etc.), agonistic antibodies (anti-ICOS, CD40, CD137, GITR, OX40, etc.), immune cell therapy, etc. 16. Severe hemorrhagic diathesis or coagulation dysfunction, or currently receiving anticoagulant therapy or thrombolytic therapy. Imaging shows signs of tumor invasion into large vessels [tumor has completely approached, surrounded, or invaded lumen of large vessels (such as pulmonary artery or superior vena cava)]. Two or more occurrences of hemoptysis [≥ 0.5 teaspoon (2.5 mL) per occurrence] within 3 months prior to screening. Thrombosis within 6 months prior to screening or excessive arterial/venous thrombosis, such as cerebrovascular accidents (including transient ischemic attack), deep vein thrombosis, pulmonary embolism within 1 year prior to screening. 17. Scheduled to receive anti-RANKL therapy [such as denosumab and its biosimilars] during the study treatment period. 18. Received > 30 Gy chest/lung radiotherapy within 6 months before the first study treatment; received palliative radiotherapy at other parts or other local therapies within 2 weeks before the first study treatment, and have not recovered from adverse reactions due to radiotherapy or other local therapies. 19. Underwent any surgery within 4 weeks before the first study treatment and have not recovered. 20. History of allogeneic hematopoietic stem cell transplantation or organ transplantation (except corneal transplantation). 21. Systemic infection or other serious infection requiring intravenous antibiotic treatment for > 7 days within 14 days before the first study treatment. 22. HIV-positive patients; patients known to have received tuberculostatic therapy within one year prior to initial study treatment; HBsAg-positive patients having HBV DNA at a level ≥ 2000 IU/mL or 10^4^ copies/mL; HCV antibody-positive and HCV RNA-positive patients (Note: HCV antibody-positive patients should be ruled out if HCV RNA could not be detected in the study site; in addition, if the results of HBsAg, HBV DNA, HCV antibody, and HCV RNA assays fail to meet the aforementioned requirements because the study site fails to perform qualitative/quantitative assays, then the aforementioned corresponding criteria would be met when values of these items are out of normal reference ranges in the study site and judged by the investigator to be clinically significant abnormalities). 23. Received live attenuated vaccine within 4 weeks before the first study treatment. However, scheduled inactivated vaccine during the study treatment period is allowed. 24. Patients with a known history of psychoactive drug abuse, alcohol abuse, or substance abuse; history of definitive neurological or mental disorder, including epilepsy and dementia. 25. Patients with other malignant tumors within 5 years before the first study treatment (except cured basal cell carcinoma of the skin, cervical carcinoma *in situ*, and papillary thyroid carcinoma). 26. Patients with a known history of allergy to macromolecular protein drug products or to QL1706 and any component of other investigational products. 27. Patients who participated in other clinical studies and used other investigational drugs within 28 days before the first study treatment. 28. Patients with added risks associated with the study or who may interfere with the interpretation of study results as determined by the investigator, or who are deemed unsuitable by the investigator and/or sponsor.   **Subject withdrawal criteria**  A subject may withdraw from the study for any of the following situations:   1. Voluntary withdrawal from the study by the subject. 2. The subject develops intolerable adverse reactions and the investigator believes that the study treatment shall be discontinued. 3. The subject experiences PD (as determined by the investigator according to RECIST v1.1/iRECIST). 4. The subject completed 2 years of QL1706 treatment. 5. The subject commits severe protocol deviation and the investigator and/or sponsor believe that treatment should be discontinued. 6. Female subjects who become pregnant during the study treatment period. 7. Other reasons lead the investigator to believe that the subject is unsuitable to further study treatment. 8. The subject starts other new anti-cancer treatment. 9. The study discontinues.   **Study premature termination or discontinuation criteria**   1. The regulatory authority, Institutional Review Board, sponsor, or investigator believes that there is a major safety risk with the investigational drug. 2. The sponsor may terminate this study due to scientific, medical, or ethical reasons but must fully consider the rights, safety, and health of enrolled subjects. 3. Other reasons that the sponsor or investigator determines to be unsuitable for study continuation. 4. The regulatory authority requests for trial termination due to other reasons. |
| Statistical analysis | The primary objective of this study is to evaluate the safety of the combination therapies, and its secondary objectives are to observe the efficacy and pharmacokinetic properties of the combination therapies, and to conduct immunogenicity study.  **Analysis of safety endpoints:**  Adverse events will be coded according to MedDRA and their incidences are evaluated. Events will be classified according to their severity, into treatment-emergent adverse event (TEAE)/reactions, serious adverse events/reactions, and adverse events/reactions leading to dropout or death. And their numbers and incidences will calculated accordingly. All adverse events will be listed and described.  The clinical laboratory markers, vital signs, physical examinations, and ECG examination results will be summarized and analyzed according to the observed values at the study visit and the changes from baseline. And the results of clinical significance judgment of laboratory tests will be summarized and analyzed in the form of a cross table involving pre-treatment and post-treatment values.  **Analysis of secondary endpoints:**  The number and percentage of subjects with objective response (ORR) will be calculated, and Clopper-Pearson method will be used to calculate the 95% confidence interval (CI). DCR will be analyzed using the same analysis method for ORR. Kaplan-Meier method will be used to estimate mPFS, mDOR, and mOS and the corresponding 95% CI, and survival curves will be plotted.  Plasma concentration of QL1706 obtained in this study will be pooled with concentration data from other studies to construct a QL1706 population pharmacokinetic model using a nonlinear mixed-effects modeling for exploratory analysis. Population pharmacokinetic analysis will then be applied to evaluate potential correlation between QL1706 exposure and efficacy, adverse events, and immunogenicity.  The number and percentage of subjects who are ADA-positive and Nab-positive will be summarized. For ADA-positive subjects, descriptive analysis of their antibody titer test results and changes relative to baseline will be carried out further. ADA and Nab results of all subjects will be tabulated. |
| Study schedule | Expected enrollment date of the first subject: Sep. 2021  Expected enrollment date of the last subject: Mar. 2022  Expected end date of the study: Jan. 2023 |

Study Schedule

| **Item/Schedule** | **Screening period/Baseline**★ | | **Treatment period**▲ | | **Post-Treatment** | | | |
| --- | --- | --- | --- | --- | --- | --- | --- | --- |
|  | Within 28 days before the first dose | Within 7 days before the first dose | C1 | C2+ | End of treatment^[p][q]^ | 30 days after the last dose of the investigational products | 90 days after the last dose of the investigational products | Long-term follow-up |
|  |  |  | D1 | D1 |  |  |  |  |
| Baseline data |  |  |  |  |  |  |  |  |
| Signing of the ICF | **×** |  |  |  |  |  |  |  |
| Demographic data | **×** |  |  |  |  |  |  |  |
| Medical history and treatment history | **×** |  |  |  |  |  |  |  |
| Tissue and blood sample collection^[a]^ | **×** |  |  |  |  |  |  |  |
| General examinations/Scoring |  |  |  |  |  |  |  |  |
| Body height^[b]^ |  | **×** |  |  |  |  |  |  |
| Body weight^[c]^ |  |  | **×** | **×** | **×** |  |  |  |
| Vital signs^[d]^ |  | **×** | **×** | **×** | **×^[r]^** |  |  |  |
| Comprehensive physical examination^[e]^ |  | **×** |  |  |  |  |  |  |
| Targeted physical examination^[e]^ |  |  |  | **×** | **×^[r]^** |  |  |  |
| ECOG performance status |  | **×** |  | **×** | **×^[r]^** |  |  |  |
| Laboratory tests^[g]^ |  |  |  |  |  |  |  |  |
| Urinalysis |  | **×** |  | **×** | **×^[r]^** |  |  |  |
| Hematology |  | **×** |  | **×** | **×^[r]^** |  |  |  |
| Blood biochemistry |  | **×** |  | **×** | **×^[r]^** |  |  |  |
| Hemorrhage and coagulation |  | **×** |  |  | **×^[r]^** |  |  |  |
| Cardiac enzyme profile |  | **×** |  |  | **×^[r]^** |  |  |  |
| Thyroid function |  | **×** |  | **×^[f]^** | **×^[r]^** |  |  |  |
| Pituitary-adrenal axis |  | **×** |  | **×^[f]^** | **×^[r]^** |  |  |  |
| Infectious diseases | **×** |  |  |  |  |  |  |  |
| Other examinations/assessments^[g]^ |  |  |  |  |  |  |  |  |
| 12-Lead ECG |  | **×** |  | **×** | **×^[r]^** |  |  |  |
| Echocardiography | **×** |  |  |  |  |  |  |  |
| Pregnancy test^[h]^ |  | **×** |  |  | **×**^[r]^ |  |  |  |
| Enrollment confirmation |  |  |  |  |  |  |  |  |
| Confirmation of inclusion/exclusion criteria^[i]^ |  | **×** |  |  |  |  |  |  |
| Imaging examination/assessment of tumors during treatment period |  |  |  |  |  |  |  |  |
| Imaging examination of tumor lesions^[j]^ | **×** |  |  | **×** |  |  |  |  |
| Immunogenicity and pharmacokinetics |  |  |  |  |  |  |  |  |
| Immunogenicity blood sampling^[k]^ |  |  | **×** | | **×**^[r]^ | **×**^[q]^ |  |  |
| PK blood sampling^[k]^ |  |  | **×** | | **×**^[r]^ | **×**^[q]^ |  |  |
| Investigational products/concomitant medications/AE |  |  |  |  |  |  |  |  |
| QL1706 infusion^[l]^ |  |  | **×** | **×** |  |  |  |  |
| Infusion of chemotherapeutic drugs and bevacizumab^[l]^ |  |  | **×** | **×** |  |  |  |  |
| Recording of concomitant medications/other treatments^[m]^ | **×** | **×** | **×** | **×** | **×** | **×** | **×** |  |
| Recording of adverse events^[m]^ | **×** | **×** | **×** | **×** | **×** | **×** | **×** |  |
| Long-term follow-up |  |  |  |  |  |  |  |  |
| Imaging examination of tumor lesions^[n]^ |  |  |  |  | **×**^[s]^ | **×** | | |
| Survival follow-up^[o]^ |  |  |  |  |  | **×** | | |
| Anti-cancer treatment status^[o]^ |  |  |  |  |  | **×** | | |

★: Before signing of the ICF, relevant evaluations (including imaging examinations, laboratory tests, ECG, and echocardiography) that are routinely performed can be used for screening if they are completed at this study site within the protocol-specified time window and are protocol-compliant in other factors.

▲: Before the investigational products are given, safety evaluation results should be obtained and assessed by investigators to determine the feasibility of continued treatment. In case of dosing delay, the corresponding examinations/tests should be delayed (except the imaging examination of tumors).

1. Tissue and blood sample collection: Past tumor paraffin blocks or 10–15 pathological biopsy sections (unstained) should be collected from subjects; blood should also be collected concurrently as the control (before the first study treatment). If tissues cannot be obtained, the blood is not required.
2. Body height: After baseline height measurement, the height may not be measured before dosing if not required.
3. Body weight: Body weight is measured before each dose of the investigational products. If dose modification occurs due to weight changes, the weight after dose modification would be used as the new baseline weight.
4. Vital signs: See "Study Window Management" for details.
5. Physical examination: Comprehensive physical examination shall be conducted at screening. Targeted physical examination may be performed based on symptoms after screening.
6. Thyroid function and pituitary-adrenal axis: Examinations will be performed once per cycle before QL1706 dosing during the first 4 cycles of study treatment, and every 2 cycles before QL1706 dosing thereafter.
7. Laboratory tests and other examinations/assessments: Multiple measurements are allowed during the screening period, and the most recent data before the first study treatment will be used as the screening period/baseline data.
8. Pregnancy test: Women of childbearing age will undergo a serum pregnancy test. Additional tests may conducted subsequently based on clinical requirements.
9. Confirmation of inclusion/exclusion criteria: Study treatment will be started after confirming subjects who meet all inclusion criteria and do not meet any exclusion criteria.
10. Imaging examination of tumor lesions: Identical imaging methods should be used for baseline and subsequent evaluations. Tumor imaging examination conducted due to routine diagnosis and treatment before signing of the ICF can be used for baseline tumor evaluation if conducted within 28 days before the first dose of the investigational products at the same study site in accordance with the requirements, and there is no need for a re-examination unless the investigator suspects that tumor load has changed. Imaging examination of lesions will be performed at the specified frequency after initiation of study treatment (once every 6 weeks from the date of the first study treatment until Week 48, and every 12 weeks thereafter, unless the frequency needs to be modified as determined by the investigator; for patients with brain metastases at baseline, imaging examination of brain metastases will be performed once every 12 weeks; bone scans will be performed at the frequency per the investigator's judgement), until PD as per RECIST v1.1 (or iCPD as per iRECIST), death, loss to follow-up, withdrawal of informed consent, start of new anti-cancer treatment, or end of study (whichever occurs first).
11. See "Study Window Management" for details.
12. Infusion of QL1706, chemotherapeutic drugs, and bevacizumab: Different chemotherapy regimens will be selected according to different histological types. Chemotherapy will be given for 4 cycles, while bevacizumab will be given according to the study design and dose regimen.
13. Recording of concomitant medications/other treatments and adverse events: The duration of recording of concomitant medications/other treatments and that of adverse event collection will last from the signing of the ICF to 90 days after the last dose of the investigational products or the start of new anti-cancer treatment (whichever occurs first). See relevant sections in the protocol for details.
14. Imaging examination of tumor lesions: Subsequent imaging examinations of the lesion for subjects who discontinue study treatment not for PD (RECIST v1.1) should be carried out according to the following procedure as far as possible until PD (RECIST v1.1), death, loss to follow-up, withdrawal of informed consent, start of new anti-cancer treatment, 12 months after the last dose of the investigational products, or end of study, whichever occurs first: (1) If imaging examination of the lesion is not carried out at the end-of-treatment visit, an imaging examination of the lesion should be carried out according to the original schedule as far as possible; (2) If imaging examination of the lesion is carried out at the end-of-treatment visit, imaging examination of the lesion should be carried out subsequently once every 6 weeks until Week 48 and every 12 weeks thereafter.
15. Survival follow-up, anti-cancer treatment status: Subjects who discontinue study treatment not for PD (RECIST v1.1) should undergo imaging examination of the lesion based on the above criteria [n] as far as possible. Then, they should undergo follow-up once every 12 weeks for collection of subsequent anti-cancer treatment information and survival status until death, 12 months after the last dose of the investigational products, or end of study, whichever occurs first. Subjects who discontinue study treatment due to PD (PD as per RECIST v1.1 or iCPD as per iRECIST) will undergo follow-up once every 12 weeks after completing the end-of-treatment visit for collection of subsequent anti-cancer treatment information and survival status until death, loss to follow up, 12 month after the last dose of the investigational products, or end of study, whichever occurs first.
16. The end of treatment includes the investigator's decision for a subject to discontinue study treatment and a subject's request to withdraw from the study. The end of treatment date is determined by the date in which the investigator decides for the subject to discontinue all investigational products and the date of withdrawal due to the subject's decision, respectively. The end-of-treatment visit should be carried out within 7 days after the end of treatment, and the procedures and examinations listed in the table should be completed as much as possible.
17. If a subject has started other new anti-cancer treatment before this, the corresponding procedures/examinations will be deemed unnecessary.
18. Need not be carried out if completed within the last 3 days.
19. An imaging examination of the lesion is not required at the end-of-treatment visit if the last imaging examination was conducted less than 4 weeks ago.

Detailed Description of Each Test Item in the Study Schedule

| **Item** | **Requirements/Observation parameters** | |
| --- | --- | --- |
| Informed Consent | The written ICF must be obtained first from the subject before any procedure of the clinical trial. | |
| Demographic data | Date of birth, sex, ethnicity, etc. | |
| Medical history and treatment history | Family history of tumor, lung cancer history and treatment history, allergy history, and other past/current medical history, etc. | |
| Tissue and blood sample collection | Past tumor paraffin blocks or 10–15 pathological biopsy sections (unstained) should be collected from subjects; blood should also be collected concurrently as the control (before the first study treatment). If tissues cannot be obtained, the blood is not required.  Recommendations for tumor tissue collection: Fresh tissues (preferred) should be obtained before the first dose, or tumor tissue sections obtained within 6 months before the first dose. Systemic therapy (such as adjuvant or neoadjuvant chemotherapy) cannot be performed after sample is obtained. Fine-needle aspiration biopsy or the use of pleural effusions is considered insufficient for biomarker evaluation. Biopsy of bone tumor samples with no soft tissue components or decalcification should also be avoided. | |
| Vital signs | Blood pressure, pulse, respiration, and body temperature | |
| Physical examination | Comprehensive physical examination should be conducted at screening, involving the skin, superficial lymph nodes, head, neck, chest/back, abdomen, anus, limbs, and nervous system (may be different due to varied descriptions by various hospitals)  Targeted physical examination may be performed based on symptoms after screening | |
| Body height | Body height will be measured at the stipulated time point. After the first measurement, it may not be measured again if not required | |
| Body weight | Body weight will be measured at the stipulated time points | |
| ECOG performance status | ECOG performance status will be scored at the stipulated time points | |
| Laboratory tests | Urinalysis | Specific gravity, pH, glucose, protein, ketone bodies, white blood cells, and red blood cells |
|  | Hematology | Red blood cell count, hemoglobin, hematocrit, white blood cell count, neutrophil count, lymphocyte count, eosinophil count, basophil count, monocyte count, and platelet count. |
|  | Blood biochemistry | Alanine aminotransferase, aspartate aminotransferase, alkaline phosphatase, lactate dehydrogenase, γ-glutamyl transferase, lipase, amylase, total protein, albumin, total bilirubin, direct bilirubin, creatinine, urea/urea nitrogen, uric acid, potassium, sodium, calcium, magnesium, phosphorus, chloride, and glucose |
|  | Hemorrhage and coagulation | Prothrombin time, activated partial thromboplastin time, D-dimer, and international normalized ratio |
|  | Cardiac enzyme profile | Troponin I, troponin T, creatine kinase (CK), creatine kinase isoenzyme (CK-MB), α-hydroxybutyrate dehydrogenase, and myoglobin (tests can be selected based on actual circumstances of each site) |
|  | Thyroid function | Free triiodothyronine [FT3], free thyroxine [FT4], and thyroid stimulating hormone [TSH] |
|  | Pituitary-adrenal axis | Adrenocorticotropic hormone [ACTH], cortisol, follicle-stimulating hormone [FSH], luteinizing hormone [LH], testosterone, and prolactin |
|  | Infectious diseases | Hepatitis B panel (HBsAg, HBsAb, HBeAg, HBeAb, and HBcAb) and HBV DNA (HBV DNA may only be tested when HBsAg is positive or exceeds the normal range), HCV antibody and HCV RNA (HCV RNA may only be tested when HCV antibody is positive or exceeds the normal range), HIV antibody, and syphilis antibody |
| 12-Lead ECG | Standard 12-lead ECG will be carried out for 3 consecutive runs at every time point, with a between-run interval of 5 min (± 2 min) | |
| Echocardiography | Echocardiography, including left ventricular ejection fraction | |
| Pregnancy test | Women who may become pregnant will undergo serum pregnancy test | |
| Imaging examination of tumor lesions | At baseline: Brain, chest, abdominal (including adrenals), and pelvic CT/MRI examinations will be performed. If clinically indicated, appropriate methods, such as a bone scan, should be employed to examine other known or suspected lesion sites.  Post-baseline: Examination sites should at least include the chest and abdomen (including adrenals). All lesions found at baseline or subsequently suspected new lesions should be examined based on the lesion sites. Unscheduled imaging examinations may be conducted if PD is suspected or deemed necessary by the investigator. | |
| Immunogenicity blood sampling | Blood will be sampled at the specified time points for immunogenicity analysis | |
| PK blood sampling | Blood will be sampled at the specified time points for PK analysis | |

Study Window Management

1. **Time window for dosing**

Time window for dosing: Every 21 days (± 3 days) after the first dose of QL1706, chemotherapeutic drugs, or bevacizumab.

Time window for each dosing of QL1706, chemotherapeutic, or bevacizumab is calculated from the date of their first dose. Generally, dosing can be brought forward or delayed by no more than 3 days. Under special circumstances (such as public holidays), it can be brought forward/delayed by more than 3 days after the reason is specified. The next dosing time should be postponed if dosing is brought forward/delayed by more than 3 days.

1. **Other visit time windows**

End of treatment: Same as the time window for various examinations

30/90 days after the last dose: ± 7 days

Long-term follow-up: ± 7 days

1. **Dose window for investigational products administration**

The dose window is ± 5%.

The dose needs not to be adjusted when the change in body weight of the subject is within ± 10%.

1. **Tumor imaging examination time window**

**Treatment period and post-treatment**

Imaging examination of lesions: after the first dose of the investigational products, with the examination time window of ± 7 days.

1. **Time window for various examinations**

**Treatment period**

Body weight, physical examination, ECOG performance status: From the first dose of the investigational products onwards, the measurement/evaluation time window is within 3 days before dosing of the investigational products. If possible, body weight should be measured as close as possible to the dosing date.

Vital signs: Vital signs will be evaluated during the first dose of the investigational products on C1D1, before QL1706 infusion (−30 min), at the end of infusion (+ 5 min), 1 h (± 10 min) after the end of infusion, and 4 h (± 10 min) after the end of infusion, and when necessary. In subsequent dosing days, vital signs will be evaluated before (−30 min) and at the end of QL1706 infusion (+ 5 min) and when necessary.

Laboratory tests and 12-lead ECG: From the first dose of the investigational products onwards, the measurement time window is within 3 days before dosing of the investigational products. 12-lead ECG will be performed for 3 consecutive runs at every time point, with a between-run interval of 5 min ± 2 min.

**Post-Treatment**

**End-of-treatment visit**

Various safety examinations: −3 to + 7 days.

1. **Blood sampling time windows for immunogenicity and pharmacodynamics testing**

**Table 1. Blood sampling time windows for immunogenicity and pharmacodynamics testing**

| **Item/Schedule** | **C1/2/3/4^[a]^** | **C5+^[a]^** | **End of treatment** | **30 days after the last dose of the investigational products** |
| --- | --- | --- | --- | --- |
| **Time point** | **D1 pre-dose** | **D1 pre-dose** |  |  |
| **Window period** | −1 h | −1 h | Same as the time window for various examinations | ± 7 days |
| **Immunogenicity** | **×** | **×** | **×** | **×**^[b]^ |
| **Pharmacokinetics** | **×** | **×** | **×** | **×**^[b]^ |

1. Blood will be sampled before the first 4 doses of QL1706 and thereafter every 4 dosing cycles (e.g., C8D1, C12D1, etc.) before dosing of QL1706. In case of QL1706 dosing delay, the corresponding sampling should be delayed.
2. Subjects, where possible, will be asked to return to the hospital for blood sampling 30 days after the last dose of the investigational products. Failure to do so will not be considered as protocol deviation.

**MAIN TEXT**

# INTRODUCTION

## Study Background

### Disease background

Overview

Lung cancer ranks first in terms of incidence and mortality among patients with malignant tumors in China. Statistics from WHO's Global Cancer Observatory showed that there were approximately 816,000 new cases of lung cancer and approximately 715,000 related deaths in China in 2020^[1]^. The 5-year survival rate for lung cancer is only 19%^[2]^. Cigarette smoking is the number one risk factor for lung cancer as the smoke contains a variety of carcinogenic chemicals (e.g., nitrosamines and benzo[a]pyrene diol epoxide). Based on biological characteristics, treatment, and prognosis, lung cancer is generally classified by WHO into two main categories: non-small cell lung cancer (NSCL) and small cell lung cancer (SCLC). NSCLC accounts for 85% of all lung cancers^[3]^ and can be divided into two main pathological types: 1) squamous cell (epidermoid) carcinoma; 2) non-squamous cell carcinoma (including adenocarcinoma, large cell carcinoma, and other subtypes). Adenocarcinoma is the most common histological type of NSCLC among non-smokers, as well as a common histological subtype carrying driver gene mutation. Although lung cancer mortality has been reduced with advances in screening, minimally invasive diagnostic technology, radiotherapy, targeted therapy, and immunotherapy in recent years, it still ranks first among all malignant tumors.

1. Immune escape mechanisms in tumor

1.1 Normal immune surveillance in the body

Under normal physiological status, the immune system has a "surveillance function" that accurately identifies "non-self" components; it can eliminate invading microbes, reject allografts, and detect and destroy tumor cells. In-depth immunology studies have proved that T lymphocyte-mediated immunity is the main mechanism of anti-tumor immunity. Neoantigens produced by tumor cells are ingested and processed by antigen presenting cells (APCs), and presented in the form of antigen peptide-major histocompatibility complex (MHC) class I/II molecules to T cells by APCs, enabling T cells to be activated, proliferated, and differentiated into tumor antigen-specific cytotoxic T cells (CTLs). Then CTLs migrate and infiltrate the tumor bed, and specifically recognize and kill tumor cells through the interaction between T cell receptors (TCRs) and antigen peptide-MHC class I molecules. After the apoptosis of tumor cells, more tumor-associated antigens will be released, which activate more T cells to further kill the tumor^[4]^.

1.2 Immune escape mechanisms in tumor and corresponding therapeutic strategies

Under normal condition, the body's immune surveillance function can recognize and eliminate cancerous cells, but tumor cells evade the immune system's surveillance in a variety of ways, eventually leading to the occurrence and development of tumors. Immune escape mechanisms in tumor can be roughly described from the following three aspects^[5]^: (1) Immune checkpoint: In addition to expressing some specific antigens recognized by the immune system, tumor cells express a variety of immunosuppressive ligands, which are bound to inhibitory receptors expressed by T cells, such as programmed cell death protein 1 (PD-1), LAG-3, TIM-3, TIGIT, VISTA, and CD244. These co-inhibitory molecules inhibit T cell function. The inhibition of T cell activation and proliferation can be removed by blocking the co-inhibitory signal, thus killing tumor cells^[5]^. (2) Loss of antigenicity: Tumor cells evade recognition by the immune system by losing the expression of specific antigens, thus avoiding the surveillance of the immune system. Immune cells can be modified by cell engineering technology to recognize other specific "non-self" antigens on the surface of tumor cells and kill tumor cells^[6,7]^. (3) Immunosuppressive microenvironment: In solid tumor tissues, there are a variety of negatively regulated cells and cytokines, which together constitute the immunosuppressive microenvironment around the tumor tissues. Immune cells and their secreted cytokines can be regulated to promote angiogenesis and other factors involved in the tumor microenvironment, thus achieving immunotherapy^[8]^.

2. Immune checkpoint inhibitors (ICIs)

When T cells are activated, the expression of immune checkpoints, including PD-1/programmed death-ligand 1 (PD-L1) and cytotoxic T lymphocyte-associated antigen-4 (CTLA-4), in the corresponding co-inhibitory signaling pathways increases, whereas, ICIs restore or enhance the body's anti-tumor immunity by blocking these checkpoints^[9]^.

2.1 PD-(L)1 inhibitors

PD-1 is an important immunosuppressive transmembrane protein expressed on the surface of T cells, and its main ligand is PD-L1. Tumor cells can express PD-L1, which is bound to PD-1, leading to tyrosine phosphorylation in the cytoplasmic domain of PD-1 and recruitment of tyrosine phosphatase SHP-2. They further result in the dephosphorylation of TCR signaling molecules, weakening the activation of downstream signals of TCR and reducing T cell activation and cytokine production^[10]^. PD-(L)1 inhibitors restore the body's immune killing function to tumor cells by blocking the binding of PD-1/L1.

2.2 CTLA-4 inhibitor

CTLA-4, a transmembrane protein encoded by the CTLA-4 gene, is expressed on activated CD4+ and CD8+ T cells and bound to ligands CD80 (B7-1) and CD86 (B7-2). CTLA-4 reduces TCR and CD28 signaling and inhibits T cell activation by competitively inhibiting the binding of CD28 to B7 ligand or recruiting phosphatase to the cytoplasmic domain of CTLA-4. Moreover, CTLA-4 downregulates the expression of CD80/CD86 on APCs through cytokines, or causes the removal of CD80/CD86 from APCs by endocytosis, thus reducing the binding of CD28 to B7 ligand. Furthermore, CTLA-4 inhibits T cell response by binding CD80/CD86 expressed by dendritic cells and inducing the expression of tryptophan-degrading enzyme indoleamine 2,3-dioxygenase (IDO)^[10]^. At present, no anti-CTLA-4 antibody has been approved for marketing in China, while the anti-CTLA-4 antibody approved outside China is ipilimumab (trade name "Yervoy") for advanced NSCLC etc.

3. Progress of combination immunotherapy in the first-line treatment for driver gene-negative NSCLC

For driver gene-negative advanced NSCLC patients, the median PFS and median OS are only 4–6 months and 10–12 months, respectively, after traditional platinum-doublet chemotherapy^[11-14]^, while immunotherapy can bring them survival benefits.

Pembrolizumab

KEYNOTE-189^[15,16]^ was a phase III randomized trial comparing the efficacy of pemetrexed/carboplatin (or cisplatin) with or without pembrolizumab for the treatment of metastatic non-squamous NSCLC without EGFR mutation or ALK fusion. After a median follow-up of 10.5 months, the median PFS was 8.8 months in the immunotherapy combined with chemotherapy group and 4.9 months in the chemotherapy monotherapy group. The overall survival was improved in the two groups regardless of the PD-L1 expression level. Based on these data, NCCN guidelines have recommended pembrolizumab combined with chemotherapy as the preferred first-line treatment regimen for metastatic non-squamous NSCLC patients without EGFR, ALK, BRAF V600E, METex14 skipping, RET, and ROS1 mutations.

In KEYNOTE-407^[17]^ study, the efficacy of paclitaxel/carboplatin with or without pembrolizumab for the treatment of metastatic squamous NSCLC patients was compared. The results showed that the median OS was 15.9 months in the immunotherapy combined with chemotherapy group and 11.3 months in the chemotherapy monotherapy group. The ORR was 57.9% and 38.4% in the combination therapy and chemotherapy groups, respectively. The incidence of discontinuation due to AEs was higher in the immunotherapy combined with chemotherapy group than in the chemotherapy group, 13.3% and 6.4%, respectively. Based on these data, NCCN guidelines have recommended pembrolizumab combined with paclitaxel and carboplatin (Category 1; preferred) as a first-line treatment option for metastatic squamous NSCLC patients without EGFR, ALK, BRAF V600E, METex14 skipping, RET, and ROS1 mutations.

Atezolizumab

In the randomized phase III IMPower150^[18]^ trial, the first-line ABCP regimen (PD-L1 + BEV + chemotherapy) was compared with BCP regimen (BEV + chemotherapy) in patients with metastatic non-squamous NSCLC. The results showed that the median OS was 19.2 months in the ABCP group and 14.7 months in the BCP group. The PFS in the ABCP group was longer than that in the BCP group, with the difference statistically significant (8.3 m vs. 6.8 m). Based on these clinical data, the NCCN panel has recommended ABCP regimen (also known as the 4-drug regimen) as a first-line treatment option (Category 1; other recommended intervention) for metastatic non-squamous NSCLC patients without EGFR, ALK, ROS1, METex14 skipping, RET, or BRAF mutation. Maintenance treatment with atezolizumab and bevacizumab is also recommended in this case.

Nivolumab ± ipilimumab

Nivolumab and ipilimumab are ICIs with complementary mechanisms of action on T cells. Nivolumab is a fully human monoclonal antibody against the PD-1 receptor. Ipilimumab is a human cytotoxic T lymphocyte-associated antigen-4 (CTLA-4) blocking antibody.

CheckMate 227^[19]^ was a phase III randomized trial, in which nivolumab/ipilimumab, nivolumab monotherapy, and chemotherapy were compared in metastatic non-squamous or squamous NSCLC patients without EGFR mutation or ALK fusion and whose PS was 0–1 and PD-L1 expression levels were ≥ 1%. For patients with PD-L1 expression ≥ 1%, the median OS was 17.1 months in the nivolumab/ipilimumab group and 14.9 months in the chemotherapy group.

In the phase III randomized trial CheckMate 9LA^[20]^, the efficacy of nivolumab + ipilimumab combined with 2 cycles of platinum-doublet chemotherapy was compared with 4 cycles of chemotherapy monotherapy as the first-line treatment for metastatic non-squamous or squamous NSCLC patients without EGFR mutation or ALK fusion. The chemotherapy regimens were pemetrexed + cisplatin or carboplatin for non-squamous NSCLC and paclitaxel + carboplatin for squamous NSCLC. Preliminary efficacy data showed a median OS of 15.6 months in the dual-immunotherapy combined with chemotherapy group and 10.9 months in the chemotherapy monotherapy group, regardless of histology or PD-L1 expression levels. The ORR was 38% and 25% in the dual-immunotherapy combined with chemotherapy group and chemotherapy monotherapy group, respectively. The incidence of grade 3/4 AEs was higher in the dual-immunotherapy combined with chemotherapy group than in the chemotherapy monotherapy group, 25.4% and 15%, respectively. Based on these data, the NCCN panel has recommended nivolumab/ipilimumab/chemotherapy as a first-line treatment option (Category 2A; other recommended intervention) for metastatic NSCLC patients who meet the criteria.

4. Progress of combination immunotherapy in the second-line and later-line treatments for EGFR-positive NSCLC

The most common EGFR mutations in NSCLC patients are exon 19 deletion and exon 21 point mutation. Both mutations lead to activation of the tyrosine kinase domain and are associated with the sensitivity to small molecule EGFR-TKI. Therefore, these drug-sensitive EGFR mutations are referred to as sensitive EGFR mutations. They are detected in approximately 10% of Caucasian NSCLC patients and up to 50% of Asian patients^[21]^. Patients typically experience progression after first-line EGFR-TKI monotherapy, and EGFR p.Thr790Met (T790M) is a mutation associated with acquired resistance to EGFR-TKI therapy. Osimertinib is recommended for EGFR T790M-positive NSCLC patients who have experienced progression after treatment with erlotinib, gefitinib, afatinib, and dacomitinib. However, there is no ideal treatment regimen for patients who have experienced re-progression.

In the phase III IMPower150^[22]^ study, some NSCLC patients with EGFR mutation or ALK fusion who experienced progression (or intolerance) during prior TKI therapy (n = 108) were enrolled, although the majority of patients (87%) in this trial did not have these gene variations. For these patients with EGFR mutation or ALK fusion, the PFS in the ABCP group was longer than that in the BCP group (9.7 m vs. 6.1 m). A subgroup analysis of IMPower150 reported that the ABCP regimen as the later-line treatment extended the median OS of some patients with EGFR mutation-positive metastatic NSCLC (n = 34) compared with carboplatin plus paclitaxel plus bevacizumab in patients (n = 45). Therefore, the ABCP regimen may be an option for patients with EGFR mutation or ALK fusion who have experienced progression after initial TKI therapy.

Bevacizumab combined with chemotherapy has been approved for the treatment of metastatic non-squamous NSCLC. In addition to the known anti-angiogenic effect, bevacizumab inhibits vascular endothelial growth factor (VEGF) and has an immunomodulatory effect. The addition of bevacizumab reverses VEGF-mediated immunosuppression, which can enhance the efficacy of ICIs.

Therefore, a prospective trial is required to evaluate the safety and clinical efficacy of first-line immunotherapy combined with chemotherapy in patients with driver gene NSCLC and immunotherapy combined with chemotherapy ± bevacizumab in patients with EGFR mutation-positive non-squamous NSCLC who have experienced progression after TKI therapy.

### Drug background

Qilu Puget Sound Biotherapeutic Corporation (a wholly-owned subsidiary of Qilu Pharmaceutical Group) used the advanced MabPair^TM^ biotechnology platform to develop a new therapeutic biological product targeting PD-1 and CTLA-4. The R&D code is QL1706 (formerly known as PSB205). The product is composed of two engineered monoclonal antibodies (anti-programmed cell death protein 1 [PD-1] and anti-cytotoxic T lymphocyte-associated antigen-4 [CTLA-4]) expressed in a fixed ratio, and is produced by a single cell line as a single product.

QL1706 targets and inhibits two immune checkpoint pathways (anti PD-1 immunoglobulin G[IgG]4 and anti-CTLA-4 IgG1). Bispecific antibodies can cover two targets equally. The two-antibody combination in a MabPair product (such as QL1706) can provide pharmacokinetic (PK) distribution and antibody binding effect with significant target-specific binding level. For example, in QL1706, the anti-PD-1 component is immunoglobulin IgG4, and the anti-CTLA-4 component is IgG1 isotype antibody. For QL1706, the anti-CTLA-4 IgG1 isotype component may be a key factor for reducing regulatory T cell function in the tumor microenvironment. In the construction of QL1706 biological structure, anti-PD-1 IgG4 and anti-CTLA-4 IgG1 have also been modified to exhibit different PK properties *in vivo*, achieving an optimal balance between therapeutic efficacy and toxicity.

Comprehensive non-clinical studies and evaluations have been performed for QL1706 and its components. In *in v*i*tro* and *in vivo* evaluations, QL1706 and its anti-PD-1 and anti-CTLA-4 components significantly increase environment-dependent activation and function of T cells. In humanized tumor-bearing mice, QL1706-mediated antineoplastic response was higher than that mediated by anti-PD-1 or anti-CTLA-4 component alone. In humanized mouse models, QL1706 can also induce systemic proliferation of human T cells. Although the two components of QL1706 are biologically active, the antigen-dependent immune stimulation of QL1706 was observed to be different from that of each of its individual components. There is synergism between the two components.

Many tumor models have proved that combination therapy of nivolumab and ipilimumab has better antineoplastic efficacy compared with monotherapy. This combination therapy has been approved in countries outside China for first-line treatment of advanced melanoma, first-line treatment of advanced renal carcinoma with moderate-to-high risk, and third-line treatment of advanced MSI-H/dMMR colorectal cancer.

While combination therapy with anti-PD-(L)1 and anti-CTLA-4 increases efficacy, its toxicity also significantly rises compared with that of monotherapy. The maximum tolerated dose for nivolumab could not be determined in a 0.1–20 mg/kg Q2W dose escalation study in patients with solid tumors, but the incidence of potential immune-related AEs (irAEs) was similar among different doses^[23][24]^. irAEs observed when a combination of nivolumab and ipilimumab were used and increased patient withdrawal may be related to high ipilimumab exposure^[25]^. When examining how not to affect the efficacy of the combination therapy while increasing the safety and decreasing the number of patients who withdrew from the study due to intolerable toxicity, study personnel decreased ipilimumab dose and dosing frequency, increased dosing interval, and used 3 mg/kg Q2W nivolumab combined with low dose ipilimumab (1 mg/kg Q6W or Q12W) to treat patients with advanced non-small cell lung cancer^[26]^. The results showed that decreasing anti-CTLA-4 antibody exposure at the clinically effective dose of anti-PD-1 antibody would not decrease the efficacy of the combination therapy but could increase the tolerability of patients towards the combination therapy.

In order to achieve different levels of PD-1 and CTLA-4 target coverage, a relative dose (ratio) of anti-PD-1 and anti-CTLA-4 in QL1706 of 2:1 was selected. The allometric PK model was used to confirm the ratio of QL1706 components and this ratio was fixed as 2:1 based on the model. This simulation predicts that the anti-PD-1 and anti-CTLA-4 antibody exposure levels are similar to nivolumab Q3W combined with ipilimumab Q6W when QL1706 is dosed at 3-week intervals. The simulation results also showed that predicted target coverage could be achieved if the QL1706 ratio fell within 10% of the fixed ratio of anti-PD-1:anti-CTLA-4.

Therefore, a fixed ratio of QL1706-specific components can be used to test the proposed clinical hypothesis, i.e., the clinical antineoplastic efficacy of QL1706 is at least similar to that reported for the combination therapy of nivolumab/ipilimumab, and the product is capable of decreasing the number of patients who discontinue treatment due to serious irAEs, enabling patients to be treated for a longer period.

#### Physicochemical and pharmaceutical properties and formulation

QL1706 is a bifunctional antibody produced in a single cell line, consisting of anti-PD-1 (aPD-1, named PSB103) antibody and anti-CTLA-4 (aCTLA-4, named PSB105) antibody. Both antibodies are specifically modified by cell engineering technology and able to be expressed in a single cell line in a predetermined ratio. The following table lists the general properties of the components.

Table 2. General properties of anti-PD-1 IgG4 and anti-CTLA-4 IgG1

| **Anti-PD-1 IgG4** | |
| --- | --- |
| **Property** | **Result** |
| Measured mass of aPD-1 (Dalton) | 149,313 ± 4 Da (the main peak was measured by LC-MS, and the G0F/G0F glycan was matched. The N-terminal Q in two heavy chains was converted to pyro-E, and the C-terminal K was removed) |
| Measured deglycosylation mass of aPD-1 (Dalton) | 146,424.2 Da (mean mass; the N-terminal Q in two heavy chains was converted to pyro-E, and the C-terminal K was removed. N296 was transformed to D296 after deglycosylation) |
| Isoelectric point | 7.5 (main peak) |
| Extinction coefficient | 1.64 (experimental) |
| Number of disulfide bonds | 16 |
| Number of glycosylation sites | 2 (1 in each heavy chain) |
| **Anti-CTLA-4 IgG1** | |
| **Property** | **Result** |
| Measured mass of aCTLA-4 (Dalton) | 147,608 ± 4 Da (the main peak was measured by LC-MS, and the G0F/G0F glycan was matched. The N-terminal Q in two heavy chains was converted to pyro-E, and the C-terminal K was removed) |
| Measured deglycosylation mass of aCTLA-4 (Dalton) | 144,718.9 Da (mean mass; the N-terminal Q in two heavy chains was converted to pyro-E, and the C-terminal K was removed. N298 was transformed to D298 after deglycosylation) |
| Isoelectric point | 9.0 (main peak) |
| Extinction coefficient | 1.65 (experimental) |
| Number of disulfide bonds | 18 |
| Number of glycosylation sites | 2 (1 in each heavy chain) |

The humanized anti-huPD-1 antibody is an IgG4 antibody with two identical HCs and two identical LCs; human anti-huCTLA-4 antibody is an IgG1 antibody with two identical HCs and two identical LCs. Mutation modification was performed on the two antibodies to promote proper assembly and prevent mismatch. L-Serine at position 228 in the hinge region of HC of anti-PD-1 IgG4 antibody was replaced by L-proline to prevent Fab arm exchange. L-Arginine at position 255 of HC of anti-CTLA-4 IgG1 antibody was replaced by L-lysine to reduce the binding to FcRn and improve the clearance.

QL1706 is composed of anti-PD-1 IgG4 and anti-CTLA-4 IgG1 in the ratio of 65:35 ± 10% (anti-PD-1:anti-CTLA-4), with excipients of acetate-sucrose (10 mM sodium acetate, 9% sucrose, and 0.02% polysorbate 20 (W/V), at pH 5.0). Its dosage form is liquid and route of administration is intravenous. QL1706 is stored under refrigeration condition (2–8 °C).

#### Preclinical studies

##### Pharmacodynamics

###### *In vitro* pharmacodynamics

The ability of QL1706 and its anti-PD-1 component (PSB103) to block the interaction between PD-1 and PD-L1 was evaluated by the dual-cell reporter assay. PSB103 and QL1706 had similar IC50 (50% inhibitory concentration) potency (1.6 nM and 1.7 nM, respectively), while the control IgG4 showed no functional blocking activity. QL1706 and PSB103 had comparable inhibitory activity, and the activity was directly proportional to the molecular weight of PSB103.

The ability of QL1706 and its anti-CTLA-4 component (PSB105) to block the interaction between CTLA-4 and B7-1/B7-2 was evaluated by the dual-cell reporter assay. PSB105 and QL1706 had similar IC50 potency (3.78 nM and 4.12 nM, respectively), while the control IgG1 showed no blocking activity. QL1706 and PSB105 had comparable inhibitory activity, and the activity was directly proportional to the molecular weight of PSB105.

**PSB103 enhancing T cell activation in human allogeneic MLR:** PSB103 exhibited a higher potency compared with the observed nivolumab response (EC50 [50% effective concentration] of 6.56 nM and 0.71 nM, respectively), with about a 15-fold increase in IFNγ production (EC50 = 0.45 nM) and a 5-fold increase in CD4+ T cell proliferation level (EC50 = 0.13 nM). PSB103 and its equivalent pembrolizumab had the similar degree of response in inducing IFNγ production or CD4+ T cell proliferation, with EC50 of 0.85 nM and 0.14 nM, respectively. The responses observed at a human IgG concentration of 3000 ng/mL were similar to those examined at the lowest concentration for PSB103, nivolumab, or pembrolizumab.

**QL1706 and its components enhancing T cell activation in human Allo-MLR:** PSB103 alone induced a 50% increase in CD8+ CMV+ T cells, while QL1706 induced a 3-fold increase in CD8+ CMV+ T cells. PSB105 induced a slight increase (5%) compared with the control huIgG. Therefore, QL1706 can mediate the synergism when both PD-1 and CTLA-4 pathways are blocked in this system.

**QL1706 and its components enhancing SEB-induced T cell activation:** Based on the maximum IL-2 production induced by SEB, the effect of QL1706 was nearly 2 times that of PSB105 and 5 times that of PSB103. The potency of QL1706 (EC50 = 2.1 nM) was about 50 times that of PSB105 (EC50 = 119 nM) and more than 100 times that of PSB103 (EC50 > 200 nM). IL-2 production induced by QL1706 was higher than the sum of that induced by PSB105 and PSB103, indicating a synergism of the two components in the assay system.

**ADCC activity assessment:** QL1706 cannot mediate cells expressing PD-1 and CTLA-4 to produce ADCC activity.

**CDC activity assessment:** QL1706 and PSB105 mediate cells expressing CTLA-4 to produce CDC, but no CDC activity was detected in target cells expressing PD-1 only after induction by component PSB103.

###### *In vivo* pharmacodynamics

Four studies were conducted to investigate the *in vivo* pharmacodynamics and mechanism of action of QL1706, including the *in vivo* pharmacodynamic study of QL1706 in B-h4-1BB transgenic mouse MC38 tumor model, the study on the mechanism of action of QL1706 in B-h4-1BB transgenic mouse MC38 tumor model, the *in vivo* pharmacodynamic study of QL1706 monotherapy and the combination therapy of QL1706 with QL1604 (an anti-human PD-1 monoclonal antibody developed by Qilu Pharmaceutical Co., Ltd.) in B-hPD-1/h4-1BB double-transgenic mouse MC38 tumor model, and the *in vivo* pharmacodynamic study of QL1706 monotherapy and the combination therapy of QL1706 with QL1604 on human HT-29 tumor in the PBMC humanized NOG model. The above studies' results showed that QL1706 could inhibit the growth of MC38 mouse colon cancer and HT-29 human colon cancer, showing certain dose dependency. The mechanism study of QL1706 in h4-1BB transgenic mouse MC38 model was consistent with the 4-1BB target and *in vitro* mechanism of action. It was proved on the two tumor models of MC38 and HT-29 that the combination therapy of QL1706 with QL1604 monoclonal antibody had better efficacy than QL1706 monotherapy, and no toxicity differences in mortality rate, body weight, and signs and symptoms were observed between the combination therapy and monotherapy groups.

##### *In vivo* pharmacology

Tumor models selected for *in vivo* pharmacology included non-small cell lung cancer (HCC827), mantle cell lymphoma (Jeko-1), and Jeko-1 xenograft models. Cumulative anti-tumor efficacy of QL1706 showed in all models. See the figures below for details.


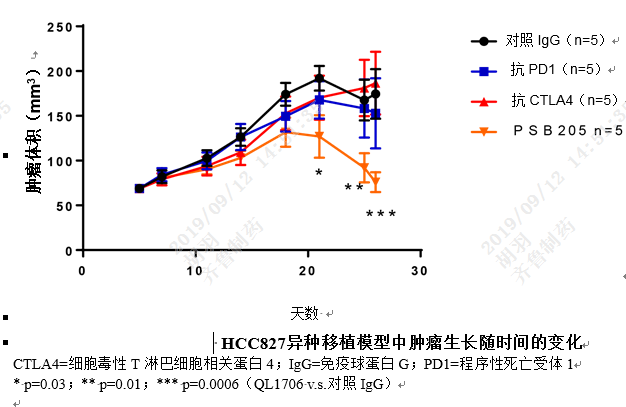

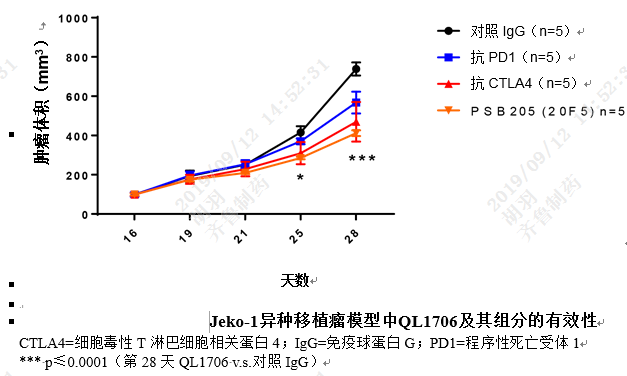


**Day(s)**

**Tumor volume (mm^3^)**

Control IgG (n = 5)

Anti-PD1 (n = 5)

Anti-CTLA4 (n = 5)

**Efficacy of QL1706 and its components in Jeko-1 xenograft model**

CTLA4 = cytotoxic T lymphocyte-associated antigen 4; IgG = immunoglobulin G;
PD1 = programmed cell death protein 1

***p ≤ 0.0001 (QL1706 vs. control IgG on Day 28)

**Tumor volume (mm^3^)**

Control IgG (n = 5)

Anti-PD1 (n = 5)

Anti-CTLA4 (n = 5)

**Day(s)**

**Change in tumor growth over time in HCC827 xenograft model**

CTLA4 = cytotoxic T lymphocyte-associated antigen 4; IgG = immunoglobulin G;
PD1 = programmed cell death protein 1

*p = 0.03; **p = 0.01; ***p = 0.0006 (QL1706 vs. control IgG)


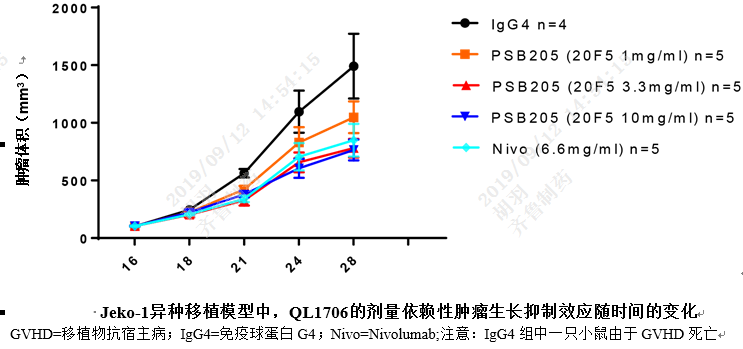


**Tumor volume (mm^3^)**

**Change in inhibitory effect of QL1706 on dose-dependent tumor growth over time in Jeko-1 xenograft model**

GVHD = graft-versus-host disease; IgG4 = immunoglobulin G4; Nivo = nivolumab; note: one mouse in the IgG4 group died from GVHD

##### Pharmacokinetic study

###### Single-dose pharmacokinetics in cynomolgus monkeys

Two single-dose exploratory trials evaluated the PK properties of PSB103 and PSB105 (two components of QL1706) after intravenous doing in cynomolgus monkeys who were not first dosed but confirmed no prior known exposure to monoclonal antibody. In the repeated-dose toxicology study in blank cynomolgus monkeys, the PK properties of QL1706 were characterized after intravenous injection.

In the exploratory non-GLP single-dose PK study, a single dose of 5 mg/kg PSB103 (29102) or human IgG4 (16102) was given to four blank cynomolgus monkeys (one male and one female in each group) by intravenous bolus. PK blood of each animal were sampled before dosing and 0.083, 0.5, 2, 8, 16, 24, 72, 144, 240, 336, 504, and 672 h after dosing. The relevant mean PK parameters by sex were assessed using the non-compartmental analysis (NCA) model of WinNonlin 6.1, and the results are summarized in the table below.

Table 3. Mean values of pharmacokinetic parameters of test article after single intravenous doing in cynomolgus monkeys of both sexes

| **Test article** | **Parameter** | **t_1/2_**  **(h)** | **T_max_**  **(h)** | **C_max_**  **(µg/mL)** | **AUC_0–last_**  **(h·µg/mL)** | **AUC_0-∞_**  **(h·µg/mL)** | **V_z_**  **(mL/kg)** | **Cl**  **(mL/h/kg)** |
| --- | --- | --- | --- | --- | --- | --- | --- | --- |
| **PSB103**  **(29102)** | N^a^ | 4 | 4 | 4 | 4 | 4 | 4 | 4 |
|  | Mean | 297 | 2.17^b^ | 204 | 37300 | 48800 | 43.9 | 0.106 |
|  | SD | 64.8 | 3.89 | 23.5 | 3540 | 10800 | 1.05 | 0.0203 |
| **Human IgG4**  **(16102)** | N^a^ | 4 | 4 | 4 | 4 | 4 | 4 | 4 |
|  | Mean | 181 | 2.06^b^ | 138 | 16900 | 21000 | 64.5 | 0.270 |
|  | SD | 51.0 | 3.96 | 19.9 | 8060 | 8420 | 9.27 | 0.108 |

IgG4 = immunoglobulin G4; SD = standard deviation

a. Two aliquots prepared for each sample. b. CV > 100%

In the second single-dose PK study, a single dose of 3 mg/kg PSB105 (10511), human IgG1 (10511P), or ipilimumab (10D1, human IgG1) was given to six blank cynomolgus monkeys (one male and one female in each group) by intravenous bolus. PK blood of each animal were sampled before dosing and 0.083 (5 min), 0.5, 2, 8, 16, 24, 72, 144, 240, 336, 504, and 672 h after dosing. The relevant mean PK parameters by sex were assessed using the NCA model of WinNonlin 6.1, and the results are summarized in the table below.

Table 4. Mean values of pharmacokinetic parameters of test article after single intravenous doing in cynomolgus monkeys of both sexes

| **Test article** | **Parameter** | **t_1/2_**  **(h)** | **T_max_**  **(h)** | **C_max_**  **(g/mL)** | **AUC_0–last_**  **(h·g/mL)** | **AUC_0-∞_ (h·g/mL)** | **V_z_**  **(mL/kg)** | **Cl**  **(mL/h/kg)** |
| --- | --- | --- | --- | --- | --- | --- | --- | --- |
| **PSB105**  **(10511)** | N | 2 | 2 | 2 | 2 | 2 | 2 | 2 |
|  | Mean | 109 | 0.290 | 116 | 6080 | 6140 | 76.6 | 0.489 |
|  | SD | 1.66 | 0.297 | 26.1 | 283 | 287 | 4.75 | 0.0229 |
| **Human IgG1**  **(10511P)** | N | 2 | 2 | 2 | 2 | 2 | 2 | 2 |
|  | Mean | 125 | 0.08 | 153 | 10100 | 10300 | 52.3 | 0.291 |
|  | SD | 21.2 | 0.00 | 4.67 | 440 | 631 | 5.69 | 0.0177 |
| **Ipilimumab** | N | 2 | 2 | 2 | 2 | 2 | 2 | 2 |
|  | Mean | 397 | 0.08 | 128 | 22500 | 31900 | 53.5 | 0.0948 |
|  | SD | 104 | 0.00 | 4.67 | 93.1 | 3740 | 7.80 | 0.0111 |

###### Repeated-dose pharmacokinetics in cynomolgus monkeys

No independent repeated-dose PK study was conducted. PK properties of QL1706 after repeated dosing were evaluated in a US-GLP-compliant repeated-dose toxicology study in blank cynomolgus monkeys. A total of 32 monkeys were randomized into 4 groups and dosed with vehicle (0) or test article (3, 15, or 60 mg/kg QL1706), Q2W, for 3 doses, by intravenous bolus on Days 1, 15, and 29. Blood for toxicokinetic (TK) analysis were sampled before each injection, 0.5, 2, 4, 24, 48, 96, 144, 216, and 336 h after the first dose, and 0.5, 2, 4, 24, 48, 96, 144, 336, 672, 1008, and 1320 h after the third/last dose. Blood for ADA analysis were sampled before dosing, on Days 15 and 29 during the dosing period, and on Days 14 and 55 after the last dose.

Table5. Toxicokinetic parameters after repeated intravenous dosing of anti-PD-1 drug to cynomolgus monkeys

| **Sex** | **Day(s)** | **Dose (mg/kg)** | **C_max_**  **(μg/mL)** | **T_last_^a^**  **(h)** | **T_max_^a^**  **(h)** | **T^1/2^**  **(h)** | **Vd_ss_ (mL/kg)** | **CL (mL/h/kg)** | **AUC_0-144 h_**  **(μg•h/mL)** |
| --- | --- | --- | --- | --- | --- | --- | --- | --- | --- |
| Male | 1^b^ | 3 | 50.3 ± 2.05 | 0.5 (0.5–0.5) | 336 (216–336) | 86.9 ± 58.8 | 72.0 ± 44.7 | 0.588 ± 0.0494 | 3080 ± 242 |
|  |  | 15 | 260 ± 17.6 | 0.5 (0.5–4) | 336 (336–336) | 119 ± ID | 95.8 ± ID | 0.557 ± ID | 15200 ± 1340 |
|  |  | 60 | 1030 ± 109 | 0.5 (0.5–2) | 336 (216–336) | 75.4 ± 2.75 | 74.2 ± 11.2 | 0.680 ± 0.0790 | 60800 ± 5250 |
|  | 29^c^ | 15 | 279 | 2 | 144 | NR | NR | NR | 15100 |
| Female | 1^b^ | 3 | 52.7 ± 3.57 | 0.5 (0.5–0.5) | 336 (216–336) | NC | NC | NC | 3020 ± 317 |
|  |  | 15 | 245 ± 29.1 | 0.5 (0.5–0.5) | 336 (336–336) | 90.0 ± ID | 81.6 ± ID | 0.702 ± ID | 13700 ± 2220 |
|  |  | 60 | 1070 ± 72.2 | 0.5 (0.5–4) | 336 (336–336) | 89.2 ± 26.4 | 65.5 ± 15.1 | 0.520 ± 0.0566 | 72100 ± 5850 |
|  | 29^c^ | 3 | 56.9 ± ID | 1.25 (0.5–2) | 144 (144–144) | NC | NC | NC | 4650 ± ID |

NA = not applicable; ID = insufficient data; NR = not reported, due to Rsq < 0.800 and/or > 20% when AUC was extrapolated to infinity; NC = not calculable

a = Median T_max_ and T_last_ (Min–Max)

b = Data of animals who were confirmed positive and negative for ADA on Day 1 (main study and recovery period); N = 3 (3 and 15 mg/kg) and N = 5 (60 mg/kg)

c = Data of ADA-negative animals on Day 29, N = 1 or 2 (3 and 15 mg/kg)

Table6. Toxicokinetic parameters after repeated intravenous dosing of anti-CTLA-4 drug to cynomolgus monkeys

| **Sex** | **Day (s)** | **Dose (mg/kg)** | **C_max_ (μg/mL)** | **T_last_^a^**  **(h)** | **T_max_^a^**  **(h)** | **T_1/2_**  **(h)** | **Vd_ss_ (mL/kg)** | **CL (mL/h/kg)** | **AUC_0-144 h_**  **(μg•h/mL)** |
| --- | --- | --- | --- | --- | --- | --- | --- | --- | --- |
| **Male** | 1^b^ | 3 | 20.7 ± 1.65 | 0.5 (0.5–2) | 216 (216–336) | 69.0 ± 40.2 | 177 ± 2.39 | 1.79 ± 0.122 | 1190 ± 99.6 |
|  |  | 15 | 98.7 ± 6.06 | 4 (2–4) | 336 (216–336) | 94.3 ± 47.6 | 208 ± 63.6 | 1.69 ± 0.538 | 6180 ± 1270 |
|  |  | 60 | 423 ± 19.8 | 0.5 (0.5–2) | 216 (216–336) | 52.4 ± 16.0 | 147 ± 34.7 | 1.98 ± 0.245 | 23500 ± 2160 |
|  | 29^c^ | 15 | 105 | 2 | 144 | 60.6 | 193 | 2.21 | 5700 |
| **Female** | 1^b^ | 3 | 19.1 ± 0.925 | 0.5 (0.5–0.5) | 336 (216–336) | 91.9 ± ID | 307 ± ID | 2.32 ± ID | 1000 ± 191 |
|  |  | 15 | 80.0 ± 4.33 | 0.5 (0.5–0.5) | 336 (216–336) | 61.9 ± 16.5 | 212 ± 54.3 | 2.40 ± 0.525 | 4780 ± 596 |
|  |  | 60 | 396 ± 18.0 | 0.5 (0.5–0.5) | 336 (216–336) | 57.6 ± 30.2 | 150 ± 81.4 | 1.78 ± 0.188 | 24400 ± 2360 |
|  | 29^c^ | 3 | 17.5 ± ID | 0.5 (0.5–0.5) | 144 (144–144) | 46.9 ± ID | 205 ± ID | 3.20 ± ID | 856 ± ID |

NA = not applicable; ID = insufficient data; NR = not reported, due to Rsq < 0.800 and/or > 20% when AUC was extrapolated to infinity

a = Median T_max_ and T_last_ (Min–Max)

b = Data of animals who were confirmed positive and negative for ADA on Day 1 (main study and recovery period); N = 3 (3 and 15 mg/kg) and N = 5 (60 mg/kg)

c = Data of ADA-negative animals on Day 29, N = 1 or 2 (3 and 15 mg/kg)

Initially, systemic exposure to anti-PD-1 and anti-CTLA-4 was achieved in all animals, and T_max_ after the first dose was 0.5–4 h. In the range of 3–60 mg/kg, C_max_ and AUC_0-144 h_ of the two analytes increased proportionally to the dose, and there was no significant sex difference after dosing on Day 1. A significant increase in clearance (CL) of both analytes was observed in male animals in the 3 and 60 mg/kg groups and female animals receiving 15 and 60 mg/kg QL1706 on Day 29 relative to Day 1. In addition, the serum analyte concentration curve declined rapidly and AUC_0-144 h_ decreased significantly, indicating possible ADA formation in these groups.

##### Toxicology studies

###### Repeated-dose toxicity

A US-GLP-compliant repeated-dose toxicity study was conducted in cynomolgus monkeys, in which the potential subchronic toxicity of QL1706 was evaluated and the reversibility, persistence, or delayed effects of the toxicity was also evaluated based on data from the 8-week recovery period. In this study, 32 monkeys (male and female animals weighing 2.2–3.9 kg) were randomized into 4 groups and dosed with vehicle (0) or test article (3, 15, or 60 mg/kg QL1706), Q2W (on Days 1, 15, and 29), for 3 doses, by intravenous bolus. On Day 35, animals, except animal 4504, in all main study groups (3 males and 3 females in each group) were euthanized and necropsied, while animals in recovery groups (2 males and 2 females in each group) were observed for another 56 days (8–week recovery period) before being euthanized on Day 85.

QL1706 dosing did not affect clinical signs, food intake, body weight, ophthalmology, electrocardiography, coagulation, and urinalysis parameters. Change in QL1706-related hematological parameters was only mild lymphocyte count decrease in male and female animals in the 60 mg/kg dose group on Day 3 (0.38 and 0.63 times lower than that before dosing, respectively). Changes in clinical biochemical parameters included slight decreases in albumin (0.80 times lower than that before dosing) and cholesterol (0.79 times lower than that before dosing) and slight increase in globulin (1.18 times higher than that before dosing) in female animals in the 60 mg/kg dose group on Day 31. Changes in albumin and globulin resulted in a decrease in albumin:globulin ratio. These changes indicated the occurrence of mild acute-phase reaction, which was probably related to local reactions stimulated by keyhole limpet hemocyanin (KLH) and not related to dosing of QL1706.

QL1706 at 3, 15, or 60 mg/kg was intravenously injected into male and female cynomolgus monkeys, Q2W, for consecutive 4 weeks (on Days 1, 15, and 29). QL1706 was well tolerated, and no adverse results were observed. One female animal (4504) was euthanized 24 h after the last dose of QL1706 due to treatment failure in an open wound. This unplanned euthanasia was not related to QL1706 treatment. Therefore, the NOAEL was determined to be 60 mg/kg.

###### Local tolerance

Local tolerance at QL1706 injection site was assessed in the pivotal 4-week repeated-dose toxicology study in cynomolgus monkeys. After cynomolgus monkeys received repeated dosing (Q2W, for 3 doses in total) at a maximum dose of 60 mg/kg (formulated at a concentration of 25 mg/mL), no clinical findings or histopathological abnormalities indicating the presence of problems of irritation or local tolerance at QL1706 injection site were observed, which were not different from those observed in animals in the vehicle group. These data revealed that neither QL1706 nor its formulation involved significant injection site irritation.

###### Immunotoxicity

Monoclonal antibody and Fc fusion protein therapeutic drugs may induce a series of acute infusion reactions that result in adverse events in the patient, including cytokine release syndrome (CRS). CRS manifestations include nausea, headache, tachycardia, hypotension, pyrexia, chills, rash, and tachypnea, and it can lead to tissue injury, organ failure, or even death. The characteristics of CRS are: elevations in TNF-α and IFNγ levels 1–2 h after the dosing of therapeutic protein followed by elevations in IL-6 and IL-10 levels.

No inflammatory cytokine release (IFN-γ, IL-2, IL-4, IL-6, IL-10, and TNF-α) was detected in the three whole blood samples from human donors after 4- or 24-h culture with QL1706 at the highest concentration of 680 nM. No cytokine release was induced after 4-h culture with QL1706 at 2040 nM. After two donor samples were cultured with QL1706 at the highest concentration of 2040 nM for 24 h, the results from one donor sample showed that immobilized QL1706 induced IL-6 and TNF-α production, while QL1706 solution induced IL-10, IL-6, and TNF-α production. In this donor sample, the levels of IL-10 and TNF-α were only several times higher than the background value, while the level of IL-6 was 20 to 100 times higher than the background value. No cytokine release was detected in another donor sample cultured with QL1706 at 2040 nM for 24 h. All the three donor samples had immobilized anti-human CD3 as the active control. After 24 h of culture, secretion of some cytokines (including IFN-γ, IL-10, IL-6, and TNF-α) was detected in each donor sample.

#### Overview of phase I clinical study

Two clinical studies have been conducted for QL1706 in China and are currently ongoing. The data as of Mar. 23, 2021 are summarized in stages.

##### QL1706-101 study

The QL1706-101 study is a phase Ia clinical study conducted in China to evaluate the tolerability, safety, pharmacokinetics, and preliminary anti-tumor activity of QL1706 in patients with advanced malignant tumors. It consists of two stages, i.e., dose escalation and PK cohort-expansion stages. Six dose groups were designed in the dose escalation stage, i.e., 0.3, 1.0, 3.0, 5.0, 7.5, and 10 mg/kg, q3w, and the study is still ongoing.

The following is a summary of the preliminary safety and efficacy data.

###### Study progress and enrollment

As of Mar. 23, 2021, a total of 70 subjects were enrolled in this study, of which 1, 6, 6, 28, and 6 subjects were in the 0.3 mg/kg, 1 mg/kg, 3 mg/kg, 5 mg/kg, and 10 mg/kg groups, respectively. Among all subjects, there were 25 patients with lung cancer, 20 patients with nasopharyngeal cancer, and 2 patients with other tumors.

Table 7. Enrollment in each dose group

| **Tumor type** | **0.3 mg/kg (n)** | **1.0 mg/kg (n)** | **3.0 mg/kg (n)** | **5.0 mg/kg (n)** | **10.0 mg/kg (n)** |
| --- | --- | --- | --- | --- | --- |
| Nasopharyngeal cancer (n = 25) | 0 | 3 | 3 | 16 | 3 |
| Lung cancer (n = 20) | 1 | 3 | 3 | 10 | 3 |
| Other (n = 2) | 0 | 0 | 0 | 2 | 0 |
| Total (n = 47) | 1 | 6 | 6 | 28 | 6 |

###### Safety and tolerability

DLT

As of Mar. 23, 2021, DLT was observed in 2 subjects only in the 10 mg/kg dose group, which are briefly described as follows.

1) The subject with screening number 01030 had nasopharyngeal cancer and received the first dose of QL1706 on Sep. 2, 2020. The subject experienced grade 1 platelet count decreased (related to QL1706) on Sep. 9, 2020, grade 3 platelet count decreased on Sep. 16, and grade 3 platelet count decreased with gingival bleeding (related to QL1706) on Sep. 21, which met the DLT criteria of "grade 3 thrombocytopenia with bleeding".

2) The subject with screening number 01036 had lung cancer and received the first injection of QL1706 at 10 mg/kg on Oct. 14, 2020. On Oct. 29, the biochemical results showed creatinine of 613.9 μmol/L (CTCAE 5.0 grade 4), which was determined to be suspected immune-mediated nephritis (related to QL1706), and was classified as DLT due to meeting the DLT criteria of "grade ≥ 3 non-hematological toxicity". After hospitalization, the blood biochemistry results on Nov. 10 showed creatinine of 124.0 μmol/L. The subject was cured and discharged from hospital on Nov. 20, 2020. QL1706 was permanently discontinued for this subject.

Overview of QL1706-related TEAEs

Among the 70 subjects, 52 (74.3%) subjects experienced QL1706-related TEAEs. In all dose groups, 6 (8.6%) subjects experienced grade ≥ 3 QL1706-related TEAEs, including grade ≥ 3 TEAEs in 3 (50.0%) subjects in the 10 mg/kg dose group and grade ≥ 3 TEAEs in 3 (6.1%) subjects in the 5 mg/kg dose group; 11 (15.7%) subjects experienced grade 2 QL1706-related TEAEs; the other 35 (50.0%) subjects experienced grade 1 QL1706-related TEAEs. The incidences of QL1706-related TEAEs in the 0.3, 1, 3, 5, 7.5, and 10 mg/kg dose groups were 0%, 83.3%, 33.3%, 81.6%, 0%, and 83.3%, respectively.

Among the 70 subjects, QL1706-related TEAEs with an incidence of ≥ 5% included rash (31.4%), pruritus (30.0%), aspartate aminotransferase increased (30.0%), alanine aminotransferase increased (17.1%), blood thyroid stimulating hormone increased (7.1%), fatigue (14.3%), pyrexia (7.1%), hyperthyroidism (14.3%), and hypothyroidism (10.0%).

Among the 70 subjects, grade ≥ 3 QL1706-related TEAEs included aspartate aminotransferase increased (1.4%), platelet count decreased (2.9%), infusion related reactions (1.4%), pneumonitis (1.4%), immune-mediated nephritis (1.4%), cardiac failure chronic (1.4%), and myocarditis (1.4%).

Detailed data are shown in the following table.

Table 8. Summary of investigational drug-related TEAEs by MedDRA SOC and PT and CTCAE (v5.0) grading—SS

|  | **0.3 mg/kg**  **(N = 1)** | **1.0 mg/kg**  **(N = 6)** | | | **3.0 mg/kg**  **(N = 6)** | | | **5.0 mg/kg**  **(N = 49)** | | | | **7.5 mg/kg**  **(N = 2)** | **10 mg/kg**  **(N = 6)** | | | | **Total**  **(N = 70)** | | | |
| --- | --- | --- | --- | --- | --- | --- | --- | --- | --- | --- | --- | --- | --- | --- | --- | --- | --- | --- | --- | --- |
| Classification PT | Total n (%)* | Total n (%)* | Grade 1 n (%)* | Grade 2 n (%)* | Total n (%)* | Grade 1 n (%)* | Grade 2 n (%)* | Total n (%)* | Grade ≥ 3 n (%)* | Grade 1 n (%)* | Grade 2 n (%)* | Total n (%)* | Total n (%)* | Grade ≥ 3 n (%)* | Grade 1 n (%)* | Grade 2 n (%)* | Total n (%)* | Grade ≥ 3 n (%)* | Grade 1 n (%)* | Grade 2 n (%)* |
| TEAE | 0 | 5 (83.3) | 2 (33.3) | 3 (50.0) | 2 (33.3) | 1 (16.7) | 1 (16.7) | 40 (81.6) | 3 (6.1) | 31 (63.3) | 6 (12.2) | 0 | 5 (83.3) | 3 (50.0) | 1 (16.7) | 1 (16.7) | 52 (74.3) | 6 (8.6) | 35 (50.0) | 11 (15.7) |
| Skin and subcutaneous tissue disorders | 0 | 2 (33.3) | 2 (33.3) | 0 | 2 (33.3) | 1 (16.7) | 1 (16.7) | 22 (44.9) | 0 | 21 (42.9) | 1 (2.0) | 0 | 2 (33.3) | 0 | 2 (33.3) | 0 | 28 (40.0) | 0 | 26 (37.1) | 2 (2.9) |
| Rash | 0 | 2 (33.3) | 2 (33.3) | 0 | 2 (33.3) | 1 (16.7) | 1 (16.7) | 17 (34.7) | 0 | 16 (32.7) | 1 (2.0) | 0 | 1 (16.7) | 0 | 1 (16.7) | 0 | 22 (31.4) | 0 | 20 (28.6) | 2 (2.9) |
| Pruritus | 0 | 2 (33.3) | 2 (33.3) | 0 | 2 (33.3) | 1 (16.7) | 1 (16.7) | 15 (30.6) | 0 | 15 (30.6) | 0 | 0 | 2 (33.3) | 0 | 2 (33.3) | 0 | 21 (30.0) | 0 | 20 (28.6) | 1 (1.4) |
| Vitiligo | 0 | 0 | 0 | 0 | 0 | 0 | 0 | 1 (2.0) | 0 | 1 (2.0) | 0 | 0 | 0 | 0 | 0 | 0 | 1 (1.4) | 0 | 1 (1.4) | 0 |
| Hyperhidrosis | 0 | 0 | 0 | 0 | 1 (16.7) | 1 (16.7) | 0 | 0 | 0 | 0 | 0 | 0 | 0 | 0 | 0 | 0 | 1 (1.4) | 0 | 1 (1.4) | 0 |
| Dermatitis | 0 | 0 | 0 | 0 | 0 | 0 | 0 | 1 (2.0) | 0 | 1 (2.0) | 0 | 0 | 0 | 0 | 0 | 0 | 1 (1.4) | 0 | 1 (1.4) | 0 |
| Investigations | 0 | 2 (33.3) | 0 | 2 (33.3) | 1 (16.7) | 0 | 1 (16.7) | 14 (28.6) | 1 (2.0) | 10 (20.4) | 3 (6.1) | 0 | 4 (66.7) | 2 (33.3) | 1 (16.7) | 1 (16.7) | 21 (30.0) | 3 (4.3) | 11 (15.7) | 7 (10.0) |
| Aspartate aminotransferase increased | 0 | 1 (16.7) | 1 (16.7) | 0 | 0 | 0 | 0 | 10 (20.4) | 1 (2.0) | 9 (18.4) | 0 | 0 | 1 (16.7) | 0 | 1 (16.7) | 0 | 12 (17.1) | 1 (1.4) | 11 (15.7) | 0 |
| Alanine aminotransferase increased | 0 | 1 (16.7) | 0 | 1 (16.7) | 0 | 0 | 0 | 4 (8.2) | 0 | 3 (6.1) | 1 (2.0) | 0 | 2 (33.3) | 0 | 2 (33.3) | 0 | 7 (10.0) | 0 | 5 (7.1) | 2 (2.9) |
| Blood thyroid stimulating hormone increased | 0 | 0 | 0 | 0 | 0 | 0 | 0 | 5 (10.2) | 0 | 5 (10.2) | 0 | 0 | 0 | 0 | 0 | 0 | 5 (7.1) | 0 | 5 (7.1) | 0 |
| White blood cell count decreased | 0 | 0 | 0 | 0 | 0 | 0 | 0 | 2 (4.1) | 0 | 2 (4.1) | 0 | 0 | 0 | 0 | 0 | 0 | 2 (2.9) | 0 | 2 (2.9) | 0 |
| Weight decreased | 0 | 1 (16.7) | 0 | 1 (16.7) | 1 (16.7) | 0 | 1 (16.7) | 0 | 0 | 0 | 0 | 0 | 0 | 0 | 0 | 0 | 2 (2.9) | 0 | 0 | 2 (2.9) |
| Platelet count decreased | 0 | 0 | 0 | 0 | 0 | 0 | 0 | 0 | 0 | 0 | 0 | 0 | 2 (33.3) | 2 (33.3) | 0 | 0 | 2 (2.9) | 2 (2.9) | 0 | 0 |
| Neutrophil count decreased | 0 | 0 | 0 | 0 | 0 | 0 | 0 | 2 (4.1) | 0 | 1 (2.0) | 1 (2.0) | 0 | 0 | 0 | 0 | 0 | 2 (2.9) | 0 | 1 (1.4) | 1 (1.4) |
| Bilirubin conjugated increased | 0 | 0 | 0 | 0 | 0 | 0 | 0 | 0 | 0 | 0 | 0 | 0 | 1 (16.7) | 0 | 1 (16.7) | 0 | 1 (1.4) | 0 | 1 (1.4) | 0 |
| Blood thyroid stimulating hormone decreased | 0 | 0 | 0 | 0 | 1 (16.7) | 1 (16.7) | 0 | 0 | 0 | 0 | 0 | 0 | 0 | 0 | 0 | 0 | 1 (1.4) | 0 | 1 (1.4) | 0 |
| Blood bilirubin increased | 0 | 0 | 0 | 0 | 0 | 0 | 0 | 0 | 0 | 0 | 0 | 0 | 1 (16.7) | 0 | 0 | 1 (16.7) | 1 (1.4) | 0 | 0 | 1 (1.4) |
| Thyroxine free increased | 0 | 0 | 0 | 0 | 0 | 0 | 0 | 1 (2.0) | 0 | 1 (2.0) | 0 | 0 | 0 | 0 | 0 | 0 | 1 (1.4) | 0 | 1 (1.4) | 0 |
| Tri-iodothyronine free increased | 0 | 0 | 0 | 0 | 0 | 0 | 0 | 1 (2.0) | 0 | 1 (2.0) | 0 | 0 | 0 | 0 | 0 | 0 | 1 (1.4) | 0 | 1 (1.4) | 0 |
| Lipase increased | 0 | 0 | 0 | 0 | 0 | 0 | 0 | 1 (2.0) | 0 | 0 | 1 (2.0) | 0 | 0 | 0 | 0 | 0 | 1 (1.4) | 0 | 0 | 1 (1.4) |
| Total bile acids increased | 0 | 0 | 0 | 0 | 0 | 0 | 0 | 0 | 0 | 0 | 0 | 0 | 1 (16.7) | 0 | 0 | 1 (16.7) | 1 (1.4) | 0 | 0 | 1 (1.4) |
| General disorders and administration site conditions | 0 | 2 (33.3) | 2 (33.3) | 0 | 0 | 0 | 0 | 11 (22.4) | 0 | 11 (22.4) | 0 | 0 | 3 (50.0) | 0 | 1 (16.7) | 2 (33.3) | 16 (22.9) | 0 | 14 (20.0) | 2 (2.9) |
| Fatigue | 0 | 1 (16.7) | 1 (16.7) | 0 | 0 | 0 | 0 | 8 (16.3) | 0 | 8 (16.3) | 0 | 0 | 1 (16.7) | 0 | 0 | 1 (16.7) | 10 (14.3) | 0 | 9 (12.9) | 1 (1.4) |
| Pyrexia | 0 | 0 | 0 | 0 | 0 | 0 | 0 | 3 (6.1) | 0 | 3 (6.1) | 0 | 0 | 2 (33.3) | 0 | 1 (16.7) | 1 (16.7) | 5 (7.1) | 0 | 4 (5.7) | 1 (1.4) |
| Asthenia | 0 | 1 (16.7) | 1 (16.7) | 0 | 0 | 0 | 0 | 1 (2.0) | 0 | 1 (2.0) | 0 | 0 | 0 | 0 | 0 | 0 | 2 (2.9) | 0 | 2 (2.9) | 0 |
| Swelling face | 0 | 0 | 0 | 0 | 0 | 0 | 0 | 1 (2.0) | 0 | 1 (2.0) | 0 | 0 | 0 | 0 | 0 | 0 | 1 (1.4) | 0 | 1 (1.4) | 0 |
| Edema peripheral | 0 | 1 (16.7) | 1 (16.7) | 0 | 0 | 0 | 0 | 0 | 0 | 0 | 0 | 0 | 0 | 0 | 0 | 0 | 1 (1.4) | 0 | 1 (1.4) | 0 |
| Chest discomfort | 0 | 0 | 0 | 0 | 0 | 0 | 0 | 1 (2.0) | 0 | 1 (2.0) | 0 | 0 | 0 | 0 | 0 | 0 | 1 (1.4) | 0 | 1 (1.4) | 0 |
| Endocrine disorders | 0 | 2 (33.3) | 0 | 2 (33.3) | 0 | 0 | 0 | 7 (14.3) | 0 | 6 (12.2) | 1 (2.0) | 0 | 1 (16.7) | 0 | 0 | 1 (16.7) | 10 (14.3) | 0 | 6 (8.6) | 4 (5.7) |
| Hyperthyroidism | 0 | 1 (16.7) | 0 | 1 (16.7) | 0 | 0 | 0 | 6 (12.2) | 0 | 6 (12.2) | 0 | 0 | 1 (16.7) | 0 | 1 (16.7) | 0 | 8 (11.4) | 0 | 7 (10.0) | 1 (1.4) |
| Hypothyroidism | 0 | 2 (33.3) | 0 | 2 (33.3) | 0 | 0 | 0 | 4 (8.2) | 0 | 3 (6.1) | 1 (2.0) | 0 | 1 (16.7) | 0 | 0 | 1 (16.7) | 7 (10.0) | 0 | 3 (4.3) | 4 (5.7) |
| Gastrointestinal disorders | 0 | 0 | 0 | 0 | 1 (16.7) | 1 (16.7) | 0 | 2 (4.1) | 0 | 2 (4.1) | 0 | 0 | 1 (16.7) | 0 | 1 (16.7) | 0 | 4 (5.7) | 0 | 4 (5.7) | 0 |
| Nausea | 0 | 0 | 0 | 0 | 1 (16.7) | 1 (16.7) | 0 | 1 (2.0) | 0 | 1 (2.0) | 0 | 0 | 0 | 0 | 0 | 0 | 2 (2.9) | 0 | 2 (2.9) | 0 |
| Vomiting | 0 | 0 | 0 | 0 | 1 (16.7) | 1 (16.7) | 0 | 1 (2.0) | 0 | 1 (2.0) | 0 | 0 | 0 | 0 | 0 | 0 | 2 (2.9) | 0 | 2 (2.9) | 0 |
| Diarrhea | 0 | 0 | 0 | 0 | 0 | 0 | 0 | 1 (2.0) | 0 | 1 (2.0) | 0 | 0 | 0 | 0 | 0 | 0 | 1 (1.4) | 0 | 1 (1.4) | 0 |
| Gingival bleeding | 0 | 0 | 0 | 0 | 0 | 0 | 0 | 0 | 0 | 0 | 0 | 0 | 1 (16.7) | 0 | 1 (16.7) | 0 | 1 (1.4) | 0 | 1 (1.4) | 0 |
| Metabolism and nutrition disorders | 0 | 2 (33.3) | 2 (33.3) | 0 | 0 | 0 | 0 | 0 | 0 | 0 | 0 | 0 | 1 (16.7) | 0 | 1 (16.7) | 0 | 3 (4.3) | 0 | 3 (4.3) | 0 |
| Hyponatremia | 0 | 0 | 0 | 0 | 0 | 0 | 0 | 0 | 0 | 0 | 0 | 0 | 1 (16.7) | 0 | 1 (16.7) | 0 | 1 (1.4) | 0 | 1 (1.4) | 0 |
| Hyperglycemia | 0 | 1 (16.7) | 1 (16.7) | 0 | 0 | 0 | 0 | 0 | 0 | 0 | 0 | 0 | 0 | 0 | 0 | 0 | 1 (1.4) | 0 | 1 (1.4) | 0 |
| Decreased appetite | 0 | 1 (16.7) | 1 (16.7) | 0 | 0 | 0 | 0 | 0 | 0 | 0 | 0 | 0 | 0 | 0 | 0 | 0 | 1 (1.4) | 0 | 1 (1.4) | 0 |
| Nervous system disorders | 0 | 0 | 0 | 0 | 0 | 0 | 0 | 3 (6.1) | 0 | 3 (6.1) | 0 | 0 | 0 | 0 | 0 | 0 | 3 (4.3) | 0 | 3 (4.3) | 0 |
| Dizziness | 0 | 0 | 0 | 0 | 0 | 0 | 0 | 2 (4.1) | 0 | 2 (4.1) | 0 | 0 | 0 | 0 | 0 | 0 | 2 (2.9) | 0 | 2 (2.9) | 0 |
| Tremor | 0 | 0 | 0 | 0 | 0 | 0 | 0 | 1 (2.0) | 0 | 1 (2.0) | 0 | 0 | 0 | 0 | 0 | 0 | 1 (1.4) | 0 | 1 (1.4) | 0 |
| Injury, poisoning and procedural complications | 0 | 0 | 0 | 0 | 0 | 0 | 0 | 2 (4.1) | 0 | 1 (2.0) | 1 (2.0) | 0 | 1 (16.7) | 1 (16.7) | 0 | 0 | 3 (4.3) | 1 (1.4) | 1 (1.4) | 1 (1.4) |
| Infusion related reaction | 0 | 0 | 0 | 0 | 0 | 0 | 0 | 2 (4.1) | 0 | 1 (2.0) | 1 (2.0) | 0 | 1 (16.7) | 1 (16.7) | 0 | 0 | 3 (4.3) | 1 (1.4) | 1 (1.4) | 1 (1.4) |
| Infections and infestations | 0 | 0 | 0 | 0 | 0 | 0 | 0 | 2 (4.1) | 0 | 1 (2.0) | 1 (2.0) | 0 | 0 | 0 | 0 | 0 | 2 (2.9) | 0 | 1 (1.4) | 1 (1.4) |
| Conjunctivitis | 0 | 0 | 0 | 0 | 0 | 0 | 0 | 1 (2.0) | 0 | 0 | 1 (2.0) | 0 | 0 | 0 | 0 | 0 | 1 (1.4) | 0 | 0 | 1 (1.4) |
| Herpes virus infection | 0 | 0 | 0 | 0 | 0 | 0 | 0 | 1 (2.0) | 0 | 1 (2.0) | 0 | 0 | 0 | 0 | 0 | 0 | 1 (1.4) | 0 | 1 (1.4) | 0 |
| Ear and labyrinth disorders | 0 | 0 | 0 | 0 | 1 (16.7) | 1 (16.7) | 0 | 0 | 0 | 0 | 0 | 0 | 0 | 0 | 0 | 0 | 1 (1.4) | 0 | 1 (1.4) | 0 |
| Vertigo | 0 | 0 | 0 | 0 | 1 (16.7) | 1 (16.7) | 0 | 0 | 0 | 0 | 0 | 0 | 0 | 0 | 0 | 0 | 1 (1.4) | 0 | 1 (1.4) | 0 |
| Musculoskeletal and connective tissue disorders | 0 | 1 (16.7) | 1 (16.7) | 0 | 0 | 0 | 0 | 0 | 0 | 0 | 0 | 0 | 0 | 0 | 0 | 0 | 1 (1.4) | 0 | 1 (1.4) | 0 |
| Arthralgia | 0 | 1 (16.7) | 1 (16.7) | 0 | 0 | 0 | 0 | 0 | 0 | 0 | 0 | 0 | 0 | 0 | 0 | 0 | 1 (1.4) | 0 | 1 (1.4) | 0 |
| Respiratory, thoracic, and mediastinal disorders | 0 | 0 | 0 | 0 | 0 | 0 | 0 | 1 (2.0) | 1 (2.0) | 0 | 0 | 0 | 0 | 0 | 0 | 0 | 1 (1.4) | 1 (1.4) | 0 | 0 |
| Pneumonitis | 0 | 0 | 0 | 0 | 0 | 0 | 0 | 1 (2.0) | 1 (2.0) | 0 | 0 | 0 | 0 | 0 | 0 | 0 | 1 (1.4) | 1 (1.4) | 0 | 0 |
| Renal and urinary disorders | 0 | 0 | 0 | 0 | 0 | 0 | 0 | 0 | 0 | 0 | 0 | 0 | 1 (16.7) | 1 (16.7) | 0 | 0 | 1 (1.4) | 1 (1.4) | 0 | 0 |
| Immune-mediated nephritis | 0 | 0 | 0 | 0 | 0 | 0 | 0 | 0 | 0 | 0 | 0 | 0 | 1 (16.7) | 1 (16.7) | 0 | 0 | 1 (1.4) | 1 (1.4) | 0 | 0 |
| Cardiac disorders | 0 | 0 | 0 | 0 | 0 | 0 | 0 | 1 (2.0) | 1 (2.0) | 0 | 0 | 0 | 0 | 0 | 0 | 0 | 1 (1.4) | 1 (1.4) | 0 | 0 |
| Cardiac failure chronic | 0 | 0 | 0 | 0 | 0 | 0 | 0 | 1 (2.0) | 1 (2.0) | 0 | 0 | 0 | 0 | 0 | 0 | 0 | 1 (1.4) | 1 (1.4) | 0 | 0 |
| Myocarditis | 0 | 0 | 0 | 0 | 0 | 0 | 0 | 1 (2.0) | 1 (2.0) | 0 | 0 | 0 | 0 | 0 | 0 | 0 | 1 (1.4) | 1 (1.4) | 0 | 0 |

Serious adverse events

Among the 70 subjects, 9 (12.9 %) subjects experienced one SAE each, of which 3 SAEs were not related to QL1706 and 6 SAEs were related to QL1706. Detailed information is tabulated as follows.

Table 9. Listing of SAEs in QL1706-101 study

|  | Total (N = 70) | | | | | |
| --- | --- | --- | --- | --- | --- | --- |
| Classification PT | Total n (%)* | Grade ≥ 3 n (%)* | Grade 1 n (%)* | Grade 2 n (%)* | Grade 3 n (%)* | Grade 4 n (%)* |
| SAE | 9 (12.9) | 7 (10.0) | 1 (1.4) | 1 (1.4) | 3 (4.3) | 4 (5.7) |
| **General disorders and administration site conditions** | 2 (2.9) | 0 | 2 (2.9) | 0 | 0 | 0 |
| Pyrexia | 2 (2.9) | 0 | 2 (2.9) | 0 | 0 | 0 |
| **Infections and infestations** | 1 (1.4) | 1 (1.4) | 0 | 0 | 1 (1.4) | 0 |
| Pneumonia | 1 (1.4) | 1 (1.4) | 0 | 0 | 1 (1.4) | 0 |
| **Investigations** | 1 (1.4) | 1 (1.4) | 0 | 0 | 0 | 1 (1.4) |
| Platelet count decreased | 1 (1.4) | 1 (1.4) | 0 | 0 | 0 | 1 (1.4) |
| **Injury, poisoning and procedural complications** | 1 (1.4) | 1 (1.4) | 0 | 0 | 0 | 1 (1.4) |
| Infusion related reaction | 1 (1.4) | 1 (1.4) | 0 | 0 | 0 | 1 (1.4) |
| **Musculoskeletal and connective tissue disorders** | 1 (1.4) | 0 | 0 | 1 (1.4) | 0 | 0 |
| Neck pain | 1 (1.4) | 0 | 0 | 1 (1.4) | 0 | 0 |
| **Respiratory, thoracic, and mediastinal disorders** | 1 (1.4) | 1 (1.4) | 0 | 0 | 1 (1.4) | 0 |
| Pneumonitis | 1 (1.4) | 1 (1.4) | 0 | 0 | 1 (1.4) | 0 |
| **Neoplasms benign, malignant and unspecified (incl cysts and polyps)** | 1 (1.4) | 1 (1.4) | 0 | 0 | 0 | 1 (1.4) |
| Tumour haemorrhage | 1 (1.4) | 1 (1.4) | 0 | 0 | 0 | 1 (1.4) |
| **Renal and urinary disorders** | 1 (1.4) | 1 (1.4) | 0 | 0 | 0 | 1 (1.4) |
| Immune-mediated nephritis | 1 (1.4) | 1 (1.4) | 0 | 0 | 0 | 1 (1.4) |
| **Cardiac disorders** | 1 (1.4) | 1 (1.4) | 0 | 0 | 1 (1.4) | 0 |
| Myocarditis | 1 (1.4) | 1 (1.4) | 0 | 0 | 1 (1.4) | 0 |

The above data demonstrate that, QL1706 at doses of 0.3–5 mg/kg q3w had good safety and tolerability, and 2 subjects in the 10 mg/kg group experienced DLT.

###### Efficacy

Among the 65 subjects in efficacy evaluable analysis set (EEAS), unconfirmed/confirmed tumor responses were described as follows: overall ORRs were 20.0% (95% CI) (11.102, 31.769)/16.9% (95% CI) (8.763, 28.266), no subject had a best overall response of CR, and 13 (20.0%)/11 (16.9%) subjects had a best overall response of PR; in the 1 mg/kg, 3 mg/kg, 5 mg/kg, and 10 mg/kg dose groups, 2 (33.3%), 1 (16.7%), 8 (16.7%)/6 (12.5%), and 2 (50.0%) subjects had a best overall response of PR, respectively.

Among the 65 subjects in EEAS, 37 subjects had not received any prior immunotherapy, including 17 subjects having received prior first-line therapy and 18 subjects having received prior second-line or later-line therapy, with unconfirmed/confirmed tumor responses as follows: overall ORRs were 29.4% (95% CI) (10.314, 55.958)/29.4% (95% CI) (10.314, 55.958) and 22.2% (95% CI) (6.409, 47.637)/16.7% (95% CI) (3.579, 41.418), respectively; 23 subjects had received prior aPD-1/aPD-L1 therapy, including 8 subjects having received prior first-line therapy and 15 subjects having received prior second-line or later-line therapy, with unconfirmed/confirmed tumor responses as follows: overall ORRs were 0 (95% CI) (0.000, 36.942)/0 (95% CI) (0.000, 36.942) and 20.0% (95% CI) (4.331, 48.089)/13.3% (95% CI) (1.658, 40.460), respectively. The above data suggest a strong correlation between the number of prior treatment lines and efficacy of the investigational drug, i.e., the fewer the number of prior treatment lines, the better the efficacy of the investigational drug may be. The efficacy of the investigational drug not only correlated strongly to the number of prior treatment lines but also correlated to some extent to whether the subjects had received prior immunotherapy. Having received prior aPD-1/aPD-L1 therapy might affect the efficacy of the investigational drug.

Confirmed tumor responses in the 23 subjects with lung cancer in EEAS were described as follows: CR in none of the subjects, PR in 3 subjects, SD in 10 subjects, PD in 10 subjects, overall ORR of 13% (95% CI) (10.314, 55.958), and DCR of 56.5% (95% CI) (34.495, 76.809).

Preliminary efficacies in all the subjects and the subjects with lung cancer based on EEAS are summarized in the following table.

Table 10. Summary of best responses in all the subjects during the trial—EEAS

| Parameter | 0.3 mg/kg (N = 1) n (%) | 1.0 mg/kg (N = 6) n (%) | 3.0 mg/kg (N = 6) n (%) | 5.0 mg/kg (N = 48) n (%) | 7.5 mg/kg (N = 0) n (%) | 10.0 mg/kg (N = 4) n (%) | Total (N = 65) n (%) |
| --- | --- | --- | --- | --- | --- | --- | --- |
| **Summary of unconfirmed best responses—EEAS** | | | | | | | |
| Best overall response during the trial[1] |  |  |  |  |  |  |  |
| CR | 0 | 0 | 0 | 0 | 0 | 0 | 0 |
| PR | 0 | 2 (33.3) | 1 (16.7) | 8 (16.7) | 0 | 2 (50.0) | 13 (20.0) |
| SD | 0 | 1 (16.7) | 2 (33.3) | 16 (33.3) | 0 | 1 (25.0) | 20 (30.8) |
| PD | 1 (100) | 3 (50.0) | 3 (50.0) | 24 (50.0) | 0 | 1 (25.0) | 32 (49.2) |
| NE | 0 | 0 | 0 | 0 | 0 | 0 | 0 |
| Objective response rate[2] |  |  |  |  |  |  |  |
| ORR | 0 | 2 (33.3) | 1 (16.7) | 8 (16.7) | 0 | 2 (50.0) | 13 (20.0) |
| 95% exact CI | (0.000, 97.500) | (4.327, 77.722) | (0.421, 64.123) | (7.481, 30.222) | (-, -) | (6.759, 93.241) | (11.102, 31.769) |
| **Summary of confirmed best responses—EEAS** | | | | | | | |
| Best overall response during the trial[1] |  |  |  |  |  |  |  |
| CR | 0 | 0 | 0 | 0 | 0 | 0 | 0 |
| PR | 0 | 2 (33.3) | 1 (16.7) | 6 (12.5) | 0 | 2 (50.0) | 11 (16.9) |
| SD | 0 | 1 (16.7) | 2 (33.3) | 18 (37.5) | 0 | 1 (25.0) | 22 (33.8) |
| PD | 1 (100) | 3 (50.0) | 3 (50.0) | 24 (50.0) | 0 | 1 (25.0) | 32 (49.2) |
| NE | 0 | 0 | 0 | 0 | 0 | 0 | 0 |
| Objective response rate[2] |  |  |  |  |  |  |  |
| ORR | 0 | 2 (33.3) | 1 (16.7) | 6 (12.5) | 0 | 2 (50.0) | 11 (16.9) |
| 95% exact CI | (0.000, 97.500) | (4.327, 77.722) | (0.421, 64.123) | (4.728, 25.246) | (-, -) | (6.759, 93.241) | (8.763, 28.266) |

[1] CR: complete response; PR: partial response; SD: stable disease; PD: PD; NE: not evaluable.

[2] Objective response rate is the percentage of patients with CR + PR in the efficacy-evaluable patients.

Percentage calculation was based on the number of subjects in each EEAS.

Table 11. Summary of best responses in lung cancer subjects confirmed during the trial—EEAS

| **Parameter** |  |
| --- | --- |
|  | **(N = 23), n (%)** |
| Best overall response during the trial^[1]^ |  |
| CR | 0 |
| PR | 3 (13.0) |
| SD | 10 (43.5) |
| PD | 10 (43.5) |
| NE | 0 |
| Objective response rate^[2]^ |  |
| ORR | 3 (13.0) |
| 95% exact CI | (2.775, 33.589) |
| Disease control rate^[3]^ |  |
| DCR | 13 (56.5) |
| 95% exact CI | (34.495, 76.809) |
| Disease control rate^[4]^ |  |
| DCR | 0 |
| 95% exact CI | (0.000, 14.819) |

[1] CR: complete response; PR: partial response; SD: stable disease; PD: PD; NE: not evaluable.

[2] Objective response rate is the percentage of patients with "CR + PR".

[3] Disease control rate is the percentage of patients with "CR + PR + SD" in the efficacy-evaluable patients.

[4] Disease control rate is the percentage of patients with CR + PR + SD > 3 months in the efficacy-evaluable patients.

###### Pharmacokinetic and pharmacodynamic studies

PK properties of aPD-1 and aCTLA-4 in plasma after intravenous infusion of QL1706 at 0.3–10 mg/kg Q3W are summarized as follows.

Summary of PK properties of aPD-1

PK properties of aCTLA-4 in plasma after intravenous infusion of QL1706 at 0.3–10 mg/kg Q3W are summarized as follows.

- The exposure of aPD-1 in plasma increased with dose;
- After the first dose and after multiple doses (Cycle 6), the median T_max_ of aPD-1 in plasma in each dose group, i.e., 0.52–2.50 h and 0.53–5.57 h, respectively, was similar;
- After the first dose (n = 10) in each dose group, the mean t_1/2_ was 6–9 days, mean CL was 0.0122–0.0159 L/h, and mean Vz was 3.41–3.98 L; after multiple doses (Cycle 6, n = 5), mean CL_ss_ was 0.00676–0.00720 L/h. Based on the data currently available, no data of t_1/2_ and V_ss_ that could be accurately evaluated after multiple doses were obtained;
- After multiple doses (Cycle 6), *in vivo* accumulation of aPD-1 to some extents was observed in each dose group, and mean Rac_C_trough_, mean Rac_C_max_, and mean Rac_AUC were 1.79–2.45 (n = 8), 1.24–1.64 (n = 9), and 1.52–1.84 (n = 4), respectively;
- Between-subject variability in exposure of aPD-1 in plasma was moderate, as after the first dose, the CV% of C_max_, C_trough_, and AUC_0-t_ in each dose group was 12%–21%, 22%–88%, and 17%–27%, respectively; and at Cycle 6, the sample size was relatively small, thus further evaluation will be performed after more data are acquired;
- After multiple consecutive doses, steady state in the 3.0 mg/kg dose group was generally reached at about Cycle 6. In the 1.0 mg/kg and 5.0 mg/kg dose groups, the immediate end-of-infusion concentration and trough concentration of aPD-1 in plasma still tended to increase slightly after Cycle 6 and might have not yet reached the steady state. In the 0.3 mg/kg and 10.0 mg/kg dose groups, less multiple-dose data were acquired, thus steady state status was not yet evaluable. Due to the small sample size currently available and presence of between-subject variability to some extent, further evaluation of steady state has to be performed after more data were available;
- Distributions of dose-normalized exposure parameters of aPD-1 after the first dose in different dose groups were approximately similar, preliminarily indicating that aPD-1 might exhibit linear PK properties *in vivo* after QL1706 was given within the dose range of 0.3–10.0 mg/kg. Due to the small number of subjects receiving multiple doses at present, further evaluation will be performed after more data are acquired;
- In the same dose group, when subjects were tested positive for ADA at baseline, the exposure of aPD-1 slightly decreased, and due to the small number of subjects at present, further evaluation will be performed after more data are acquired;
- The exposure of aPD-1 might correlate with clinical efficacy to some extent.

Summary of PK properties of aCTLA-4

PK properties of aCTLA-4 in plasma after intravenous infusion of QL1706 at 0.3–10 mg/kg Q3W are summarized as follows.

- The exposure of aCTLA-4 in plasma increased with dose;
- After the first dose and after multiple doses (Cycle 6), the T_max_ of aCTLA-4 in plasma in each dose group, i.e., 0.54–2.50 h and 0.52–13.53 h, respectively, was similar;
- After the first dose (n = 31) in each dose group, the mean t_1/2_ was 4–5 days, mean CL was 0.0159–0.0252 L/h, and mean Vz was 2.72–3.92 L. After multiple doses (Cycle 6, n = 8), the mean t_1/2_ was 5–8 days, mean CL_ss_ was 0.0134–0.0225 L/h, and mean V_ss_ was 2.65–3.71 L;
- After multiple doses (Cycle 6), no remarkable *in vivo* accumulation of aCTLA-4 was observed in each dose group, and the mean Rac_C_trough_, mean Rac_C_max_, and mean Rac_AUC were 0.922–1.33 (n = 7), 0.862–1.04 (n = 9), and 0.977–1.05 (n = 8), respectively;
- Between-subject variability in exposure of aCTLA-4 in plasma was moderate and slightly high, as after the first dose, the CV% of C_max_, C_trough_, AUC_0-t_, and AUC_0-inf_ in each dose group was 11%–19%, 43%–136%, 27%–56%, and 26%–51%, respectively; and at Cycle 6, the sample size was relatively small, thus further evaluation will be performed after more data are acquired;
- After multiple consecutive doses, the immediate end-of-infusion concentration and trough concentration of aCTLA-4 in plasma did not increase markedly when compared to those after the first dose, indicating no significant accumulation of aCTLA-4;
- Distributions of dose-normalized exposure parameters of aCTLA-4 after the first dose in different dose groups were similar, preliminarily indicating that aCTLA-4 might exhibit linear PK properties *in vivo* after QL1706 was given within the dose range of 0.3–10.0 mg/kg. Due to the small number of subjects receiving multiple doses at present, further evaluation will be performed after more data are acquired;
- In the same dose group, when subjects were tested positive for ADA at baseline, the exposure of aCTLA-4 slightly decreased, and due to the small number of subjects at present, further evaluation will be performed after more data are acquired;
- The exposure of aCTLA-4 might correlate with clinical efficacy to some extent.

###### PD-1 receptor occupancy study

As of Mar. 23, 2021, the mean PD-1 receptor occupancy in subjects of each dose group exceeded 80% 72 h, 168 h, and 336 h after dosing on C1D1, before dosing and 48 h after dosing on C2D1, and at efficacy evaluation time points (W7, W13, W22, W31, and W40).

The above results showed that the PD-1 receptor occupancy at each time period was comparable in subjects of each dose group after dosing of QL1706 once every 3 weeks.

###### Immunogenicity

The immunogenicity data of subjects as of Mar. 23, 2021 are tabulated as follows.

Table 12. Frequency analysis of ADA test results—immunogenicity analysis set

| Result determination | 0.3 mg/kg  (N = 1)  n (%) | 1.0 mg/kg  (N = 6)  n (%) | 3.0 mg/kg  (N = 6)  n (%) | 5.0 mg/kg  (N = 48)  n (%) | 7.5 mg/kg  (N = 0)  n (%) | 10.0 mg/kg  (N = 4)  n (%) | Total  (N = 65)  n (%) |
| --- | --- | --- | --- | --- | --- | --- | --- |
| **aQL1706** | | | | | | | |
| At least one positive ADA result during the trial | 0 | 4 (66.7) | 4 (66.7) | 19 (39.6) | 0 | 3 (75.0) | 30 (46.2) |
| Not received prior aPD-1 therapy | 0 | 4 (66.7) | 2 (33.3) | 13 (27.1) | 0 | 1 (25.0) | 20 (30.8) |
| Received prior aPD-1 therapy | 0 | 0 | 2 (33.3) | 6 (12.5) | 0 | 2 (50.0) | 10 (15.4) |
| Received prior aPD-1/placebo treatment | 0 | 0 | 0 | 1 (2.1) | 0 | 0 | 1 (1.5) |
| At least one positive post-baseline ADA result | 0 | 3 (50.0) | 4 (66.7) | 19 (39.6) | 0 | 2 (50.0) | 28 (43.1) |
| Negative at baseline | 0 | 3 (50.0) | 4 (66.7) | 12 (25.0) | 0 | 2 (50.0) | 21 (32.3) |
| Positive at baseline | 0 | 0 | 0 | 7 (14.6) | 0 | 0 | 7 (10.8) |
| No change in post-baseline titer^[1]^ | 0 | 0 | 0 | 3 (6.3) | 0 | 0 | 3 (4.6) |
| Increase in post-baseline titer^[2]^ | 0 | 0 | 0 | 4 (8.3) | 0 | 0 | 4 (6.2) |
| Decrease in post-baseline titer^[3]^ | 0 | 0 | 0 | 0 | 0 | 0 | 0 |
| Treatment-induced ADA positivity^[4]^ | 0 | 3 (50.0) | 4 (66.7) | 16 (33.3) | 0 | 2 (50.0) | 25 (38.5) |
| No post-baseline ADA positivity | 1 (100) | 3 (50.0) | 2 (33.3) | 29 (60.4) | 0 | 2 (50.0) | 37 (56.9) |
| Negative at baseline | 1 (100) | 2 (33.3) | 2 (33.3) | 29 (60.4) | 0 | 1 (25.0) | 35 (53.8) |
| Positive at baseline | 0 | 1 (16.7) | 0 | 0 | 0 | 1 (25.0) | 2 (3.1) |
| **aPD-1** | | | | | | | |
| At least one positive ADA result during the trial | 0 | 3 (50.0) | 2 (33.3) | 14 (29.2) | 0 | 2 (50.0) | 21 (32.3) |
| Not received prior aPD-1 therapy | 0 | 3 (50.0) | 1 (16.7) | 10 (20.8) | 0 | 1 (25.0) | 15 (23.1) |
| Received prior aPD-1 therapy | 0 | 0 | 1 (16.7) | 4 (8.3) | 0 | 1 (25.0) | 6 (9.2) |
| Received prior aPD-1/placebo treatment | 0 | 0 | 0 | 1 (2.1) | 0 | 0 | 1 (1.5) |
| At least one positive post-baseline ADA result | 0 | 2 (33.3) | 2 (33.3) | 14 (29.2) | 0 | 2 (50.0) | 20 (30.8) |
| Negative at baseline | 0 | 2 (33.3) | 2 (33.3) | 10 (20.8) | 0 | 2 (50.0) | 16 (24.6) |
| Positive at baseline | 0 | 0 | 0 | 4 (8.3) | 0 | 0 | 4 (6.2) |
| No post-baseline ADA positivity | 1 (100) | 4 (66.7) | 4 (66.7) | 34 (70.8) | 0 | 2 (50.0) | 45 (69.2) |
| Negative at baseline | 1 (100) | 3 (50.0) | 4 (66.7) | 34 (70.8) | 0 | 2 (50.0) | 44 (67.7) |
| Positive at baseline | 0 | 1 (16.7) | 0 | 0 | 0 | 0 | 1 (1.5) |
| **aCTLA-4** | | | | | | | |
| At least one positive ADA result during the trial | 0 | 1 (16.7) | 3 (50.0) | 11 (22.9) | 0 | 3 (75.0) | 18 (27.7) |
| Not received prior aPD-1 therapy | 0 | 1 (16.7) | 1 (16.7) | 7 (14.6) | 0 | 1 (25.0) | 10 (15.4) |
| Received prior aPD-1 therapy | 0 | 0 | 2 (33.3) | 4 (8.3) | 0 | 2 (50.0) | 8 (12.3) |
| Received prior aPD-1/placebo treatment | 0 | 0 | 0 | 0 | 0 | 0 | 0 |
| At least one positive post-baseline ADA result | 0 | 1 (16.7) | 3 (50.0) | 11 (22.9) | 0 | 2 (50.0) | 17 (26.2) |
| Negative at baseline | 0 | 1 (16.7) | 3 (50.0) | 8 (16.7) | 0 | 2 (50.0) | 14 (21.5) |
| Positive at baseline | 0 | 0 | 0 | 3 (6.3) | 0 | 0 | 3 (4.6) |
| No post-baseline ADA positivity | 1 (100) | 5 (83.3) | 3 (50.0) | 37 (77.1) | 0 | 2 (50.0) | 48 (73.8) |
| Negative at baseline | 1 (100) | 5 (83.3) | 3 (50.0) | 37 (77.1) | 0 | 1 (25.0) | 47 (72.3) |
| Positive at baseline | 0 | 0 | 0 | 0 | 0 | 1 (25.0) | 1 (1.5) |

The denominator is the number of subjects in the immunogenicity analysis set of each dose group.

[1] No change in post-baseline titer means that the maximum post-baseline titer is identical to the titer at baseline.

[2] Increase in post-baseline titer means that the maximum post-baseline titer is greater than the titer at baseline.

[3] Decrease in post-baseline titer means that the maximum post-baseline titer is smaller than the titer at baseline.

[4] Treatment-induced ADA positivity was in the population with at least one positive post-baseline ADA result: a) negative at baseline; b) positive at baseline and increased titer after baseline.

The immunogenicity data in the current stage are not so sufficient, making further accumulation necessary before further analysis.

###### Recommended phase II dose

Based on the existing data, the recommended phase II/III dose is 5 mg/kg. The preliminary analysis is detailed as follows.

The interim data of QL1706-101 study (as of Mar. 23, 2021) showed that, in subjects from the 0.3 mg/kg, 1 mg/kg, 3 mg/kg, 5 mg/kg, and 7.5mg/kg q3w dose groups, no DLT was observed while good tolerability and good safety were noted; among a total of 6 subjects included in the 10 mg/kg dose group, DLT, once each, was observed in 2 subjects.

1. Safety and tolerability

- Among the 6 subjects included in the 10 mg/kg dose group, DLT was observed in 2 subjects, while no DLT was reported in other dose groups.
- In the 0.3 mg/kg, 3 mg/kg, and 7.5 mg/kg dose groups, no TEAE-induced dose interruption or permanent discontinuation occurred; in the 0.1 mg/kg group, TEAE-induced dose interruption related to the investigational drug occurred in 1 subject; in the 5 mg/kg dose group, TEAE-induced dose interruption related to the investigational drug occurred in 1 subject, and TEAE-induced permanent discontinuation occurred in 3 subjects, among which such discontinuation in 2 subjects was related to the investigational drug; in the 10 mg/kg dose group, TEAE-induced dose interruption in 1 subject and TEAE-induced permanent discontinuation in 3 subjects occurred, all of which were related to the investigational drug.
- Incidence of QL1706-related TEAEs: The incidences of QL1706-related TEAEs in the 0.3, 1, 3, 5, 7.5, and 10 mg/kg dose groups were 0%, 83.3%, 33.3%, 81.6%, 0%, and 83.3%, respectively.
- The incidences of grade ≥ 3 QL1706-related TEAEs in the 0.3 mg/kg, 1 mg/kg, 3 mg/kg, 5 mg/kg, 7.5 mg/kg, and 10 mg/kg groups were 0, 0, 0, 14.3%, 0, and 50.0%, respectively.

In summary, 5 mg/kg is an acceptable safe dose based on the current data analysis.

1. Preliminary efficacy study results

- The efficacy data of 65 efficacy-evaluable subjects in each dose group are listed as follows.
- CR: No CR was observed in all dose groups for the time being.
- PR: unconfirmed/confirmed: PR in 2 subjects (33.3%)/PR in 2 subjects (33.3%) among 6 efficacy-evaluable subjects in the 1 mg/kg group, PR in 1 subject (16.7%)/PR (16.7%) in 1 subject among 6 efficacy-evaluable subjects in the 3 mg/kg group, PR in 8 subjects (16.7%)/PR in 6 subjects (12.5%) among 48 efficacy-evaluable subjects in the 5 mg/kg group, and PR in 2 subjects (50.0%)/PR in 2 subjects (50.0%) among 4 efficacy-evaluable subjects in the 10 mg/kg group were observed. In the 7.5 mg/kg group, no efficacy-evaluable subject was found for the time being.
- SD: unconfirmed/confirmed: SD in 1 subject (16.7%)/SD in 1 subject (16.7%) among 6 efficacy-evaluable subjects in the 1 mg/kg group, SD in 2 subjects (33.3%)/SD in 2 subjects (33.3%) among 6 efficacy-evaluable subjects in the 3 mg/kg group, SD in 16 subjects (33.3%)/SD in 18 subjects (37.5%) among 48 efficacy-evaluable subjects in the 5 mg/kg group, and SD in 1 subject (25.0%)/SD in 1 subject (25.0%) among 4 efficacy-evaluable subjects in the 10 mg/kg group were observed. In the 7.5 mg/kg group, no efficacy-evaluable subject was found for the time being.
- The efficacy data of 38 efficacy-evaluable subjects with nasopharyngeal cancer are listed as follows.
- CR: No CR was observed in all dose groups for the time being.
- PR: unconfirmed/confirmed: PR in 1 subject (33.3%)/PR in 1 subject (33.3%) among 3 efficacy-evaluable subjects in the 1 mg/kg group, no PR in 3 efficacy-evaluable subjects with nasopharyngeal cancer in the 3 mg/kg group, PR in 8 subjects (25.8%)/PR in 6 subjects (19.4%) among 31 efficacy-evaluable subjects in the 5 mg/kg group, and PR in 1 subject (100%)/PR in 1 subject (100%) among 1 efficacy-evaluable subject with nasopharyngeal cancer in the 10 mg/kg group were observed. In the 7.5 mg/kg group, no efficacy-evaluable subject was found for the time being.
- SD: unconfirmed/confirmed: No SD in 3 efficacy-evaluable subjects with nasopharyngeal cancer in the 1 mg/kg group, SD in 1 subject (33.3%)/SD in 1 subject (33.3%) among 1 efficacy-evaluable subject with nasopharyngeal cancer in the 3 mg/kg group, SD in 7 subjects (22.6%)/SD in 9 subjects (29.0%)among 31 efficacy-evaluable subjects with nasopharyngeal cancer in the 5 mg/kg group, and no SD in 1 efficacy-evaluable subject in the 10 mg/kg group were observed.In the 7.5 mg/kg group, no efficacy-evaluable subject was found for the time being.

Based on the above efficacy data analysis, 5 mg/kg is an acceptable effective dose.

1. Pharmacokinetic properties

In current stage, the data of PK parameters obtained are not yet complete, and full analysis will be performed after complete data are obtained.

The preliminary analysis results of PK parameters after a single dose of QL1706 showed that aPD-1 and aCTLA-4 exposure increased as QL1706 dose increased within the dose range of 0.3–10 mg/kg. Exposure may have a certain correlation with clinical efficacy.

1. Immunogenicity and PD-1 receptor occupancy

By now, the data of immunogenicity and PD-1 receptor occupancy are not yet complete. Current data showed that, no positive post-baseline ADA result was observed in the 0.3 mg/kg group, no remarkable differences among the 1 mg/kg, 3 mg/kg, and 5 mg/kg groups were noted in the incidences of at least one positive post-baseline ADA result and treatment-induced ADA positivity; among 0.3 mg/kg, 1 mg/kg, 3 mg/kg, 5 mg/kg, and 10 mg/kg groups, no remarkable difference in PD-1 receptor occupancy was noted.

In summary, the RP2D is 5 mg/kg q3w based on existing data.

##### QL1706-102 study

QL1706-102 study is a multicenter, open-label, non-randomized phase Ⅰb clinical study of QL1706 in patients with advanced solid malignant tumor in order to evaluate its efficacy and safety in such patients and investigate its immunogenicity and PK properties. It is still ongoing.

The following is a summary of the preliminary efficacy and safety data.

###### Study progress and enrollment

As of Mar. 23, 2021, a total of 208 subjects had been enrolled into this study, including 205 subjects in safety analysis set that consisted of 59 subjects with nasopharyngeal cancer, 58 subjects with lung cancer, 18 subjects with colorectal cancer, 16 subjects with breast cancer, 7 subjects with ovarian cancer, 5 subjects with esophageal cancer, 4 subjects with cervical cancer, 4 subjects with renal cancer, 3 subjects with hepatic cancer, 2 subjects with endometrial cancer, 2 subjects with gastric and gastroesophageal junction adenocarcinoma, 18 subjects with other types of tumors, and 9 subjects with unknown types of tumors (missing value). Among the 59 patients with nasopharyngeal cancer, there were 25 patients with undifferentiated cancer, 13 patients with non-keratinizing differentiated cancer, and 21 patients with other types of nasopharyngeal cancer. The distribution of enrolled subjects in each analysis set is shown in Table 13 below.

Table 13. Patient distribution

| **Analysis datasets** | **5 mg/kg** |
| --- | --- |
|  | **n (%)** |
| Safety analysis set | 205 (98.6) |
| Efficacy-evaluable analysis set | 51 (24.5) |
| Immunogenicity analysis set | 84 (40.4) |

###### Efficacy

Objective response rate (ORR) and disease control rate (DCR)

In the 51 subjects in EEAS, overall ORR was 13.7% (95% CI) (5.701, 26.255), and overall DCR was 56.9% (95% CI) (42.245, 70.655). No subject had a best overall response of CR, 7 (13.7%) subjects had a best overall response of PR, and 22 (43.1%) subjects had a best overall response of SD.

Confirmed tumor responses in the 11 subjects with lung cancer in EEAS were described as follows: CR in none of the subjects, PR in none of the subjects, SD in 8 subjects, PD in 3 subjects, overall ORR of 0% (95% CI) (0.000, 28.491), and DCR of 72.7% (95% CI) (39.026, 93.978).

Preliminary efficacies in all the subjects and the subjects with lung cancer based on EEAS are summarized in the following table.

Table 14. Summary of best responses in all the subjects during the trial—EEAS

| **Parameter** | **5.0 mg/kg** |
| --- | --- |
|  | **(N = 51), n (%)** |
| Best overall response during the trial^[1]^ |  |
| CR | 0 |
| PR | 7 (13.7) |
| SD | 22 (43.1) |
| PD | 22 (43.1) |
| NE | 0 |
| Objective response rate^[2]^ |  |
| ORR | 7 (13.7) |
| 95% exact CI | (5.701, 26.255) |
| Disease control rate^[3]^ |  |
| DCR | 29 (56.9) |
| 95% exact CI | (42.245, 70.655) |
| Disease control rate^[4]^ |  |
| DCR | 0 |
| 95% exact CI | (0.000, 6.978) |

Table 15. Summary of best responses in lung cancer subjects confirmed during the trial—EEAS

| **Parameter** |  |
| --- | --- |
|  | **(N = 11), n (%)** |
| Best overall response during the trial^[1]^ |  |
| CR | 0 |
| PR | 0 |
| SD | 8 (72.7) |
| PD | 3 (27.3) |
| NE | 0 |
| Objective response rate^[2]^ |  |
| ORR | 0 |
| 95% exact CI | (0.000, 28.491) |
| Disease control rate^[3]^ |  |
| DCR | 8 (72.7) |
| 95% exact CI | (39.026, 93.978) |
| Disease control rate^[4]^ |  |
| DCR | 0 |
| 95% exact CI | (0.000, 28.491) |

[1] CR: complete response; PR: partial response; SD: stable disease; PD: PD; NE: not evaluable.

[2] Objective response rate is the percentage of patients with "CR + PR".

[3] Disease control rate is the percentage of patients with "CR + PR + SD" in the efficacy-evaluable patients.

[4] Disease control rate is the percentage of patients with CR + PR + SD > 3 months in the efficacy-evaluable patients.

Relationship between the number of prior treatment lines and efficacy

In efficacy analysis based on EEAS, overall ORR was 13.7% (95% CI) (5.701, 26.255), and overall DCR was 56.9% (95% CI) (42.245, 70.655). No subject had a best overall response of CR, 7 (13.7%) subjects had a best overall response of PR, and 22 (43.1%) subjects had a best overall response of SD. In efficacy analysis based on patients with nasopharyngeal cancer in EEAS, overall ORR was 15.2% (95% CI) (5.109, 31.899), and overall DCR was 54.5% (95% CI) (36.351, 71.893). No subject with nasopharyngeal cancer had a best overall response of CR, 5 (15.2%) subjects with nasopharyngeal cancer had a best overall response of PR, and 13 (39.4%) subjects had a best overall response of SD. Based on data analysis up to now, the overall efficacy in the patients with nasopharyngeal cancer was slightly superior to that in all the subjects.

Among the 51 subjects in EEAS, the subjects having received prior first-line therapy (N = 17) had an overall ORR of 23.5% (95% CI) (6.811, 49.899) and an overall DCR of 58.8% (95% CI) (32.925, 81.556); the subjects having received prior second-line or later-line therapy (N = 33) had an overall ORR of 9.1% (95% CI) (1.915, 24.332) and an overall DCR of 57.6% (95% CI) (39.215, 74.524). Among the 33 subjects with nasopharyngeal cancer in EEAS, the subjects with nasopharyngeal cancer having received prior first-line therapy (N = 10) had an overall ORR of 20.0% (95% CI) (2.521, 55.610) and an overall DCR of 60.0% (95% CI) (26.238, 87.845); the subjects with nasopharyngeal cancer having received prior second-line or later-line therapy (N = 23) had an overall ORR of 13.0% (95% CI) (2.775, 33.589) and an overall DCR of 52.2% (95% CI) (30.588, 73.180). Analysis of the data up to now suggested that, in all subjects and in subjects with nasopharyngeal cancer, the number of prior treatment lines correlated strongly to the efficacy of the investigational drug, and the fewer the number of prior treatment lines, the better the efficacy of the investigational drug may be.

In all the 40 subjects having not received prior immunotherapy, overall ORR was 15%, and overall DCR was 57.5%. In all the 10 subjects having received prior aPD-1/aPD-L1 therapy, overall ORR was 10%, and overall DCR was 50%. In all the 27 subjects with nasopharyngeal cancer having not received prior immunotherapy, overall ORR was 18.5%, and overall DCR was 59.3%. In all the 6 subjects with nasopharyngeal cancer having received prior aPD-1/aPD-L1 therapy, overall ORR was 0%, and overall DCR was 33.3%. Analysis of the data up to now suggested that, in all subjects and in subjects with nasopharyngeal cancer, the efficacy of the investigational drug not only correlated strongly to the number of prior treatment lines but also correlated to some extent to whether the subjects had received prior immunotherapy. Prior aPD-1/aPD-L1 therapy received by patients might affect the efficacy of the investigational drug.

Numbers of prior treatment lines–best responses in all the subjects and the subjects with nasopharyngeal carcinoma based on EEAS are summarized in the following table.

Table 16. Summary of numbers of prior treatment lines–best responses in all the subjects—EEAS

|  | No prior immunotherapy | | Prior aPD-1/aPD-L1 therapy | | Prior aPD-1/Placebo therapy | | Total | |
| --- | --- | --- | --- | --- | --- | --- | --- | --- |
|  | (N = 40) | | (N = 10) | | (N = 0) | | (N = 51) | |
| Parameter | Prior first-line therapy | Prior second-line or later-line therapy | Prior first-line therapy | Prior second-line or later-line therapy | Prior first-line therapy | Prior second-line or later-line therapy | Prior first-line therapy | Prior second-line or later-line therapy |
|  | (N = 14) | (N = 25) | (N = 3) | (N = 7) | (N = 0) | (N = 0) | (N = 17) | (N = 33) |
|  | n (%) | n (%) | n (%) | n (%) | n (%) | n (%) | n (%) | n (%) |
| Best overall response during the trial^[1]^ |  |  |  |  |  |  |  |  |
| CR | 0 | 0 | 0 | 0 | 0 | 0 | 0 | 0 |
| PR | 3 (21.4) | 3 (12.0) | 1 (33.3) | 0 | 0 | 0 | 4 (23.5) | 3 (9.1) |
| SD | 5 (35.7) | 12 (48.0) | 1 (33.3) | 3 (42.9) | 0 | 0 | 6 (35.3) | 16 (48.5) |
| PD | 6 (42.9) | 10 (40.0) | 1 (33.3) | 4 (57.1) | 0 | 0 | 7 (41.2) | 14 (42.4) |
| NE | 0 | 0 | 0 | 0 | 0 | 0 | 0 | 0 |
| Objective response rate^[2]^ |  |  |  |  |  |  |  |  |
| ORR | 3 (21.4) | 3 (12.0) | 1 (33.3) | 0 | 0 | 0 | 4 (23.5) | 3 (9.1) |
| 95% exact CI | (4.658, 50.798) | (2.547, 31.219) | (0.840, 90.570) | (0.000, 40.962) | (-, -) | (-, -) | (6.811, 49.899) | (1.915, 24.332) |
| Disease control rate^[3]^ |  |  |  |  |  |  |  |  |
| DCR | 8 (57.1) | 15 (60.0) | 2 (66.7) | 3 (42.9) | 0 | 0 | 10 (58.8) | 19 (57.6) |
| 95% exact CI | (28.861, 82.339) | (38.665, 78.875) | (9.430, 99.160) | (9.899, 81.595) | (-, -) | (-, -) | (32.925, 81.556) | (39.215, 74.524) |
| Disease control rate^[4]^ |  |  |  |  |  |  |  |  |
| DCR | 0 | 0 | 0 | 0 | 0 | 0 | 0 | 0 |
| 95% exact CI | (0.000, 23.164) | (0.000, 13.719) | (0.000, 70.760) | (0.000, 40.962) | (-, -) | (-, -) | (0.000, 19.506) | (0.000, 10.576) |

The efficacy analysis results of QL1706-102 show that certain efficacy of this investigational drug was observed in solid tumor patients, but since only the first imaging data are available, the efficacy results are for preliminary reference only, and further analysis will be carried out after more data of response evaluation are acquired later.

The latest response evaluation data of QL1706-101&102 study in lung cancer

The response evaluation data of QL1706-101&102 study in lung cancer show that, as of Jun. 30, 2021, there were a total of 118 subjects with lower lung cancer in the 5 mg/kg dose group. Overall ORR was 16.95% (20/118), and DCR was 47.46% (56/118). In the 5 mg/kg dose group, there were 100 efficacy-evaluable subjects with lower lung cancer. Overall ORR was 20% (20/100), and DCR was 56% (56/100). And subgroup analysis shows that the population having not received prior immunotherapy had a higher ORR than the population having received prior immunotherapy.

The phase I study shows that QL1706 had certain anti-tumor efficacy in lung cancer patients, but since it is only a preliminary imaging result analysis, the efficacy results are for preliminary reference only, and further analysis will be carried out after more data of response evaluation are acquired later.

###### Safety

Overview of QL1706-related TEAEs

Among 205 subjects, 53 (25.9%) subjects experienced QL1706-related TEAEs, where QL1706-related TEAEs of grade ≥ 3 occurred in a total of 4 (2.0%) subjects; 11 (5.4%) subjects experienced QL1706-related TEAEs of grade 2; and QL1706-related TEAEs occurring in other 38 (18.5%) subjects were all of grade 1.

In 205 subjects, QL1706-related TEAEs of grade ≥ 3 were increased neutrophil percentage (0.5%), hypomagnesaemia (0.5%), infusion-related reactions (0.5%), and pneumonia (0.5%). And QL1706-related TEAEs of grades 1–2 with an incidence rate ≥ 2% were pruritus, rash, hyperthyroidism, nausea, and dizziness.

Among 205 subjects, 3 (1.5%) patients experienced study-related TEAEs resulting in interruption of the investigational drug, including hepatobiliary disorders (0.5%), pneumonia (0.5%), and dyspnea (0.5%). Study-related TEAEs resulting in permanent discontinuation occurred in 2 (1.0%) patients, including aspartate aminotransferase increased (0.5%) and infusion-related reactions (0.5%).

Detailed data are shown in the following table.

Table 17. Summary of investigational drug-related TEAEs classified by MedDRA SOC and PT and graded by CTCAE (v5.0)—SS

|  | **5.0 mg/kg** | | | | | |
| --- | --- | --- | --- | --- | --- | --- |
|  | **(N = 205)** | | | | | |
| **Class** | **Total** | **Grade ≥ 3** | **Grade 1** | **Grade 2** | **Grade 3** | **Grade 5** |
| **PT** | **n (%)^*^** | **n (%)^*^** | **n (%)^*^** | **n (%)^*^** | **n (%)^*^** | **n (%)^*^** |
| TEAE | 53 (25.9) | 4 (2.0) | 38 (18.5) | 11 (5.4) | 3 (1.5) | 1 (0.5) |
| Investigations | 15 (7.3) | 1 (0.5) | 13 (6.3) | 1 (0.5) | 0 | 1 (0.5) |
| Aspartate aminotransferase increased | 3 (1.5) | 0 | 3 (1.5) | 0 | 0 | 0 |
| Decreased lymphocyte count | 2 (1.0) | 0 | 2 (1.0) | 0 | 0 | 0 |
| Electrocardiogram T wave abnormal | 2 (1.0) | 0 | 2 (1.0) | 0 | 0 | 0 |
| Platelet count decreased | 2 (1.0) | 0 | 1 (0.5) | 1 (0.5) | 0 | 0 |
| White blood cell count decreased | 1 (0.5) | 0 | 1 (0.5) | 0 | 0 | 0 |
| Alanine aminotransferase increased | 1 (0.5) | 0 | 1 (0.5) | 0 | 0 | 0 |
| Amylase increased | 1 (0.5) | 0 | 1 (0.5) | 0 | 0 | 0 |
| Abnormalities in thyroid function test | 1 (0.5) | 0 | 1 (0.5) | 0 | 0 | 0 |
| Positive urine WBC | 1 (0.5) | 0 | 1 (0.5) | 0 | 0 | 0 |
| Blood thyroid stimulating hormone increased | 1 (0.5) | 0 | 1 (0.5) | 0 | 0 | 0 |
| Blood bilirubin increased | 1 (0.5) | 0 | 1 (0.5) | 0 | 0 | 0 |
| Blood iron decreased | 1 (0.5) | 0 | 1 (0.5) | 0 | 0 | 0 |
| Neutrophil percentage increased | 1 (0.5) | 1 (0.5) | 0 | 0 | 0 | 1 (0.5) |
| Neutrophil count decreased | 1 (0.5) | 0 | 1 (0.5) | 0 | 0 | 0 |
| General disorders and administration site conditions | 12 (5.9) | 0 | 9 (4.4) | 3 (1.5) | 0 | 0 |
| Pyrexia | 5 (2.4) | 0 | 3 (1.5) | 2 (1.0) | 0 | 0 |
| Fatigue | 3 (1.5) | 0 | 3 (1.5) | 0 | 0 | 0 |
| Asthenia | 2 (1.0) | 0 | 1 (0.5) | 1 (0.5) | 0 | 0 |
| Chest discomfort | 2 (1.0) | 0 | 2 (1.0) | 0 | 0 | 0 |
| Chills | 1 (0.5) | 0 | 0 | 1 (0.5) | 0 | 0 |
| Skin and subcutaneous tissue disorders | 11 (5.4) | 0 | 9 (4.4) | 2 (1.0) | 0 | 0 |
| Pruritus | 7 (3.4) | 0 | 5 (2.4) | 2 (1.0) | 0 | 0 |
| Rash | 6 (2.9) | 0 | 6 (2.9) | 0 | 0 | 0 |
| Endocrine disorders | 10 (4.9) | 0 | 9 (4.4) | 1 (0.5) | 0 | 0 |
| Hypothyroidism | 4 (2.0) | 0 | 3 (1.5) | 1 (0.5) | 0 | 0 |
| Hyperthyroidism | 4 (2.0) | 0 | 4 (2.0) | 0 | 0 | 0 |
| Secondary hyperthyroidism | 2 (1.0) | 0 | 2 (1.0) | 0 | 0 | 0 |
| Secondary hypothyroidism | 1 (0.5) | 0 | 1 (0.5) | 0 | 0 | 0 |
| Gastrointestinal disorders | 7 (3.4) | 0 | 6 (2.9) | 1 (0.5) | 0 | 0 |
| Nausea | 4 (2.0) | 0 | 4 (2.0) | 0 | 0 | 0 |
| Diarrhea | 1 (0.5) | 0 | 1 (0.5) | 0 | 0 | 0 |
| Vomiting | 1 (0.5) | 0 | 1 (0.5) | 0 | 0 | 0 |
| Dysphagia | 1 (0.5) | 0 | 0 | 1 (0.5) | 0 | 0 |
| Gastrointestinal disorders | 1 (0.5) | 0 | 1 (0.5) | 0 | 0 | 0 |
| Metabolism and nutrition disorders | 6 (2.9) | 1 (0.5) | 4 (2.0) | 1 (0.5) | 1 (0.5) | 0 |
| Decreased appetite | 4 (2.0) | 0 | 3 (1.5) | 1 (0.5) | 0 | 0 |
| Hypoproteinemia | 1 (0.5) | 0 | 0 | 1 (0.5) | 0 | 0 |
| Hypophosphatemia | 1 (0.5) | 0 | 1 (0.5) | 0 | 0 | 0 |
| Hypomagnesemia | 1 (0.5) | 1 (0.5) | 0 | 0 | 1 (0.5) | 0 |
| Hyperuricemia | 1 (0.5) | 0 | 1 (0.5) | 0 | 0 | 0 |
| Injury, poisoning and procedural complications | 5 (2.4) | 1 (0.5) | 3 (1.5) | 1 (0.5) | 1 (0.5) | 0 |
| Infusion related reaction | 5 (2.4) | 1 (0.5) | 3 (1.5) | 1 (0.5) | 1 (0.5) | 0 |
| Nervous system disorders | 4 (2.0) | 0 | 3 (1.5) | 1 (0.5) | 0 | 0 |
| Dizziness | 4 (2.0) | 0 | 4 (2.0) | 0 | 0 | 0 |
| Headache | 1 (0.5) | 0 | 0 | 1 (0.5) | 0 | 0 |
| Respiratory, thoracic, and mediastinal disorders | 4 (2.0) | 0 | 1 (0.5) | 3 (1.5) | 0 | 0 |
| Cough | 2 (1.0) | 0 | 0 | 2 (1.0) | 0 | 0 |
| Hemoptysis | 1 (0.5) | 0 | 1 (0.5) | 0 | 0 | 0 |
| Tachypnea | 1 (0.5) | 0 | 1 (0.5) | 0 | 0 | 0 |
| Dyspnea | 1 (0.5) | 0 | 0 | 1 (0.5) | 0 | 0 |
| Exertional dyspnea | 1 (0.5) | 0 | 0 | 1 (0.5) | 0 | 0 |
| Blood and lymphatic system disorders | 4 (2.0) | 1 (0.5) | 1 (0.5) | 2 (1.0) | 1 (0.5) | 0 |
| Anemia | 2 (1.0) | 1 (0.5) | 1 (0.5) | 0 | 1 (0.5) | 0 |
| Lymphadenopathy | 1 (0.5) | 0 | 0 | 1 (0.5) | 0 | 0 |
| Iron-deficiency anemia | 1 (0.5) | 0 | 0 | 1 (0.5) | 0 | 0 |
| Hepatobiliary disorders | 3 (1.5) | 0 | 1 (0.5) | 2 (1.0) | 0 | 0 |
| Abnormal hepatic function | 3 (1.5) | 0 | 1 (0.5) | 2 (1.0) | 0 | 0 |
| Renal and urinary disorders | 2 (1.0) | 0 | 1 (0.5) | 1 (0.5) | 0 | 0 |
| Renal impairment | 1 (0.5) | 0 | 1 (0.5) | 0 | 0 | 0 |
| Hematuria | 1 (0.5) | 0 | 0 | 1 (0.5) | 0 | 0 |
| Cardiac disorders | 2 (1.0) | 0 | 2 (1.0) | 0 | 0 | 0 |
| Sinus bradycardia | 2 (1.0) | 0 | 2 (1.0) | 0 | 0 | 0 |
| Infections and infestations | 1 (0.5) | 1 (0.5) | 0 | 0 | 1 (0.5) | 0 |
| Pneumonia | 1 (0.5) | 1 (0.5) | 0 | 0 | 1 (0.5) | 0 |
| Herpes virus infection | 1 (0.5) | 0 | 1 (0.5) | 0 | 0 | 0 |
| Musculoskeletal and connective tissue disorders | 1 (0.5) | 0 | 1 (0.5) | 0 | 0 | 0 |
| Myasthenia | 1 (0.5) | 0 | 1 (0.5) | 0 | 0 | 0 |
| Immune system disorders | 1 (0.5) | 0 | 1 (0.5) | 0 | 0 | 0 |
| Immunodeficiency | 1 (0.5) | 0 | 1 (0.5) | 0 | 0 | 0 |
| Uncoded | 9 (4.4) | 0 | 5 (2.4) | 4 (2.0) | 0 | 0 |
| Uncoded | 9 (4.4) | 0 | 5 (2.4) | 4 (2.0) | 0 | 0 |

Table 18. Summary of study-related TEAEs resulting in interruption of the investigational drug classified by MedDRA SOC and PT and graded by CTCAE (v5.0)—SS

|  | **5.0 mg/kg** | | | |
| --- | --- | --- | --- | --- |
|  | **(N = 205)** | | | |
| **Class** | **Total** | **Grade ≥ 3** | **Grade 2** | **Grade 3** |
| **PT** | **n (%)^*^** | **n (%)^*^** | **n (%)^*^** | **n (%)^*^** |
| TEAE | 3 (1.5) | 1 (0.5) | 2 (1.0) | 1 (0.5) |
| Hepatobiliary disorders | 1 (0.5) | 0 | 1 (0.5) | 0 |
| Infections and infestations | 1 (0.5) | 1 (0.5) | 0 | 1 (0.5) |
| Pneumonia | 1 (0.5) | 1 (0.5) | 0 | 1 (0.5) |
| Respiratory, thoracic, and mediastinal disorders | 1 (0.5) | 0 | 1 (0.5) | 0 |
| Dyspnea | 1 (0.5) | 0 | 1 (0.5) | 0 |

Table 19. Summary of study-related TEAEs resulting in permanent discontinuation classified by MedDRA SOC and PT and graded by CTCAE (v5.0)—SS

|  | **5.0 mg/kg** | | | |
| --- | --- | --- | --- | --- |
|  | **(N = 205)** | | | |
| **Class** | **Total** | **Grade ≥ 3** | **Grade 1** | **Grade 3** |
| **PT** | **n (%)^*^** | **n (%)^*^** | **n (%)^*^** | **n (%)^*^** |
| TEAE | 2 (1.0) | 1 (0.5) | 1 (0.5) | 1 (0.5) |
| Investigations | 1 (0.5) | 0 | 1 (0.5) | 0 |
| Aspartate aminotransferase increased | 1 (0.5) | 0 | 1 (0.5) | 0 |
| Injury, poisoning and procedural complications | 1 (0.5) | 1 (0.5) | 0 | 1 (0.5) |
| Infusion related reaction | 1 (0.5) | 1 (0.5) | 0 | 1 (0.5) |

Immune-related adverse events

Immune-related TEAEs occurred in a total of 23 out of 205 subjects, including immune-related TEAEs of grade 3 in 3 subjects, which consist of infusion-related reactions of grade 3, anemia of grade 3, and pneumonia of grade 3; while the remaining immune-related TEAEs were all of grade 1 or 2.

Detailed data are shown in the following table.

Table 20. Summary of immune-related AEs classified by MedDRA SOC and PT and graded by CTCAE (v5.0)—SS

|  | **5.0 mg/kg** | | | | |
| --- | --- | --- | --- | --- | --- |
|  | **(N = 205)** | | | | |
| **Class** | **Total** | **Grade ≥ 3** | **Grade 1** | **Grade 2** | **Grade 3** |
| **PT** | **n (%)^*^** | **n (%)^*^** | **n (%)^*^** | **n (%)^*^** | **n (%)^*^** |
| Immune-related adverse events | 23 (11.2) | 3 (1.5) | 14 (6.8) | 6 (2.9) | 3 (1.5) |
| Endocrine disorders | 8 (3.9) | 0 | 7 (3.4) | 1 (0.5) | 0 |
| Hyperthyroidism | 4 (2.0) | 0 | 4 (2.0) | 0 | 0 |
| Secondary hyperthyroidism | 2 (1.0) | 0 | 2 (1.0) | 0 | 0 |
| Hypothyroidism | 2 (1.0) | 0 | 1 (0.5) | 1 (0.5) | 0 |
| Secondary hypothyroidism | 1 (0.5) | 0 | 1 (0.5) | 0 | 0 |
| Skin and subcutaneous tissue disorders | 7 (3.4) | 0 | 5 (2.4) | 2 (1.0) | 0 |
| Pruritus | 5 (2.4) | 0 | 3 (1.5) | 2 (1.0) | 0 |
| Rash | 3 (1.5) | 0 | 3 (1.5) | 0 | 0 |
| Investigations | 5 (2.4) | 0 | 4 (2.0) | 1 (0.5) | 0 |
| Amylase increased | 1 (0.5) | 0 | 1 (0.5) | 0 | 0 |
| Positive urine WBC | 1 (0.5) | 0 | 1 (0.5) | 0 | 0 |
| Aspartate aminotransferase increased | 1 (0.5) | 0 | 1 (0.5) | 0 | 0 |
| Electrocardiogram T wave abnormal | 1 (0.5) | 0 | 1 (0.5) | 0 | 0 |
| Blood iron decreased | 1 (0.5) | 0 | 1 (0.5) | 0 | 0 |
| Platelet count decreased | 1 (0.5) | 0 | 0 | 1 (0.5) | 0 |
| Hepatobiliary disorders | 3 (1.5) | 0 | 1 (0.5) | 2 (1.0) | 0 |
| Abnormal hepatic function | 3 (1.5) | 0 | 1 (0.5) | 2 (1.0) | 0 |
| Injury, poisoning and procedural complications | 3 (1.5) | 1 (0.5) | 1 (0.5) | 1 (0.5) | 1 (0.5) |
| Infusion related reaction | 3 (1.5) | 1 (0.5) | 1 (0.5) | 1 (0.5) | 1 (0.5) |
| Blood and lymphatic system disorders | 3 (1.5) | 1 (0.5) | 0 | 2 (1.0) | 1 (0.5) |
| Lymphadenopathy | 1 (0.5) | 0 | 0 | 1 (0.5) | 0 |
| Anemia | 1 (0.5) | 1 (0.5) | 0 | 0 | 1 (0.5) |
| Iron-deficiency anemia | 1 (0.5) | 0 | 0 | 1 (0.5) | 0 |
| Gastrointestinal disorders | 2 (1.0) | 0 | 1 (0.5) | 1 (0.5) | 0 |
| Nausea | 1 (0.5) | 0 | 1 (0.5) | 0 | 0 |
| Dysphagia | 1 (0.5) | 0 | 0 | 1 (0.5) | 0 |
| Metabolism and nutrition disorders | 1 (0.5) | 0 | 1 (0.5) | 0 | 0 |
| Hyperuricemia | 1 (0.5) | 0 | 1 (0.5) | 0 | 0 |
| Infections and infestations | 1 (0.5) | 1 (0.5) | 0 | 0 | 1 (0.5) |
| Pneumonia | 1 (0.5) | 1 (0.5) | 0 | 0 | 1 (0.5) |
| Immune system disorders | 1 (0.5) | 0 | 1 (0.5) | 0 | 0 |
| Immunodeficiency | 1 (0.5) | 0 | 1 (0.5) | 0 | 0 |
| General disorders and administration site conditions | 1 (0.5) | 0 | 1 (0.5) | 0 | 0 |
| Pyrexia | 1 (0.5) | 0 | 1 (0.5) | 0 | 0 |
| Renal and urinary disorders | 1 (0.5) | 0 | 1 (0.5) | 0 | 0 |
| Renal impairment | 1 (0.5) | 0 | 1 (0.5) | 0 | 0 |

Serious adverse events

Among 205 subjects, 7 (3.4 %) subjects experienced a total of 8 SAEs, of which SAEs in 3 subjects were not related to QL1706 and SAEs in 4 subjects were QL1706-related. Among which 1 subject experienced 2 SAEs, consisting of 1 SAE not related to QL1706 and 1 SAE related.

Detailed information is tabulated as follows.

Table 21. Summary of SAEs classified by MedDRA SOC and PT—SS

|  | **5.0 mg/kg** | | | | |
| --- | --- | --- | --- | --- | --- |
|  | **(N = 205)** | | | | |
| **Class** | **Total** | **Grade ≥ 3** | **Grade 2** | **Grade 3** | **Grade 4** |
| **PT** | **n (%)^*^** | **n (%)^*^** | **n (%)^*^** | **n (%)^*^** | **n (%)^*^** |
| SAE | 7 (3.4) | 4 (2.0) | 3 (1.5) | 3 (1.5) | 1 (0.5) |
| Gastrointestinal disorders | 2 (1.0) | 2 (1.0) | 0 | 1 (0.5) | 1 (0.5) |
| Ascites | 1 (0.5) | 1 (0.5) | 0 | 1 (0.5) | 0 |
| Upper digestive tract hemorrhage | 1 (0.5) | 1 (0.5) | 0 | 0 | 1 (0.5) |
| Hepatobiliary disorders | 1 (0.5) | 0 | 1 (0.5) | 0 | 0 |
| Abnormal hepatic function | 1 (0.5) | 0 | 1 (0.5) | 0 | 0 |
| Infections and infestations | 1 (0.5) | 1 (0.5) | 0 | 1 (0.5) | 0 |
| Pneumonia | 1 (0.5) | 1 (0.5) | 0 | 1 (0.5) | 0 |
| Nervous system disorders | 1 (0.5) | 1 (0.5) | 0 | 1 (0.5) | 0 |
| Headache | 1 (0.5) | 1 (0.5) | 0 | 1 (0.5) | 0 |
| Respiratory, thoracic, and mediastinal disorders | 1 (0.5) | 0 | 1 (0.5) | 0 | 0 |
| Dyspnea | 1 (0.5) | 0 | 1 (0.5) | 0 | 0 |
| General disorders and administration site conditions | 1 (0.5) | 0 | 1 (0.5) | 0 | 0 |
| Pyrexia | 1 (0.5) | 0 | 1 (0.5) | 0 | 0 |
| Renal and urinary disorders | 1 (0.5) | 0 | 1 (0.5) | 0 | 0 |
| Hematuria | 1 (0.5) | 0 | 1 (0.5) | 0 | 0 |

The above data show that QL1706, at a dose of 5 mg/kg once every 3 weeks, had good tolerability and safety.

###### PD-1 receptor occupancy study

As of Mar. 23, 2021, PD-1 receptor occupancy data were obtained from 26 subjects. Currently, only the data at 168 h and 336 h after dosing on C1D1 are obtained due to the short time of dosing in the subjects, and as shown by the data, values of mean PD-1 receptor occupancy are all above 90%. Detailed data are shown in the following table.

Table 22. Summary of PD-1 receptor occupancy results–SS

| **Visit** | **Total** |
| --- | --- |
| **Class** | **(N = 205), n (%)** |
| Baseline |  |
| Number of subjects (missing value) | 26 (179) |
| Mean (standard deviation) | 14.44 (36.227) |
| Median | -2.2 |
| Minimum - Maximum | -6 - 108.7 |
| C1 - 168 h after start of dosing |  |
| Number of subjects (missing value) | 12 (193) |
| Mean (standard deviation) | 99.45 (3.103) |
| Median | 99.05 |
| Minimum - Maximum | 94.8 - 105.4 |
| Change from baseline |  |
| Number of subjects (missing value) | 12 (193) |
| Mean (standard deviation) | 78.53 (43.977) |
| Median | 102 |
| Minimum - Maximum | -11.4 - 106.9 |
| C1 - 336 h after start of dosing |  |
| Number of subjects (missing value) | 2 (203) |
| Mean (standard deviation) | 102.80 (5.233) |
| Median | 102.8 |
| Minimum - Maximum | 99.1 - 106.5 |
| Change from baseline |  |
| Number of subjects (missing value) | 2 (203) |
| Mean (standard deviation) | 53.55 (67.670) |
| Median | 53.55 |
| Minimum - Maximum | 5.7 - 101.4 |

The above data suggest that QL1706 maintains adequate PD-1 receptor occupancy when administered at a frequency of once every 3 weeks.

###### Immunogenicity

Based on the results of QL1706 composition-induced ADA assay, at least 1 positive result of ADA was observed in 27 out of 84 subjects, among which 6 subjects received prior anti-PD-1 monoclonal antibody.

Detailed values are shown in the following table.

Table 23. Frequency analysis of QL1706-induced ADA test results—IAS

| **Result determination** | **Total** |
| --- | --- |
|  | **(N = 84), n (%)** |
| At least one positive ADA result during the trial | 27 (32.1) |
| Not received prior aPD-1 therapy | 21 (25.0) |
| Received prior aPD-1 therapy | 6 (7.1) |
| Received prior aPD-1/placebo treatment | 0 |
| At least one positive post-baseline ADA result | 27 (32.1) |
| Negative at baseline | 24 (28.6) |
| Positive at baseline | 3 (3.6) |
| No change in post-baseline titer^[1]^ | 1 (1.2) |
| Increase in post-baseline titer^[2]^ | 1 (1.2) |
| Decrease in post-baseline titer^[3]^ | 1 (1.2) |
| Treatment-induced ADA positivity^[4]^ | 25 (29.8) |
| No post-baseline ADA positivity | 57 (67.9) |
| Negative at baseline | 57 (67.9) |
| Positive at baseline | 0 |

[1] No change in post-baseline titer means that the maximum post-baseline titer is identical to the titer at baseline.
[2] Increase in post-baseline titer means that the maximum post-baseline titer is greater than the titer at baseline.
[3] Decrease in post-baseline titer means that the maximum post-baseline titer is smaller than the titer at baseline.
[4] Treatment-induced ADA-positivity means that in the population with at least one post-baseline ADA-positive result: a) negative result at baseline; b) positive result at baseline but post-baseline titer increased.

Immunogenicity-efficacy analysis

The best tumor response analysis results of 84 subjects in IAS show that, out of 27 subjects having at least one positive post-baseline ADA result, 4 subjects achieved PR and 7 subjects achieved SD; out of 25 subjects having treatment-induced positive ADA results, 3 subjects achieved PR and 6 subjects SD; out of 57 subjects having no positive post-baseline ADA result, 3 subjects PR and 15 subjects SD. A summary of the data is shown in the following table.

Table 24. Summary of best tumor responses based on ADA status—IAS

| **Parameter** | **At least one positive post-baseline ADA result** | **Treatment-induced ADA-positivity** | **No post-baseline ADA positivity** |
| --- | --- | --- | --- |
|  | **(N = 27)** | **(N = 25)** | **(N = 57)** |
| Best overall response during the trial |  |  |  |
| CR | 0 | 0 | 0 |
| PR | 4 (14.8) | 3 (12.0) | 3 (5.3) |
| SD | 7 (25.9) | 6 (24.0) | 15 (26.3) |
| PD | 5 (18.5) | 5 (20.0) | 17 (29.8) |
| NE | 0 | 0 | 0 |

Immunogenicity - safety analysis

Among 84 subjects included in IAS, 27 subjects had at least one positive post-baseline ADA result, 25 subjects had treatment-induced positive ADA results, and 57 subjects did not have any positive post-baseline ADA result.

Among 27 subjects having at least one positive post-baseline ADA result, 14 subjects experienced QL1706-related TEAEs, consisting of grade 1 in 8 subjects, grade 4 in 5 subjects, and grade ≥ 3 in 2 subjects.

Among 25 subjects having treatment-induced ADA-positivity, 12 subjects experienced QL1706-related TEAEs, consisting of grade 1 in 6 subjects, grade 2 in 4 subjects, and grade ≥ 3 in 2 subjects.

Among 57 subjects having no positive post-baseline ADA result, 29 subjects experienced QL1706-related TEAEs, consisting of grade 1 in 24 subjects, grade 2 in 4 subjects, and grade ≥ 3 in 1 subject.

The data show that no significant differences in number and severity of QL1706-related TEAEs were observed between subjects with at least one positive post-baseline ADA result, subjects with treatment-induced ADA-positivity, and subjects with no positive post-baseline ADA result.

##### Summary of safety findings of phase I clinical study of QL1706

As of Mar. 23, 2021, a total of 278 subjects were enrolled in the two phase I clinical studies of QL1706 in China, including 275 subjects in the SS. The following summary of safety data is based on SS.

- QL1706 at doses of 0.3–5.0 mg/kg q3w had good safety and tolerability, and the 7.5 mg/kg dose group is under safety and tolerability observation; 2 subjects in the 10 mg/kg group experienced DLT (grade 3).
- In the 10 mg/kg dose group, DLT was observed in 2 subjects: (1) a patient with nasopharyngeal carcinoma, experiencing platelet count decreased of grade 3 associated with gum bleeding (QL1706-related) meeting the criterion for DLT "thrombocytopenia of grade 3 associated with hemorrhage symptom" 14 days after the first dose; and (2) a patient with lung cancer, experiencing suspected immune nephritis (QL1706-related) meeting the criterion for DLT "non-hematological toxicity of grade 3 or greater" after the first dose.
- In 275 subjects, no TEAE resulting in death occurred.
- SAEs occurred in 16 (5.8%) out of 275 subjects, in which the SAEs in 10 (3.6%) subjects were QL1706-related.
- Immune-related AEs occurred in 60 (21.8%) out of 275 subjects, in which AEs in 59 subjects were QL1706-related.
- QL1706-related TEAEs occurred in 148 (53.8 %) out of 275 subjects, in which TEAEs in 105 (38.2%) subjects were QL1706-related. There were QL1706-related TEAEs of grade ≥ 3 in 10 (3.6%) subjects, TEAEs of grade 2 in 22 (8.0%) subjects, and grade 1 in 73 (26.5%) subjects.

QL1706-related TEAEs with an incidence rate ≥ 5% in 275 subjects consist of rash in 28 (10.2%) subjects and pruritus in 28 (10.2%) subjects.

## Potential Risks and Benefits

Regarding safety, the results of completed preclinical studies show that dosing of QL1706 did not affect parameters of clinical vital signs, food consumption, body weight, ophthalmology, ECG, coagulation, and urinalysis. QL1706-related changes in hematological parameters are restricted to mild decrease in lymphocyte count only in male and female animals from the 60 mg/kg dose group on Day 3. Changes in clinical chemistry parameters include slight decreases in albumin and cholesterol and slight increase in globulin in female animals from the 60 mg/kg dose group on Day 31. Changes in albumin and globulin resulted in a decrease in albumin:globulin ratio. Such changes indicate mild acute-phase reaction, which is probably related to KLH-stimulated local reaction and irrelevant to dosing of QL1706. Additionally, QL1706 and its components enhanced activation of T-cells in human Allo-MLR and SEB-induced T-cell activation, and showed cumulative antitumor effect in a number of tumor models.

In general, there is a very great unmet need for treatment of patients with advanced malignancies, while the results of phase I clinical study of QL1706 have shown good safety, suggesting that the safety of QL1706 is controllable in clinical medication. In the field of first-line treatment of non-small cell lung cancer (NSCLC), multiple anti-PD-1 antibody treatment options have been marketed in China, including anti-PD-1 antibody monotherapy (for PD-L1-positive patients) or anti-PD-1 antibody combined with chemotherapy (without limitation in PD-L1 status of the patient), combination with adequate chemotherapy is still needed for first-line treatment in NSCLC population without PD-L1 expression restrictions, where unmet clinical need is present. In May of 2020, based on CheckMate-9LA study, FDA approved nivolumab + ipilimumab + 2 cycles of chemotherapy for first-line treatment of advanced NSCLC without EGFR/ALK mutation, including subjects with squamous cell carcinoma and those with non-squamous cell carcinoma, and also without PD-L1 expression restrictions. QL1706 has the same target as Nivolumab combined with Ipilimumab, QL1706 combined with chemotherapy for first-line treatment in NSCLC population has good scientific rationale, and Cohort 1 and Cohort 2 for this study were designed with reference to the study groups in CheckMate-9LA study. For patients with EGFR-positive non-squamous NSCLC failing EGFR-TKI therapy, no excellent treatment option is available now. IMPower150 study suggests that, compared with simple chemotherapy + bevacizumab group, atezolizumab combined with chemotherapy + bevacizumab had better survival benefit. QL1706 is an immunosuppressive preparation bispecific to PD-1 and CTLA-4, and a Cohort 3 was added in this study to the first-line treatment of NSCLC pursuant to design of IMPower150 study, in order to observe the safety and efficacy of QL1706 combined with chemotherapy ± bevacizumab in patients with EGFR-positive advanced non-squamous cell carcinoma failing TKI therapy.

# STUDY OBJECTIVES AND ENDPOINTS

## Study Objectives

### Primary objective

To evaluate the safety of QL1706 combined with chemotherapy ± bevacizumab in patients with advanced NSCLC.

### Secondary objectives

1. To evaluate the efficacy of QL1706 combined with chemotherapy ± bevacizumab in patients with NSCLC.
2. To evaluate the pharmacokinetic (PK) characteristics of QL1706 combined with chemotherapy ± bevacizumab in patients with NSCLC.
3. To evaluate the immunogenicity of QL1706 combined with chemotherapy ± bevacizumab in patients with NSCLC.

### Exploratory objectives

1. To explore the efficacy as per iRECIST.
2. To explore the correlation between certain biomarkers and the anti-tumor activity of QL1706.
3. To explore the correlation between QL1706 exposure and clinical efficacy, safety, and immunogenicity in patients with advanced NSCLC.

## Study Endpoints

### Primary endpoint

Safety endpoints: AE, TEAE occurrence, vital signs, and laboratory test parameters.

### Secondary endpoints

1. Objective response rate (ORR, per RECIST v1.1).
2. Progression-free survival (PFS, per RECIST v1.1).
3. Duration of response (DOR, per RECIST v1.1).
4. Disease control rate (DCR, per RECIST v1.1).
5. Overall survival (OS).
6. 1-year OS.
7. The PK characteristics of QL1706 combined with chemotherapy ± bevacizumab.
8. The immunogenicity of QL1706 combined with chemotherapy ± bevacizumab.

### Exploratory endpoints

1. The ORR/PFS/DOR/DCR per iRECIST.
2. To explore whether PD-L1 and TMB are predictive biomarkers of clinical efficacy.
3. The correlation between QL1706 exposure and clinical efficacy, safety, and immunogenicity in patients with advanced NSCLC.

# STUDY DESIGN

This is a single-arm, non-randomized, open-label, multi-center phase II clinical study to investigate the safety and efficacy of QL1706 combined with chemotherapy ± bevacizumab in first-line treatment of patients with advanced NSCLC.

This study consists of two cohorts: Cohort 1: advanced squamous NSCLC, dosing: QL1706 + paclitaxel + carboplatin; Cohort 2: advanced non-squamous NSCLC, dosing: QL1706 + pemetrexed + carboplatin ± bevacizumab.

The study plans to enroll 20 subjects to Cohort 1 and 40 subjects to Cohort 2. Twenty subjects are enrolled directly to Cohort 1; In Cohort 2, 3 subjects are first enrolled to receive QL1706 + pemetrexed + carboplatin and are subject to safety observation from the start of dosing to the end of Cycle 1 (21 days after the first dose). Adverse events (AEs) are evaluated by investigators as per NCI-CTCAE 5.0. When all first enrolled subjects completed Cycle 1 treatment, safety and tolerability can be confirmed if the following drug-related AEs are reported among < 1/3 of the subjects only. Thereafter, 3 more subjects can be enrolled to receive QL1706 + bevacizumab + pemetrexed + carboplatin and are subject to safety observation for 4-drug combination regimen. Similarly, safety and tolerability can be confirmed if the following drug-related AEs are reported among < 1/3 of the subjects only. Following discussion and confirmation by investigator and sponsor, more subjects can be enrolled to receive the combination of QL1706 + bevacizumab + pemetrexed + carboplatin, until 40 subjects are enrolled to Cohort 2, including at least 20 EGFR-positive subjects. If drug-related AEs are reported in ≥ 1/3 of the subjects, three other subjects will be enrolled for further evaluation of safety and tolerability, or dose adjustment or study termination will be considered for safety following discussion between investigator and sponsor. During the study, the number of subjects enrolled in each cohort will be promptly adjusted according to the efficacy and safety data of each treatment regimen.

Tolerability of combination therapy will be evaluated based on the occurrence of the following drug-related AEs:

∙ Non-hematological adverse reaction of grade ≥ 3 (excluding transient electrolyte abnormality, diarrhea, nausea, and vomiting that could resolve to grade 2 or below within 3 days after best supportive care, and fatigue that could resolve to grade 2 or below within 7 days after best supportive care), and cardiac insufficiency of grade 2 or above;

∙ Thrombocytopenia of grade 4 or thrombocytopenia of grade 3 associated with remarkable clinical bleeding tendency, neutropenia of grade 4 persisting for a duration ≥ 7 days or neutropenia of grade 3 associated with fever at a temperature ≥ 38.3 °C;

∙ Other non-hematological toxicity of grade 4;

∙ Other circumstances of subject intolerance assessed by the investigator.

Clinical tumor imaging evaluation will be performed once every 6 weeks (± 7 days) as per RECIST v1.1/iRECIST. From 48 weeks after the first dose, clinical tumor imaging evaluation will be performed once every 12 weeks (± 7 days). If the subjects develop PD (PD) or have no clinical benefits during the dosing period, treatment is ended and the subjects enter the follow-up period.

The subjects will receive study treatment until PD (judged by the investigator as per RECIST v1.1/iRECIST), with no clinical benefits, or meeting other withdrawal criteria for subjects, whichever occurs first. The subjects will receive treatment with QL1706 for 2 years in maximum.

The subjects who develop PD (PD per RECIST v1.1 is currently iUP per iRECIST) may continue treatment with the investigational drug if, the subjects are clinically stable and the benefits of continuing treatment outweigh the risks according to the investigator's judgment. PD should be confirmed by imaging examination (with an interval of ≥ 4 weeks and ≤ 8 weeks from the previous imaging examination as per iRECIST). Treatment with QL1706 should be discontinued in case of confirmed PD (iCPD as per iRECIST criteria). Note: Chemotherapeutic drugs and bevacizumab should be discontinued at the first occurrence of PD.

# STUDY POPULATION

## Inclusion Criteria

Subjects must meet all of the following inclusion criteria to be eligible for study participation:

1. Subjects voluntarily participate in the study and sign the ICF.
2. Subjects are aged ≥ 18 years when signing the ICF, with no gender restrictions.
3. Eastern Cooperative Oncology Group (ECOG) performance status score of 0–1.
4. Expected survival time ≥ 3 months.
5. Histologically- or cytologically-confirmed Stage ⅢB/C or IV NSCLC (classified according to 8th edition of AJCC) patients, including squamous and non-squamous cancer. Details can be found below.
   1. Cohort 1: Stage ⅢB/C (unsuitable for radical surgery or other radical local therapy) and Stage IV squamous NSCLC patients, without previous systemic anti-cancer treatment for Stage IIIB/C or metastatic disease. Those who have previously received neoadjuvant/adjuvant therapy for non-metastatic disease, or radical chemoradiotherapy, may be enrolled if the last treatment is > 6 months from the start of study treatment.
   2. Cohort 2: Stage IIIB/C (unsuitable for radical surgery or other radical local therapy) and Stage IV non-squamous NSCLC patients, without previous systemic anti-cancer treatment for Stage IIIB/C or metastatic disease. Those who have previously received neoadjuvant/adjuvant therapy for non-metastatic disease, or radical chemoradiotherapy, may be enrolled if the last treatment is > 6 months from the start of study treatment. But the following are excluded: (1) patients carrying sensitive EGFR gene mutation should meet any of the following conditions: no 20 exon T790M mutation after failing (treatment failure: occurrence of radiographic progression or toxicity intolerance during treatment or after treatment completion as judged by the investigator as per RECIST v1.1) prior first or second-generation EGFR-TKI therapies (including gefitinib, erlotinib, icotinib, afatinib, etc.); experiencing 20 exon T790M mutation after failing prior first or second-generation EGFR-TKI monotherapy, followed by recurrence of PD after receiving osimertinib or other third-generation EGFR-TKI therapies; subjects failing prior osimertinib or other third-generation EGFR-TKI therapies (as first-line treatment) (regardless of EGFR T790M mutation status). If the subjects show other sensitive mutations (except exon 19 deletion mutation and exon 21 L858R mutation), including 18G719X, 20S786I, and 21L861Q mutations, apart from the aforementioned conditions, subjects should also have failed EGFR-TKI treatment after previous response (per Jackman criteria: CR/PR or SD ≥ 6 months) to EGFR-TKI treatment. (2) Patients carrying anaplastic lymphoma kinase (ALK)-fused oncogene have to experience PD or be intolerant to ALK-TKI during or after ALK-TKI (e.g., crizotinib) therapy. Note: TKI wash-out period for all participants is 1 week or 5 half-lives after the last treatment, whichever is longer.
6. Patients with at least one imaging measurable lesion per RECIST v1.1. Tumor lesions with clear radiographic progression after previous radiotherapy (or other local treatments) are also considered measurable lesions.
7. Prior to enrollment, subjects in Cohort 2 should provide a test report on molecular pathology of tumor tissue, including assay of EGFR/ALK and other genes.
8. Organs must exhibit adequate functional level prior to the first dose of study treatment (transfusion with any blood components and drugs that stimulate the growth of WBC or platelets are not allowed within 7 days before obtaining laboratory test results).
   1. WBC ≥ 3 × 10^9^/L.
   2. Absolute neutrophil count ≥ 1.5 × 10^9^/L.
   3. Platelet count ≥ 100 × 10^9^/L.
   4. Hemoglobin ≥ 90 g/L.
   5. Serum creatinine ≤ 1.5 × ULN or creatinine clearance (CLcr) ≥ 50 mL/min calculated according to the Cockcroft-Gault equation.
   6. Total bilirubin ≤ 1.5 × ULN (< 3 × ULN is permitted for patients with Gilbert's syndrome).
   7. AST and ALT ≤ 2.5 × ULN (≤ 5 × ULN is permitted for patients with liver metastases).
   8. INR or APTT ≤ 1.5 × ULN.
   9. Left ventricular ejection fraction (LVEF) > 50%.
9. Subjects (both female and male) must employ effective contraceptive measures from the signing of the ICF to 180 days after the last dose of the investigational products. Women who are pregnant or lactating (who can be enrolled if she agrees to stop breastfeeding during this period) from the signing of the ICF to 180 days after the last dose of the investigational products.

## Exclusion Criteria

Subjects who meet any of the following criteria will not be enrolled in this study.

1. CNS metastases with symptoms. Subjects may be enrolled if they fulfill the following conditions at least 2 weeks prior to the first dose of study treatment: Adequate treatment of CNS metastases and resolution of nervous system symptoms (other than residual signs and symptoms related to CNS treatment), and discontinuation or dose reduction of systemic corticosteroids (≤ 10 mg prednisone per day or equivalent).
2. Presence of carcinomatous meningitis prior to the first dose of study treatment.
3. Known active autoimmune disorder that requires systemic treatment within 2 years prior to the first dose of study treatment. But the following are excluded: related replacement therapies (such as thyroxine, insulin, or physiological corticosteroid replacement therapy for renal/pituitary insufficiency); skin diseases not requiring systemic treatment, such as vitiligo and psoriasis.
4. Diseases that require treatment with systemic corticosteroids (> 10 mg prednisone per day or equivalent) or other immunosuppressive drugs (cyclophosphamide, azathioprine, methotrexate, thalidomide, TNF-alpha inhibitors, etc.) within 2 weeks prior to the first dose of study treatment. Local corticosteroids, nasal spray, and inhalational steroids are allowed. Systemic corticosteroids as prophylaxis for contrast media allergy are allowed. Low-dose corticosteroids for orthostatic hypotension are allowed.
5. Concurrent diseases that may seriously affect patient safety or impact the completion of the study as determined by the investigator, such as hypertension that cannot be controlled by 2 or more types of anti-hypertensive drugs (systolic blood pressure ≥ 160 mmHg and/or diastolic blood pressure ≥ 100 mmHg) and severe diabetes.
6. Subjects with any of the following cardiovascular diseases shall be excluded.
   1. Acute myocardial infarction within 6 months prior to the first dose of the study treatment or patients with NYHA class III or IV heart failure.
   2. Poorly uncontrolled cardiovascular disease prior to the first dose of the study treatment, including angina, pulmonary arterial hypertension, or severe arrhythmia or conduction disorders.
   3. 12-lead ECG shows that mean QT interval (QTcF) > 450 ms (males) or > 470 ms (females) prior to the first dose of the study treatment.
7. Patients with previous or current interstitial lung disease, pneumoconiosis, radiation pneumonia, or severe lung function impairment, which may interfere with the detection and treatment of suspected treatment-related lung toxicities deemed by the investigator.
8. History of intestinal fistula, gastrointestinal perforation, or intra-abdominal abscess within 6 months prior to screening.
9. Uncontrolled pleural and peritoneal effusions, or pericardial effusion requiring repeated drainage (≥ 1 drainage/month).
10. Uncontrolled or symptomatic hypercalcemia.
11. Grade ≥ 2 peripheral neuropathy (CTCAE v5.0) prior to the first dose of study treatment.
12. Previous immunotherapies, including immune checkpoint inhibitory antibodies (anti-PD-1, PD-L1, CTLA-4 antibodies, etc.), agonistic antibodies (anti-ICOS, CD40, CD137, GITR, OX40, etc.), immune cell therapy, etc.
13. Severe hemorrhagic diathesis or coagulation dysfunction, or currently receiving anticoagulant therapy or thrombolytic therapy. Imaging shows signs of tumor invasion into large vessels [tumor has completely approached, surrounded, or invaded lumen of large vessels (such as pulmonary artery or superior vena cava)]. Two or more occurrences of hemoptysis [≥ 0.5 teaspoon (2.5 mL) per occurrence] within 3 months prior to screening. Thrombosis within 6 months prior to screening or excessive arterial/venous thrombosis, such as cerebrovascular accidents (including transient ischemic attack), deep vein thrombosis, pulmonary embolism within 1 year prior to screening.
14. Scheduled to receive anti-RANKL therapy [such as denosumab and its biosimilars] during the study treatment period.
15. Received > 30 Gy chest/lung radiotherapy within 6 months before the first study treatment; received palliative radiotherapy at other parts or other local therapies within 2 weeks before the first study treatment, and have not recovered from adverse reactions due to radiotherapy or other local therapies.
16. Underwent any surgery within 4 weeks before the first study treatment and have not recovered.
17. History of allogeneic hematopoietic stem cell transplantation or organ transplantation (except corneal transplantation).
18. Systemic infection or other serious infection requiring intravenous antibiotic treatment for > 7 days within 14 days before the first study treatment.
19. HIV-positive patients; patients known to have received tuberculostatic therapy within one year prior to initial study treatment; HBsAg-positive patients having HBV DNA at a level ≥ 2000 IU/mL or 10^4^ copies/mL; HCV antibody-positive and HCV RNA-positive patients (Note: HCV antibody-positive patients should be ruled out if HCV RNA could not be detected in the study site; in addition, if the results of HBsAg, HBV DNA, HCV antibody, and HCV RNA assays fail to meet the aforementioned requirements because the study site fails to perform qualitative/quantitative assays, then the aforementioned corresponding criteria would be met when values of these items are out of normal reference ranges in the study site and judged by the investigator to be clinically significant abnormalities).
20. Received live attenuated vaccine within 4 weeks before the first study treatment. However, scheduled inactivated vaccine during the study treatment period is allowed.
21. Patients with a known history of psychoactive drug abuse, alcohol abuse, or substance abuse; history of definitive neurological or mental disorder, including epilepsy and dementia.
22. Patients with other malignant tumors within 5 years before the first study treatment (except cured basal cell carcinoma of the skin, cervical carcinoma *in situ*, and papillary thyroid carcinoma).
23. Patients with a known history of allergy to macromolecular protein drug products or to QL1706 and any component of other investigational products.
24. Patients who participated in other clinical studies and used other investigational drugs within 28 days before the first study treatment.
25. Patients with added risks associated with the study or who may interfere with the interpretation of study results as determined by the investigator, or who are deemed unsuitable by the investigator and/or sponsor.

## Subject Withdrawal Criteria

According to GCP and ICH guidelines, all subjects have the right to withdraw from the study at any time with or without justification, and the subjects withdrawing from the study would not be discriminated against or retaliated and their medical interests would not be compromised. For subjects who withdraw from the study, the reason for withdrawal should be recorded. Subsequent evaluation should be carried out if the subject is willing and compliant, and the safety and efficacy data of that subject should be obtained as much as possible.

Subjects can withdraw from the study treatment due to any of the following circumstances, but should continue study participation according to the protocol.

1. Voluntary withdrawal from the study by the subject.
2. The subject develops intolerable adverse reactions and the investigator believes that the study treatment shall be discontinued.
3. The subject experiences PD (as determined by the investigator according to RECIST v1.1/iRECIST).
4. The subject completed 2 years of QL1706 treatment.
5. The subject commits severe protocol deviation and the investigator and/or sponsor believe that treatment should be discontinued.
6. Female subjects who become pregnant during the study treatment period.
7. Other reasons lead the investigator to believe that the subject is unsuitable to further study treatment.
8. The subject starts other new anti-cancer treatment.
9. The study discontinues.

## Study Premature Termination or Discontinuntion Criteria

Criteria for premature study discontinuation or suspension include the following items.

1. The regulatory authority, Institutional Review Board, sponsor, or investigator believes that there is a major safety risk with the investigational drug.
2. The sponsor may terminate this study due to scientific, medical, or ethical reasons but must fully consider the rights, safety, and health of enrolled subjects.
3. Other reasons that the sponsor or investigator determines to be unsuitable for study continuation.
4. The regulatory authority requests for trial termination due to other reasons.

The study can continue once aforementioned problems leading to study suspension are resolved and approvals from the sponsor and the Ethics Committee or regulatory authority are obtained.

# INVESTIGATIONAL PRODUCTS

## Drug Distribution

The investigational products will be directly sent to the study sites, and will be given to successfully screened subjects according to the confirmed study phase/dose group.

## Compliance

The dose/consumption and dosing time of investigational product infused into each subject in each treatment cycle should be documented in detail in the raw data and eCRF, while reasons for dose delay, dose interruption, etc. should also be documented in the raw data and eCRF.

In the event of dose interruption during drip of an investigational product, the investigator should evaluate the percentage of the dose received by the subject and record it in the raw data and eCRF.

Subject compliance with the study treatment and study protocol is voluntary. This includes blood sampling required for study evaluation and imaging examinations for tumor evaluation. If the subject fails to return for follow-up as scheduled or if the subject's compliance is poor, the study treatment can be terminated as per the judgment of the investigator or the sponsor.

## Overview of Investigational Products

### General information

#### Investigational drug

Name: QL1706 Injection

Manufacturer: Qilu Pharmaceutical Co., Ltd.

Dosage form: Injection

Route of administration: Intravenous infusion

Strength: 50 mg:2 mL

Storage: Stored and transported at 2–8 °C while protected from light, avoid freezing and rigorous shaking

#### Chemotherapeutic drugs

##### Pemetrexed

Generic name: Pemetrexed Disodium for Injection (subject to its package insert)

Manufacturer: Qilu Pharmaceutical Co., Ltd.

Dosage form: Lyophilized powder

Route of administration: Intravenous infusion

Strength: 0.2 g

Storage: Stored in a closed container in a cool dark place (protected from light at a temperature up to 20 °C)

Additional information: See details in the package insert

##### Carboplatin

Generic name: Carboplatin for Injection (subject to its package insert)

Manufacturer: Qilu Pharmaceutical Co., Ltd.

Dosage form: Injection

Route of administration: Intravenous infusion

Strength: 10 mL:100 mg

Storage: Protected from light and kept in a cool place (at a temperature up to 20 °C)

Additional information: See details in the package insert

##### Paclitaxel

Generic name: Paclitaxel Injection (subject to its package insert)

Manufacturer: Wuxi Taxus Pharmaceutical Co., Ltd.

Dosage form: Injection

Route of administration: Intravenous infusion

Strength: 5 mL: 30 mg

Storage: away from light and sealed at below 25 °C

Additional information: See details in the package insert

##### Bevacizumab injection

Generic name: Bevacizumab Injection (subject to its package insert)

Manufacturer: Qilu Pharmaceutical Co., Ltd.

Dosage form: Injection

Route of administration: Intravenous infusion

Strength: 100 mg: 4 mL

Storage: stored and transported at 2–8 °C, away from light, avoid freezing and rigorous shaking

Additional information: See details in the package insert

### Dose regimen

All investigational products are administered via intravenous infusion, every 21 days for a cycle, once on the first day of each cycle (Q3W, D1). If the full dose cannot be completed on the first day, the remaining drug can be administered on the next day. Treatment regimens are detailed as follows:

Table 25. Drugs to be received during study treatment and the dose sequence

| **Treatment group** | **Histologic type** | **Run-in period** | **Post run-in period** |
| --- | --- | --- | --- |
| Cohort 1 | Squamous NSCLC | QL1706 (5 mg/kg) + paclitaxel (175 mg/m^2^) + carboplatin (AUC5/6), 4 cycles | QL1706 (5 mg/kg) |
| Cohort 2 | Non-squamous NSCLC | QL1706 (5 mg/kg) + pemetrexed (500 mg/m^2^) + carboplatin (AUC5/6) ± bevacizumab  (15 mg/kg), 4 cycles | QL1706 (5 mg/kg) + pemetrexed (500 mg/m^2^) ± bevacizumab  (15 mg/kg) |

When chemotherapy is given, prophylactic medication should be administered according to local medical practice or corresponding package insert.

#### Investigational drug

QL1706: dosed by intravenous infusion, 5 mg/kg, once every 3 weeks. The dose regimen for run-in period is the same as that for post run-in period; when QL1706 and chemotherapy are given on the same day, QL1706 will be administered first, while chemotherapeutic drugs will be given 30 min after completion of QL1706 infusion; prophylactic medication of chemotherapeutic drugs, if any, will be administered 30 min after completion of QL1706 infusion, and then the chemotherapeutic drugs will be given. Refer to relevant SOPs for detailed method of administration.

#### Chemotherapeutic drugs and targeted therapy drugs

Confirmation of histologic type: 1) for patients with mixed histologic type, the final type depends on primary component; 2) patients with a histologic type of adenosquamous carcinoma should be classified as non-squamous cell carcinoma patients.

The doses and dosing cycles are listed as follows. Detailed dosing-related procedure/information is subject to local medical practice and/or package insert.

**Squamous NSCLC:**

Paclitaxel (175 mg/m^2^) + carboplatin (AUC 5 or 6 mg/mL/min) (according to the Calvert formula), 3 weeks per cycle, dosed on D1 in each cycle. Dosed for 4 cycles.

**Non-squamous NSCLC:**

Pemetrexed (500 mg/m^2^) + carboplatin (AUC 5 or 6 mg/mL/min) (according to the Calvert formula) ± bevacizumab (15 mg/kg), 3 weeks per cycle, dosed on D1 in each cycle. Dosed for 4 cycles (pemetrexed and bevacizumab are dosed in both run-in period and post run-in period).

**Calculation of carboplatin dose — Calvert formula**

Dose of carboplatin (mg) = Desired AUC value × [CrCl (mL/min) + 25]

Note: CrCL is calculated according to the Cockcroft-Gault equation, and if the result calculated as per the Cockroft-Gault equation is greater than 125 mL/min, then CrCl should be 125 mL/min. Example:

When desired AUC value of carboplatin is 6, the maximum dose of carboplatin (mg) = 6 × 150 = 900 mg

### Supply, packaging, and labeling of investigational products

The sponsor is responsible for providing a sufficient amount of investigational products to the study sites. All investigational products will be packaged and labeled according to corresponding regulatory requirements.

Investigational drug in this study is QL1706. Unified secondary packaging is adopted. Texts such as "For this clinical study only", the name, batch number, shelf life, and storage conditions of the investigational drug, and the name of the sponsor should be distinctly stated on the secondary packaging.

### Management of investigational products

Designated staff are responsible for management of the investigational products in this study.

After the investigational products are delivered to a study site, the designated drug management staff at the study site will check and receive the qualified drugs and fill out the Investigational Product Receipt. The receipt is in duplicate, with one kept by the sponsor and the other by the study site.

The study site should establish an Investigational Product Usage Form to document in detail the actual quantity of the investigational products dispensed to/returned from each subject. This form is in duplicate, with one kept by the sponsor and the other by the study site.

The investigator must ensure that all investigational products are used only on subjects participating in this study and no investigational products are transferred to those who are not participants of the clinical study.

The monitor is responsible for monitoring the supply, usage, and storage of the investigational products.

If the study site is authorized to destroy the investigational products, the investigator must ensure that destruction conforms to pertinent environmental protection regulations, department policies, and other pertinent clauses, and provide the destruction procedure. All destruction should be documented.

## Dose Modification

### Dose modification for QL1706

In this study, reduction of the dose of QL1706 is not permitted, and only dose interruption or permanent discontinuation is permitted.

If the dose of QL1706 is interrupted due to QL1706-related toxicity, discontinuation for up to 56 consecutive days (since the last dose) is permitted, otherwise QL1706 treatment should be discontinued; in the event of interruption of the dose of QL1706 due to toxicity not related to QL1706, if discontinuation lasts for more than 56 consecutive days, then discussion with the sponsor should be carried out before deciding whether QL1706 can be administered again or not.

If it is necessary to interrupt/postpone the administration of chemotherapeutic drugs/targeted therapy drugs due to toxicity of the chemotherapeutic drugs/targeted therapy drugs and, in the judgment of the investigator, QL1706 can be administered, then QL1706 should be dosed as scheduled.

#### Infusion-related reactions

During dosing, the subject's condition should be observed. If severe and life-threatening infusion-related reactions occur, use of the investigational products should be stopped. Infusion should be interrupted or infusion rate should be decreased for subjects who develop mild to moderate infusion-related reactions.

Infusion-related reactions should be managed based on the medical practices and guidelines of the test facility. The treatment recommendations for infusion-related reactions are shown below, which are provided for reference.

Table 26. Recommendations for the treatment adjustment of QL1706-related infusion-related reactions

| **CTCAE grade** | **Description** | **Clinical management** |
| --- | --- | --- |
| Grade 1 | Mild, temporary reaction; neither infusion interruption nor treatment required | - Reduce QL1706 infusion rate until symptoms disappear. - Observe at bedside and monitor closely until recovery. - Once the recovery from symptoms is achieved, the infusion rate of QL1706 can be adjusted to the original one. |
| Grade 2 | Treatment or infusion interruption required; rapid response to symptomatic treatment (anti-histamine, NSAIDS, sedatives, and infusion treatment); prophylactic medication ≤ 24 h | - Interrupt QL1706 infusion. - Intravenously infuse physiological saline; administer 50 mg diphenhydramine (or equivalent) and/or 325–1000 mg acetaminophen; observe at bedside and monitor closely until symptoms disappear. Corticosteroids can be administered when necessary. - After disappearance of symptoms, restart infusion at 50% of the original infusion rate. If no complications occur after 30 min, the infusion rate can be increased to 100% of the original rate. - Closely monitor the subject's condition. Immediately stop infusion if symptoms recur. It is recommended to administer 50 mg diphenhydramine; observe at bedside and monitor closely until symptoms disappear. - Subsequently, it is recommended that prophylactics be administered before infusion: 50 mg diphenhydramine (or equivalent) and/or 325–1000 mg acetaminophen, at least 30 min before dosing of QL1706. Corticosteroids can be given if necessary. |
| Grade 3 | Delayed symptom alleviation (e.g. symptomatic treatment and/or infusion interruption which does not result in rapid recovery); symptoms recur after improvement; sequelae requiring inpatient treatment. | - Interrupt QL1706 infusion. - After symptoms disappear, the investigator will determine whether to restart infusion. If the investigator determines that infusion can be restarted, restart infusion at 50% of the original infusion rate or according to the investigator's judgment. - If the investigator determines to continue the QL1706 for the subject, prophylactic medication is recommended before each infusion. - Intravenous drip of physiological saline and the following methods are recommended for treatment of the subjects: bronchodilators, subcutaneous infusion of 0.2–1.0 mg adrenaline (1:1000 solution) or slow intravenous infusion of 0.1–0.25 mg adrenaline (1:10000 solution); and/or intravenous infusion of 50 mg diphenhydramine and 100 mg methylprednisolone (or equivalent) when necessary. |
| Grade 4 | Life-threatening consequences; urgent intervention indicated | - Immediately stop QL1706 infusion and subsequent dosing of the drug. - Intravenous drip of physiological saline and the following methods are recommended for treatment of the subjects: bronchodilators, subcutaneous infusion of 0.2–1.0 mg adrenaline (1:1000 solution) or slow intravenous infusion of 0.1–0.25 mg adrenaline (1:10000 solution); and/or intravenous infusion of 50 mg diphenhydramine and 100 mg methylprednisolone (or equivalent) when necessary. - Observe at bedside and monitor closely until symptoms disappear. - It is recommended that the investigator should comply with the test facility's treatment guidelines for allergic reactions. |

#### Immune-related adverse events

Management of immune-related adverse events (irAEs) is a key aspect of safety and will be carried out based on local medical practice. This is combined with the risk/benefit status of subjects judged by the investigator to make a decision on dose interruption/permanent discontinuation/dose resumption. It is recommended that the recommendations proposed by the American Society of Clinical Oncology (ASCO) in the 2018 Clinical Practice Guidelines for Management of Immune-Related AEs in Patients Treated with Immune Checkpoint Inhibitor Therapy^[27]^ or its later versions be used as a reference. The grading criteria in the ASCO guidelines are mostly consistent with CTCAE v5.0. For detailed grade management recommendations, please refer to the ASCO guidelines.

The following information for reference is translated from an excerpt of general recommendations in the ASCO guidelines.

1. Immune checkpoint inhibitor therapy should be continued with close monitoring for grade 1 toxicities, with the exception of some neurologic, hematologic, and cardiac toxicities.
2. Hold immune checkpoint inhibitor therapy for most grade 2 toxicities and consider resuming when symptoms and/or laboratory values revert to grade 1 or less. Corticosteroids (initial dose of 0.5 to 1 mg/kg/d of prednisone or equivalent) may be administered.
3. Hold immune checkpoint inhibitor therapy for grade 3 toxicities and initiate high-dose corticosteroids (1 to 2 mg/kg/d of prednisone or equivalent). Corticosteroids should be tapered over the course of at least 4 to 6 weeks. If symptoms do not improve within 48 to 72 hours of high-dose corticosteroid, infliximab may be offered for some toxicities. When symptoms and/or laboratory values revert to grade 1 or less, rechallenging with immune checkpoint inhibitor therapy may be offered; however, caution is advised, especially in those patients with early-onset irAEs. Dose adjustments are not recommended.
4. In general, grade 4 toxicities warrant permanent discontinuation of immune checkpoint inhibitor therapy, with the exception of endocrinopathies that have been controlled by hormone replacement.

The following table lists the dose modification recommendations for some irAEs based on the ASCO guidelines as a reference.

Table 27. Dose modification recommendations for irAEs

| **Toxicity type** | **Adverse event** | **Grading per ASCO guidelines^[a]^** | **Dose Modification** |
| --- | --- | --- | --- |
| Hepatotoxicity | AST/ALT elevation and/or total bilirubin elevation | Grade 2 (3 × ULN < AST/ALT ≤ 5 × ULN and/or 1.5 × ULN < total bilirubin ≤ 3 × ULN) (baseline AST/ALT < 3 × ULN) | Interrupt QL1706^[b]^.  If at baseline, 3 × ULN < ALT/AST ≤ 5 × ULN, QL1706 treatment can continue when ALT/AST ≤ 5 × ULN, and QL1706 treatment should be permanently discontinued if AST/ALT elevation is more than 50% of baseline and persists for more than one week^[c]^. |
|  |  | Grade 3 (5 × ULN < AST/ALT ≤ 20 × ULN and/or > 3 × ULN < total bilirubin ≤ 10 × ULN) (baseline AST/ALT < 3 × ULN) | Permanently discontinue QL1706^[c]^.  If at baseline, 3 × ULN < ALT/AST ≤ 5 × ULN, QL1706 treatment can continue when ALT/AST ≤ 5 × ULN, and QL1706 treatment should be permanently discontinued if AST/ALT elevation is more than 50% of baseline and persists for more than one week^[c]^. |
|  |  | Grade 4 (AST/ALT > 20 × ULN and/or total bilirubin > 10 × ULN) | Permanently discontinue QL1706^[c]^. |
| Gastrointestinal toxicity | Diarrhea/colitis | Grade 2 | Interrupt QL1706^[b]^. |
|  |  | Grade 3 | Consider whether permanent discontinuation of QL1706 is necessary. |
|  |  | Grade 4 | Permanently discontinue QL1706^[c]^. |
| Lung toxicity | Pneumonitis | Grade 1 | QL1706 treatment is interrupted when radiographic evidence demonstrates pneumonitis progression^[b]^. Treatment can be resumed if CT scan and vital capacity show that pneumonitis has improved or resolved. If pneumonitis does not improve, treatment is carried out based on grade 2 pneumonitis. |
|  |  | Grade 2 | QL1706 treatment is interrupted^[b]^. |
|  |  | Grade 3 or 4 | Permanently discontinue QL1706^[c]^. |
| Renal toxicity | Nephritis | Grade 2 (2 × baseline < creatinine ≤ 3 × baseline) | Interrupt QL1706^[b]^. |
|  |  | Grade 3 (creatinine > 3× baseline or > 4 mg/dL) or grade 4 (life-threatening or dialysis indicated) | Permanently discontinue QL1706^[c]^. |
| Skin toxicity | Rash/inflammatory dermatitis | Grades 3 and 4 | Interrupt QL1706^[b]^. |
|  | Bullous dermatoses, Stevens-Johnson syndrome, or toxic epidermal necrolysis | Grade 2–3 | Interrupt QL1706^[b]^. |
|  |  | Grade 4 | Permanently discontinue QL1706^[c]^. |
| Endocrine toxicity | Hypophysitis | Any grade | Interrupt QL1706 treatment until acute symptoms are resolved. |
| Musculoskeletal toxicity | Myositis | Grade 2–4 | QL1706 treatment is interrupted^[a]^. |
| Others | Other irAEs | General recommendations | According to the general toxicity grading management principles in the ASCO guidelines, treatment should be continued with close monitoring for grade 1 toxicities; in the case of toxicity of grades 2–3, interruption of QL1706 treatment should be considered, and resumption or discontinuation of QL1706 treatment will be considered depending on risk-to-benefit ratio of the subjects; permanent discontinuation of QL1706 treatment is recommended with grade 4 toxicities, with the exception of endocrinopathies that have been controlled by hormone replacement. |
|  |  | First occurrence of other grade 3 AEs | It is recommended to refer to detailed ASCO recommendations, and interruption of QL1706 treatment is generally recommended^[a]^. |
|  |  | Recurrent grade 3 AEs | QL1706 treatment should be permanently discontinued after the same grade 3 AE recurs^[c]^. |
|  |  | Grade 2 myocarditis | Permanently discontinue QL1706. |
|  |  | Life-threatening AEs | Permanently discontinue QL1706. |
|  |  | Grade 4 AEs | Permanently discontinue QL1706^[c]^. |
|  |  | Daily treatment with ≥ 10 mg prednisone or equivalent for more than 12 weeks | Permanently discontinue QL1706^[c]^. |
|  |  | Grade 2 or 3 AEs that persist for ≥ 12 weeks | Permanently discontinue QL1706^[c]^. |

1. The grading criteria in the ASCO guidelines are mostly consistent with CTCAE v5.0. Refer to the ASCO guidelines for grade-based management recommendations.
2. Please use local medical practices as a basis and refer to the ASCO guidelines. The general recommendation is to resume treatment when the AEs resolve to grade 0/1 or baseline, or resume dosing when the investigator deems that the benefits outweigh the risks.
3. Please be subject to local medical practice, and refer to [b] if the investigator deems that the benefits of continuing with QL1706 outweigh the risks.

#### Non-immune-related adverse events related to the investigational drug

In the event of non-immune-related QL1706-related toxicities during the study, AEs will be managed mainly according to local medical practice, and depending on the risk/benefit status of the subject judged by the investigator, dose interruption/permanent discontinuation/dose resumption decision will be made, while the proposed dose modification recommendations shown in the following table are for reference only.

Table 28. Dose modification recommendations for non-immune-related QL1706-related AEs

| **AEs and severity (CTCAE v5.0)** | | **Dose Modification** |
| --- | --- | --- |
| First occurrence of grade 3 AEs^[a]^ | | Interrupt QL1706^[b]^. |
| Recurrent grade 3 AEs^[a]^ | | Permanently discontinue QL1706^[c]^. |
| Life-threatening AEs, or grade 4 AEs^[a]^ | | Permanently discontinue QL1706^[c]^. |
| Grade 2 or 3 AEs that persist for ≥ 12 weeks^[a]^ | | Permanently discontinue QL1706^[c]^. |
| AST/ALT increased  or total bilirubin increased | Grade 2 | Interrupt QL1706^[b]^. |
|  | Grade 3 | Permanently discontinue QL1706^[c]^. |
|  | Grade 4 | Permanently discontinue QL1706^[c]^. |
| Other QL1706-related AEs for which the investigator deems that interruption or permanent discontinuation of QL1706 is required. | | Interrupt^[b]^ or permanently discontinue QL1706. |

1. Except for endocrine disorders, laboratory test abnormalities alone with no related clinical symptoms or for which appropriate treatment is given for correction, or other AEs determined by investigators to be of no risk. The investigator will determine the benefits/risks to the subject before making a decision.
2. Dosing may be resumed when the AEs resolve to ≤ grade 1 or baseline, or when the investigator deems that the benefits outweigh the risks.
3. Refer to [b] if the investigator deems that the benefits of continued QL1706 treatment outweigh the risks.

### Dose modification of chemotherapeutic drugs

Modification to the dose of chemotherapeutic drugs is to address chemotherapy-induced toxicities, and will be performed mainly according to the package insert and/or local medical practice. In this study, recommendations for dose modification of chemotherapeutic drugs (detailed in the study-related SOP) are established for reference by the investigator, and depending on toxicity severities and benefit/risk assessments of the subjects, the investigator can modify related recommendations to improve the patient compliance to the maximum extent and allow the patient to have an opportunity to get supportive care.

### Dose modification of bevacizumab

Dose reduction of bevacizumab is not permitted in this study. In the event of any AEs requiring discontinuation of bevacizumab treatment, its dose will remain unchanged once such treatment is resumed.

If a patient experiences any SAEs or non-serious grade 3 or 4 AEs evaluated by the investigator to be bevacizumab-related, dosing of bevacizumab has to be interrupted. If the events resolve to ≤ grade 1, bevacizumab treatment can be restarted at the same dose level. If dosing of bevacizumab is postponed due to toxicities such that it is more than 42 days later than the next dosing time point, then the patient has to discontinue permanently bevacizumab treatment.

Pertinent time interval between the last dose of bevacizumab and major surgery is unknown. As bevacizumab has a half-life of about 21 days, elective surgery should be postponed where possible, but if necessary, bevacizumab treatment should be discontinued for ≥ 28 days prior to the surgery. Bevacizumab treatment should not be restarted at ≥ 28 days after surgery and before complete wound healing.

If the patient experiences dyspnea or clinically significant hypotension, bevacizumab infusion should be interrupted. For patients experiencing grade 3 or 4 anaphylaxis/hypersensitivity as per NCI CTCAE, adult respiratory distress syndrome, or bronchospasm (regardless of grade), bevacizumab treatment should be discontinued.

For patients developing any infusion-related symptoms not specified above, infusion rate of bevacizumab should be reduced until it is approximately ≤ 50% of the initial value or its infusion should be interrupted. If the infusion is interrupted, it can be resumed at ≤ 50% of the infusion rate prior to reaction after the patient's symptoms resolve fully, and if good tolerability is noted, the infusion can be accelerated to a full rate at an increment of 50%. In the next cycle, the infusion can be restarted at a full rate.

If the patient is found to have characteristics of thoracic vascular cavitation and/or tumor invasion at baseline, then the subsequent scans should also be reviewed to decide whether the discontinuation of bevacizumab treatment on the patient should be performed based on the scan manifestation.

Table 29 gives a summary of the dose modification criteria for bevacizumab treatment and toxicity management guidelines.

Table 29. Dose modification recommendations for bevacizumab-related AEs

| **Adverse event** | **Dose Modification** |
| --- | --- |
| **Hypertension** | |
| Grade 1 (asymptomatic, transient [< 24 h] blood pressure increased > 20 mmHg (diastolic blood pressure) or increase to a level > 150/100 mmHg [if the previous level is within normal reference range]) | Not modified. |
| Grade 2 (recurrent or persistent [> 24 h] or symptomatic increase > 20 mmHg (diastolic blood pressure) or increase to a level > 150/100 mmHg [if the previous level is within normal reference range]) | Bevacizumab treatment is discontinued. Anti-hypertensive therapy starts. Once the blood pressure < 150/100 mmHg, the patient can continue bevacizumab treatment. |
| Grade 3 | One or more anti-hypertensive agents or more potent therapy are needed: If blood pressure cannot be controlled at 150/100 mmHg by medication, then bevacizumab treatment is discontinued. |
| Grade 4 (including hypertensive encephalopathy) | Bevacizumab treatment is discontinued. |
| **Hemorrhage** | |
| Grade 1 or 2 non-pulmonary or non-CNS events | Not modified. |
| Grade 3 non-pulmonary or non-cerebral or non-myeloid hemorrhage | Bevacizumab treatment is interrupted until all of the following criteria are met:   - Hemorrhage is resolved, and hemoglobin level is stable. - Hemorrhage factors that will pose more risks to treatment are absent. - Anatomical or pathological conditions that will significantly pose more risks of hemorrhage recurrence are absent.   Bevacizumab treatment will be discontinued for patients with recurrence of any grade 3 hemorrhage events. |
| Grade 4 non-pulmonary or non-cerebral or non-myeloid hemorrhage | Bevacizumab treatment is discontinued. |
| Grade 1 pulmonary or cerebral or myeloid hemorrhage | Bevacizumab treatment is interrupted until all of the following criteria are met:   - Hemorrhage is resolved, and hemoglobin level is stable. - Hemorrhage factors that will pose more risks to treatment are absent. - Anatomical or pathological conditions that will significantly pose more risks of hemorrhage recurrence are absent. |
| Grade 2, 3, or 4 pulmonary or cerebral or myeloid hemorrhage | Bevacizumab treatment is discontinued. |
| Venous thromboembolism event |  |
| Grade 1 or 2 | Not modified. |
| Grade 3 or asymptomatic grade 4 events | If scheduled duration of full-dose anticoagulant therapy is < 2 weeks, then bevacizumab treatment should be interrupted until completion of the full-dose anticoagulant therapy. If scheduled duration of full-dose anticoagulant therapy is > 2 weeks and all of the following criteria are met, then bevacizumab treatment can be restarted after 2-week full-dose anticoagulant therapy:   - If warfarin is being used, then INR of the patient has to be within the reference range (normally 2–3); prior to restart of study treatment, dosing of LMWH, warfarin, or any other anticoagulants has to be stable. - When the patient is under anticoagulant therapy, no grade 3 or 4 hemorrhage events should occur. |
| Symptomatic grade 4 events | Bevacizumab treatment is discontinued. |
| **Arterial thromboembolism events** (newly diagnosed, worsened, or unstable angina, myocardial infarction, transient ischemic attack, cerebrovascular accident, and any other arterial thromboembolism events) | |
| Any grade | Bevacizumab treatment is discontinued. |
| **Congestive heart failure** (left ventricular systolic dysfunction) | |
| Grade 1 or 2 | Not modified. |
| Grade 3 | Bevacizumab treatment is discontinued until the symptoms resolve to ≤ grade 1. |
| Grade 4 | Bevacizumab treatment is discontinued. |
| **Proteinuria** | |
| Grade 1  (dipstick 1+ or urine collection 0.15–1.0 g/24 h) | Not modified. |
| Grade 2  (dipstick 2+ to 3+ or urine collection > 1.0–3.5 g/24 h) | For dipstick 2+, bevacizumab can be administered, and the urine in 24 h should be obtained prior to the next dose.  For dipstick 3+, the urine in 24 h should be obtained prior to dosing of bevacizumab.  If urine protein level is > 2 g/24 h, then bevacizumab treatment should be interrupted and its dosing should be restarted when urine protein level is ≤ 2 g/24 h^a^. |
| Grade 3 (dipstick 4+ or urine collection > 3.5 g/24 h) | Bevacizumab treatment is discontinued. Dosing is resumed when urine protein level is ≤ 2 g/24 h^a^. |
| Grade 4 (nephrotic syndrome) | Bevacizumab treatment is discontinued. |
| **Gastrointestinal perforation** | |
| Any grade | Bevacizumab treatment is discontinued. |
| **Fistulas** | |
| Tracheo-esophageal fistula of any grade | Bevacizumab treatment is discontinued. |
| Grade 4 fistulas (except tracheo-esophageal fistula) | Bevacizumab treatment is discontinued. |
| **Bowel obstruction** | |
| Grade 1 | For partial obstruction not requiring medical intervention, the patients should continue study participation. |
| Grade ≥ 2 | Bevacizumab treatment is discontinued. |
| **Wound dehiscence** | |
| Any grade (requiring medication or surgical treatment) | Bevacizumab treatment is discontinued. |
| **Reversible posterior leukoencephalopathy** | |
| Any grade (confirmed by MRI) | Bevacizumab treatment is discontinued. |

LMWH: Low molecular weight heparin. a All urine protein values were obtained from the urine collected in 24 h.

## Concomitant Treatments

### Previous medications and other treatments

All anti-lung cancer drugs and other treatments used before signing the ICF have to be documented in eCRF.

Minimally, other previous medications within 14 days prior to signing the ICF and a history of other treatments should be collected and documented in eCRF.

### Concomitant medications and other treatments

Concomitant medications and other treatments within a period since the subject signs the ICF until 90 days after the last dose of the investigational products or during initiation of a new anti-cancer treatment (whichever occurs first) should be documented. After the above collection period, AE-related concomitant medications and other treatments are subject to the rules for AE recording, as detailed in Section "Recording of adverse events".

Prohibited (or to be used with caution) or permitted drugs and other treatments from the signing of the ICF to the last dose of the investigational product are shown below.

#### Drugs and other treatments prohibited (or to be used with caution)

1. Any other anti-cancer drugs, such as chemotherapy drugs, immunotherapy drugs, hormone therapy drugs, monoclonal antibodies and other biologics, small molecule targeted anti-cancer drugs, and traditional Chinese medicine with anti-cancer indications, are prohibited.
2. Anti-cancer local treatments are prohibited, such as surgery, ablation, and radiotherapy (except for palliative radiotherapy, see "Permitted drugs and other treatments" for details).
3. Patients are prohibited from participating in other clinical studies or using other clinical study drugs or devices.
4. Live vaccine is prohibited within 4 weeks prior to the first dose of the investigational product and during study treatment, but inactivated vaccine during the treatment period is allowed.
5. Systemic corticosteroids (> 10 mg prednisone per day or equivalent) or other immunosuppressive drugs (e.g., cyclophosphamide, azathioprine, methotrexate, thalidomide, TNF-alpha inhibitors, etc.) are prohibited within 2 weeks prior to the first dose of the investigational product and during treatment period. Unless used as prophylaxis or for the treatment of adverse events. Refer to "Permitted drugs and other treatments" for details.
6. Anti-RANKL therapy (such as denosumab or its biosimilar) is prohibited during study period.

**Use of drugs that are prohibited (or to be used with caution) during chemotherapy is subject to specific instructions and local medical practices.**

#### Permitted drugs and other treatments

1. The investigator can decide the treatment for pain, allergies, infections, and other complications of malignant tumors and treatment-related AEs according to local medical practices.
2. With regards to QL1706, the investigator can administer prophylactic/therapeutic anti-infusion reaction/allergic reaction drugs after the first dose of QL1706. There is no restriction for chemotherapy drugs.
3. Immunosuppressants are allowed as prophylaxis or for the treatment of AEs (such as prophylactic drugs prior to chemotherapy specified in this study); local corticosteroids, nasal spray and inhalational steroids are allowed. Systemic corticosteroids are allowed as prophylaxis for contrast agent allergy. Relevant replacement therapies (such as thyroxine, insulin, or corticosteroid replacement therapy for renal/pituitary insufficiency, etc.) are allowed; low-dose corticosteroids for orthostatic hypotension are allowed.

[Notes]: The effect of corticosteroids on T cell proliferation has the potential to eliminate early QL1706-mediated antitumor immune activity. Therefore, when corticosteroids are required as prophylaxis in chemotherapy, it is recommended to reduce dexamethasone dose to clinically feasible levels. For example, if subjects do not develop hypersensitivity reactions after Cycle 1, reduction of dexamethasone dose is recommended to be considered, as permitted by local standards of care.

1. Palliative radiotherapy for non-target lesions is allowed. The investigational products may be suspended during palliative radiotherapy at the investigator's discretion.
2. Bisphosphonates are allowed for subjects with bone metastases.
3. Long-term medication for comorbidities (such as hypertension, hyperlipidemia, and diabetes mellitus).
4. For subjects with active hepatitis B, antiviral treatment (except interferons; nucleoside analogs are recommended) can be carried out according to local medical practices and/or diagnosis and treatment guidelines. It is recommended that antiviral treatment be started before the first dose of QL1706 and continue until the patient has recovered to a stable state.

# STUDY PROCESS

## Overview of the Study Process

For the requirements or observation parameters of each study procedure, refer to "Detailed Description of Various Test Items in the Study Process". Additional descriptions are provided below.

### Imaging examination of tumor lesions

Tumor response is evaluated by imaging at the time points stipulated in the protocol.

Identical imaging methods should be used for baseline and subsequent evaluations. All lesions found at the baseline period or subsequent suspected new lesions must be examined at appropriate time points. Unscheduled imaging examinations may be carried out if a PD is suspected.

## Study Procedures at Various Time Points

For the completion time points for all evaluations conducted after study commencement (i.e. evaluations after Day 1 of administration of the investigational drug), please refer to Study Window Management.

The safety and tolerability of subjects will be closely monitored and toxicity will be assessed before each dosing. The investigational products will only be administered when clinical evaluation and local laboratory test results are acceptable.

### Study visit

The run-in period and post run-in period are carried out in sequence. For details on study visit schedule, please refer to the "Study Schedule".

To ensure subject safety, the investigator may increase the frequency of examinations (in addition to the time points described in the Schedule) when a subject develops an AE or when clinically indicated.

### Re-treatment after PD confirmed by imaging

Patients who develop PD (PD per RECIST v1.1, currently iUP per iRECIST) may continue treatment with the investigational drug if, the subject is clinically stable and the benefits of continuing treatment outweigh the risks according to the investigator's judgment. PD should be confirmed by imaging examination (an interval of ≥ 4 weeks and ≤ 8 weeks from the previous imaging examination according to iRECIST). Treatment with QL1706 should be discontinued in case of confirmed PD (iCPD as per iRECIST criteria).

**The following criteria are used as a basis by the investigator to determine if the subject is clinically stable:**

1. No significant decrease in ECOG score.
2. No significant worsening of tumor-related clinical symptoms.
3. There is no need to strengthen management of tumor-related symptoms (such as analgesia, palliative radiotherapy, and other palliative treatments).

**Investigators should do their best to document why iUPD is not confirmed according to iRECIST requirements. Possible reasons are listed below.**

1. Study treatment is discontinued but subjects did not undergo efficacy assessment or imaging examination.
2. No iCPD.
3. Subject death.
4. Other reasons.

### Unscheduled visit

The investigator can conduct unscheduled visits according to clinical needs. Additional tests can be conducted if an AE occurs and the investigator deems that additional tests are necessary in consideration of the subject's safety.

Unscheduled imaging examinations can be carried out if the investigator suspects that PD is present.

## Voluntary Withdrawal by the Subject

The subject can withdraw from the study at any time, including withdrawing informed consent and loss to follow-up.

Withdrawal of informed consent: It refers to subject withdrawal of consent for further contact or further information disclosure by previously authorized people. If possible, the subject should inform the investigator in writing of his/her decision on stopping the follow-up. The investigator should describe informed consent withdrawal in detail in the medical record, such as no longer consenting to receiving the investigational products, or also not consenting to conform to study procedures and/or follow-up after the treatment is completed. Life status (regardless of whether the subject is dead or alive) measurements should only be confirmed using publicly available information if allowed by local laws. It should be noted that in contrast to withdrawal of informed consent by the subject, subjects that discontinue the study treatment due to other reasons will be retained in the study and follow-up must be carried out according to the specific follow-up steps stipulated in the protocol.

Loss to follow-up: The investigator should make the greatest reasonable efforts to locate the subject to determine and report his/her current status, including locating and interviewing people the subject authorized who can provide information on the subject. Definition of loss to follow-up: Failure to find the subject by 2 documented telephone numbers, fax, or email. All measures taken should be recorded in the subject's case history. The study site staff or representatives can access public resources, such as public health registration information and databases, to obtain the latest contact information. If the subject is still lost to follow-up after all these measures are taken, the date of the last confirmed status of the subject by the investigator will be recorded.

If the subject does not turn up for scheduled visits, greater efforts should be made to contact the subject. The investigator should ask about the reason for study withdrawal, request the subject to undergo subsequent follow-ups, and follow up with unresolved AEs.

If the subject refuses to participate in further follow-ups, the subject's status should still be continuously followed up unless the subject withdraws consent for disclosing further information or contact. Under these circumstances, no study evaluation will be carried out and no data will be collected. The sponsor may retain and continue to use all data obtained before the subject withdraws informed consent unless the subject requests the withdrawal of collected information.

## Study Termination and End of Study

### Study termination

This study will continue until completion or premature termination, which is determined by the decision of the regulatory authority, changes to the IRB/EC opinion, safety problems of the investigational drug, or based on the sponsor's judgment. In addition, Qilu Pharmaceutical Co., Ltd. reserves the right to terminate the R&D on QL1706 at any time.

If the study is prematurely terminated or interrupted, Qilu Pharmaceutical Co., Ltd. will inform the investigator in a timely manner. After the notification is sent, the investigator must contact all subjects as soon as possible. All study data should be collected and all raw data and eCRFs should be completed as much as possible under the guidance of Qilu Pharmaceutical Co., Ltd.

# SAFETY EVALUATION

## Adverse Event

### Definitions

#### Adverse event

An adverse event (AE) is defined as any untoward medical occurrence in a subject who received an investigational product, which can be manifested as any sign, symptom, disease, or abnormal laboratory finding, and which does not necessarily have a causal relationship with the investigational products.

Treatment-emergent adverse event (TEAE) refers to an adverse event that occurs or worsens in severity after the subject received the investigational product.

Safety information from the signing of the ICF to the start of study treatment is important for determining the safety of the investigational products (For example, symptoms that occurred prior to the use of the investigational drug can be used as baseline for before and after comparison); In addition, it can also aid in determining whether the safety information obtained is related to study procedures and operations (such as wash-out, biopsy, etc.). Therefore, the recording of AEs must start from **the signing of the ICF**.

The criteria for identifying medical history and AEs are presented below. The term "*condition*" may refer to abnormal physical examinations, symptoms, diseases, laboratory/auxiliary findings, or imaging assessments:

⦁ Conditions that existed before the signing of the ICF, and are asymptomatic or untreated at signing ICF should be documented as medical history (e.g., seasonal allergies without acute complaints).

⦁ Conditions that existed before the signing of the ICF, and are symptomatic or treated at signing ICF but without changes in severity should be documented as medical history (e.g., allergic pollinosis).

⦁ Conditions that existed before the signing of the ICF but are discovered or diagnosed during screening can be recorded as medical history based on the investigator's judgment (such as hyperlipidemia and cholelithiasis).

⦁ Conditions that develop or worsen after the signing of the ICF should be documented as AEs.

Any surgery scheduled by the doctor prior to the signing of the ICF should not be documented as an AE.

#### Serious adverse events

A serious adverse event (SAE) refers to an untoward medical occurrence that meets any of the following criteria:

1. Leading to death.
2. Life-threatening: "Life-threatening" refers to an immediate risk of death to the subject at the time of the event; it does not refer to an event that hypothetically might cause death if it were severer.
3. Leading to hospitalization or prolonged hospitalization.

If any of the following is met, the hospitalization or prolonged hospitalization is not required to be reported as an SAE:

**-** Hospitalization for less than 12 h;

**-** Scheduled hospitalization (e.g., a part of the study procedures; or hospitalization for safety observation/blood sample collection in this study);

**-** Hospitalization unrelated to AE (e.g., if admitted to a social welfare institution due to temporary care).

However, it is important to note that SAEs that meet other seriousness criteria during hospitalization are to be reported as SAEs based on clinical judgment.

1. Resulting in permanent or significant disability/incapacity. Disability refers to substantial damage to an individual's ability to conduct daily life functions.
2. Resulting in congenital anomalies or birth defects.
3. Other important medical events: The events that may not immediately result in death, threaten life, or lead to hospitalization/prolonged hospitalization, but may jeopardize the subject and may require medical intervention to prevent one of the above outcomes based on medical judgment. For example, allergic bronchospasm requiring treatment in an emergency room or home first aid, cachexia or convulsions not resulting in hospitalization, and development of drug dependence or drug abuse.

#### Suspected unexpected serious adverse reaction (SUSAR)

SUSAR refers to an adverse event that is unexpected, serious, and related to treatment. The nature and/or severity of its clinical manifestation exceeds existing information, such as the Investigator's Brochure for an investigational drug, package insert of marketed drugs, or summary of product characteristics.

### Adverse event evaluation

All AEs will be evaluated and documented by the investigator according to the following categories. These categories are detailed as follows.

#### Seriousness

The seriousness of each AE will be evaluated based on the definition of SAE.

#### Severity

The severity of AE will be classified according to CTCAE v.5.0. When there is no corresponding entry in the CTCAE, the severity is assessed using the following criteria.

Grade 1: Mild, asymptomatic, or mild symptoms; abnormal clinical or diagnostic observations only; intervention not indicated.

Grade 2: Moderate; minimal, local, or noninvasive intervention indicated; limiting age-appropriate instrumental activities of daily life (such as preparing meals, grocery shopping, and using the telephone).

Grade 3: Severe or medically significant but not immediately life-threatening; resulting in hospitalization or prolonged hospitalization; resulting in disabilities; limiting self-care activities of daily life (bathing, dressing and undressing, feeding self, using the toilet, and taking medications), but not bedridden.

Grade 4: Life-threatening consequences; urgent intervention indicated.

Grade 5: Death related to AE.

#### Causality

##### Causality between AE and the investigational products

The investigator should determine the causal relationship between every AE documented in the eCRF and the investigational products. Causality assessment is based on the clinical judgment made from available information in the eCRF.

Assessment is carried out based on whether there is a reasonable causal relationship between the AE and the investigational product. There are 2 causality assessment results: "related" and "not related".

The reasons for "not related" may include the following points.

1. Presence of other more plausible explanations, such as underlying diseases or events that are common to the study population.

or

1. Unreasonable, e.g. the temporal relationship of AE onset and time of study treatment is not plausible or the causality is not plausible from a biological perspective.

An evaluation of "related" means that there is a plausible relationship between the AE and the study treatment, and the following important factors could be considered.

1. The sequence of dosing and adverse event occurrence: The adverse event should appear after administration of the investigational product. The drug exposure duration when the adverse event occurs should be included for consideration.
2. Recovery status of the subject after drug discontinuation (de-excitation) and recurs after the drug is used again (rechallenge). When evaluating the responses of the subject to de-excitation and rechallenge, the general clinical course of the adverse event should be considered.
3. Underlying diseases and comorbidity: The natural medical history, course of disease that is undergoing treatment, and all other diseases should be considered for evaluation of each adverse event.
4. Concomitant medication and treatment: Other drugs or treatments received by the subject should be evaluated to determine whether these factors resulted in the adverse event.
5. The pharmacological and pharmacokinetic characteristics of the investigational product: PK characteristics (absorption, distribution, metabolism, and elimination) of the investigational product and the subject's condition should be considered.
6. The adverse event is rare and is known to be strongly correlated to drug exposure (such as angioedema, liver damage, and Stevens-Johnson syndrome).
7. In case causality cannot be assessed, the assessment result will be "related".

Table 30. Criteria for causality assessment

| **Causality documentation** | | **Reference assessment criteria (all criteria need not to be simultaneously met)** |
| --- | --- | --- |
| Related  (Possibility of plausible causal relationship between the event and the drug) | Definitely related | There is a reasonable temporal relationship between drug administration and the AE. The possibility that the AE is caused by the drug is greater than other factors (such as underlying diseases, comorbidities, concomitant medications, etc.). The AE improves or disappears on drug discontinuation and reappears when the drug is given again. The AE is a recognized pharmacological phenomenon or adverse reaction of the drug. |
|  | Possibly related | There is a reasonable temporal relationship between drug administration and the AE. The possibility that the AE is caused by the drug is greater than or is comparable to other factors (such as underlying diseases, comorbidities, concomitant medications, etc.). The AE improves or disappears on drug discontinuation. |
|  | Undetermined | The causal relationship between the AE and the investigational drug cannot be determined due to a lack of sufficient information. |
| Not related  (Possibility of absence of plausible causal relationship between the event and the drug) | Unlikely related | There is an unreasonable temporal relationship between drug administration and the AE. The possibility that the AE is caused by other factors (such as underlying diseases, comorbidities, concomitant medications, etc.) is greater than that caused by the drug. The AE does not improve or disappear on drug discontinuation. The AE does not reappear when the drug is given again or the rechallenge response is unclear. |
|  | Not related | The subject is not treated with the drug or the temporal relationship between drug administration and the AE is unreasonable, or the AE can be clearly caused by other factors. |

##### Causality between an AE and the study procedure

Assessment of the causality between AE and the study procedure should be conducted on the basis of whether a plausible causal relationship exists between the AE and the procedure required in the protocol. The assessment results will be referenced from the causality assessment of the AE and the investigational products.

#### Measures taken on investigational products

Any measures targeting the investigational products taken during the study to mitigate AEs should be recorded according to the following categories:

**-** Unchanged.

- Drug suspension.

- Dose reduction ("dose reduction" is not allowed for QL1706 or bevacizumab).

- Permanent discontinuation.

- Not applicable.

- Unknown.

#### Measures employed for adverse events

Targeted treatment for AEs during the study must be documented according to the following format:

- None.

- Drug treatment.

- Other treatment measures.

#### Outcome

The outcomes of AEs should be recorded according to the following criteria:

**-** Recovered/resolved.

- Improved.

- Recovered/resolved with sequelae.

- Not resolved.

- Death.

- Unknown.

### Recording of adverse events

The duration of adverse event collection lasts from the signing of the ICF to 90 days after the last dose of the investigational product or start of a new anti-cancer treatment (whichever occurs first).

All AEs that occur during the AE collection period should be completely documented in the subject's eCRF, including AEs observed by the investigator, obtained from inquiries, or reported by the subject. Abnormal laboratory test results and signs/symptoms that are clinically significant should be recorded as AEs.

The investigator is required to follow up all AEs until the events are resolved, return to baseline, become stable, or until the start of a new anti-cancer treatment or more information cannot be obtained. The investigator must monitor and document the outcome of AEs in the source documents. After the AE collection period, the investigator will only be required to report and follow up on new SAEs that are suspected to be related to study treatment.

The name of an AE should be a medical term and a medical diagnosis is preferred. In case a definitive diagnosis is not possible, the symptoms/vital signs are used. In case a diagnosis is confirmed later on, the record should be updated to replace the symptoms/vital signs with the diagnosis. When determining the name of an AE, it should be ensured that the name of each AE includes only a single event. A single diagnosis, vital sign/symptom is an AE.

The investigator is responsible for classifying each AE according to the aforementioned severity. At the same time, the investigator should assess the **seriousness** of the event. If it is an SAE, it should be filled out according to SAE criteria in the eCRF.

All AEs should be documented in detail, including the start and end date, severity, causal relationship between the investigational products and/or study procedures, probably alternative explanation (complications, concomitant medications), action taken toward the study treatment, treatment measures taken for the AE, and the final outcome of the AE.

In the event of a death, the cause of the death, rather than "death" itself, should be documented as the name of the AE. "Death" should be documented as the outcome of the AE.

The sponsor is required to conduct a separate evaluation of all serious adverse events (SAEs), including the expectedness, severity, and causal relationship with the investigational drug.

In this study, the investigator also needs to specify whether the AE is an immune-related adverse event.

### Progressive disease

During the AE collection period, clear PD of the tumor (including related symptoms and signs) is not documented or reported as AE; clear PD of the tumor conforming to the SAE criteria (including hospitalization/prolonged hospitalization, life-threatening condition, death, etc.) is not documented or reported as SAE, either.

### Reporting of serious adverse events

**Notifying the sponsor**

The investigator is responsible for reporting all serious adverse events that meet the SAE criteria specified in the protocol to the sponsor's drug safety department. The investigator must report the SAE to the sponsor immediately after becoming aware of the event, in the format specified by the sponsor (no later than 24 h after becoming aware of the event). All investigators must receive relevant training. Relevant contact information, report forms, and other information will be summarized in the investigator's management documents and updated when necessary.

The investigator is required to follow up all SAEs until the event is resolved, returns to baseline, becomes stable, or until the start of a new anti-cancer treatment or more information cannot be obtained. The investigator should submit an SAE follow-up report after obtaining new information. The time limit and format of the report should be the same as those of the initial report.

**Submitting to regulatory authorities**

The sponsor must manage all events (such as SAEs and SUSARs) in accordance with regulatory requirements, and submit SUSAR reports to regulatory authorities in accordance with current regulatory requirements.

**Notifying the investigator/clinical study site/Ethics Committee**

The sponsor should notify the investigator/clinical study site/Ethics Committee of relevant safety events (such as SUSARs) according to regulatory requirements. The specific requirements of various units should be referenced for the submission time limit and format.

### Safety reference documents

The reference document for the investigational drug in this study is the latest version of the Investigator's Brochure (IB). The reference documents for the use of marketed products are the latest version of the instructions.

The current IB provides a tabulated summary of common events identified so far during the clinical development of the drug. If new and significant safety information becomes available, the IB will be updated with the information and distributed to all participating study sites.

The sponsor will determine whether the AEs are expected based on the corresponding reference document (IB) and regulatory requirements.

## Pregnancy

If female subjects become pregnant after receiving treatment (and within AE collection period), the investigator is required to notify the sponsor regarding the pregnancy. The investigator should follow up the outcome of the pregnancy, and report any abnormalities in the mother or fetus.

If the partner of a male subject becomes pregnant, the above information regarding the course and outcome of the pregnancy and the health of the fetus should be obtained with the consent of the partner to the greatest efforts.

When reporting pregnancies, the report format specified by the sponsor should be followed. The investigator should report pregnancies within the same time limit as that for SAE to the sponsor, that is, within 24 h after becoming aware of the event.

Pregnancy outcomes such as spontaneous abortion, stillbirth, medical abortion, and fetal malformation (any congenital anomaly/birth defect/fetal growth restriction) are considered as SAEs, and the report forms specified by the sponsor should be filled and submitted within the required time limit.

# OTHER EVALUATIONS

## Efficacy Evaluation

### Efficacy evaluation criteria

The investigator will evaluate treatment response according to RECIST v1.1, in combination with iRECIST criteria. If symptoms suggest PD, imaging will be used in a timely manner to assess the patients to facilitate recording and confirmation of tumor response. In addition, clinical progression as judged by the investigators will be recorded in the eCRF.

### Efficacy endpoints

The following are the efficacy endpoints according to RECIST v1.1. Efficacy endpoints based on iRECIST are detailed in the Statistical Analysis Plan (SAP).

#### Progression-free survival

Progression-free survival (PFS) refers to the duration from the start of study treatment to the first occurrence of PD or death due to any cause (whichever occurs first).

#### Overall survival and 1-year overall survival rate

Overall survival (OS) refers to the duration from the start of study treatment to the time point when death occurs due to any cause. At the cut-off date, the cut-off analysis date will be used as the censor date for patients who are still under follow-up. For subjects who are lost to follow-up, the date of last contact will be used as the censor date. The 1-year OS rate is defined as the proportion of subjects who are still alive 1 year after the first dose of the investigational drug.

#### Objective response rate

Objective response rate (ORR) is defined as the best ORR during the study and includes patients with complete response (CR) and partial response (PR).

#### Duration of response

The duration of response (DOR) is defined as the duration from the first tumor assessment of CR or PR (whichever comes first) to the first assessment of PD or death.

#### Disease control rate

The disease control rate (DCR) refers to the percentage of patients with a best overall response evaluation of "CR + PR + SD".

## Evaluations of Pharmacokinetics and Immunogenicity

### Blood sampling

For details on blood sampling time points, please see "Study Window Management".

### Sample processing and storage

Specific requirements for sample processing and storage requirements of pharmacokinetics and immunogenicity tests are subject to the relevant SOPs.

### Analysis and evaluation

The validated test method will be employed to test anti-QL1706 antibodies, including the incidence of ADA and its titer, and the incidence of Nab.

When needed, plasma concentration of QL1706 will be measured using validated test method and be included in the population pharmacokinetic analysis to evaluate the impact of immunogenicity on QL1706 concentration in the blood.

## Exploratory Evaluation

The correlation between the biomarkers (such as TMB and PD-L1) in tumor tissues and the anti-tumor activity of QL1706 will be explored. Failure to collect/assess relevant samples (including blood samples and tumor tissue samples) for exploratory studies will not be regarded as protocol deviations.

Specific requirements for sample processing and storage are subject to the relevant SOPs.

# DATA MANAGEMENT

The data manager will draft the data management plan (DMP) according to the study requirements and manage all data according to the DMP, ensuring the reliability, integrity, and accuracy of the clinical trial data.

Data collection and management will be performed using the electronic data capture (EDC) system in this study. This system has been well validated, which can achieve storage of audit trails and management of accounts and permissions. The system manager will construct accounts for the data manager (DM), investigator, clinical research associate (CRA), and clinical research coordinator (CRC), which are given different permissions. In addition, the system manager will strictly manage and control the account application and cancellation.

The data manager will design the Case Report Form (CRF) based on the study protocol and by referencing the CDISC criteria, and draft the database construction and logic check descriptions according to the CRF. The database designer will construct electronic case report forms (eCRFs) in the EDC and design the logic check procedure. The data manager will test the database, which will be online after passing the test. All EDC users must have received adequate training and a user account prior to accessing the EDC database.

The investigator must collect subject data according to the GCP and the study protocol requirements. The investigator or CRC will record data in the EDC system in an authentic, accurate, complete, and standardized manner according to eCRF completion guidelines and save the data within the stipulated time. The saved data can still be directly amended in the EDC system. The logic check program of the EDC will automatically conduct system check. In the event of the data require to be checked by meeting the check conditions, system query will be triggered after the eCRF is saved. The investigator or CRC will check the saved data based on raw data and respond to the query, revise the data or confirm the accuracy of data, and make necessary explanations. The data manager will check the data revision results or query responses. Queries can be raised again when necessary until all queries are resolved or clarified.

During the trial, the CRA will confirm that all data in the EDC system are entered correctly and are consistent with raw data (source data/documents). The data manager will conduct manual review of data integrity, consistency, and accuracy. During the coding process, medical coders will conduct necessary reviews of clinical data required to be coded. Medical staff will conduct necessary medical reviews on clinical data. These four types of staff could submit manual queries in the EDC during source data comparison and data review. The investigator or CRC will reply to these manual queries in the system and revise the data when necessary. If the reply does not resolve the query, the reviewer can raise another query on that data until all queries are resolved or clarified. All data amendment records and query records are stored in the EDC audit trail.

After all queries have been resolved and no errors are found in data review, the principal investigator will sign electronic signature on all eCRFs in the EDC system and confirm data authenticity. In case there are amendments made to the data after signing the eCRFs, the amended eCRFs will require re-signing by the investigator.

After data cleanup, a data (blinded) review meeting will be held. During the meeting, the principal investigator, medical staff, statistician, data manager, and project manager will review the data, discuss and solve problematic data and questions to be solved, complete the final definition and delineation of the analysis population, and approve database locking by the data manager.

No modifications shall be made to the locked database in principle. If errors are still found in the data after database lock, the project team members (medical staff, statistician, data manager, and project manager) should assess the potential effects of these erroneous data on safety and efficacy analyses in detail and discuss how to manage these erroneous data. The erroneous data could be recorded in the Statistical Analysis Report and clinical report. If it is confirmed that there is a need for data revision after database lock, the project team members must sign relevant documents before the database could be unlocked for data revision. After data revision and re-cleanup are completed, the data manager will lock the database again. After locking, all data are ultimately exported by the data manager and submitted to the statistician for analysis.

After the study is completed, the data manager will complete the Data Management Report according to the actual implementation status of the project and complete the archival of hardcopy and electronic documents and data storage and backup. The EDC system manager will shut down the EDC (i.e., making it go offline).

# STATISTICAL ANALYSIS

## Sample Size Determination

This study plans to enroll 60 subjects, namely 20 to Cohort 1 and 40 to Cohort 2. Sample size estimation is not based on statistical assumptions. During the study, the number of subjects enrolled in each cohort will be promptly adjusted according to the efficacy and safety signals of each regimen.

## Statistical Analysis Datasets

- Full Analysis Set (FAS): Based on the intention-to-treat principle (ITT), the FAS includes all enrolled subjects who received at least one dose of the investigational drug. The FAS is mainly used to report subject demographic data, baseline characteristics, and efficacy analysis.
- Per-protocol Analysis Set (PPS): All subjects included in the FAS who met the protocol requirements, have adequate compliance and no major protocol deviations that affect efficacy analysis, will be used for efficacy analysis.
- Safety Set (SS): The safety set includes all enrolled patients who received at least one dose of the investigational products and have post-treatment safety records. The safety analysis set will be used for the analysis of safety data.
- Pharmacokinetics Concentration Set (PKCS): Includes all subjects with at least one evaluable QL1706 plasma concentration data point in the FAS and with no major violation of the study protocol that significantly affects PK assessment. This dataset is used for PK analysis.
- Immunogenicity Analysis Set (IAS): Includes all subjects in the FAS with at least 1 post-dose ADA test result. This dataset is used for ADA analysis.

## Statistical Analysis Plan

### General analysis

In this study, data will be summarized by descriptive statistics according to the following principles, unless otherwise stated.

Measurement data will be summarized as mean, standard deviation, median, maximum, and minimum. Enumeration data will be summarized as frequency and percentage. Kaplan-Meier estimator will be used for survival estimates based on time-event data and survival curves will be plotted.

Baseline is defined as the last non-missing effective data point before the first dose.

### Management of missing data

Subjects with missing post-treatment imaging evaluations will be reviewed one by one to determine the censor time when the investigator calculates variables involving time (such as PFS). Missing values will not be imputed for baseline and safety data, unless otherwise stated.

### Summary of study completion

Screening, enrollment, dropout and study completion status of each site, as well as the division of the corresponding statistical analysis population should be summarized. Dropout and excluded cases and the division of data sets should be listed. All participants that failed the screening should also be summarized, and the specific reasons for screening failure should be listed and described.

Exposure and treatment compliance: The duration, exposure dose, and treatment compliance of each subject who received the investigational product will be computed.

### Safety analysis

AEs are coded using MedDRA 21.1 or later. Statistical analysis will be carried out on treatment-emergent adverse events (TEAEs) in this study. AEs that occur before treatment will be listed. Unless otherwise stated, the AEs mentioned below are all TEAEs.

The analysis of AEs will be based on the SS. Data analysis will include but is not limited to: Numbers and incidences of adverse events/reactions, serious adverse events/reactions, adverse events/reactions leading to dropout, and adverse events/reactions leading to death, etc. Numbers and incidences of adverse events and adverse reactions will be sorted by system organ class, preferred term, and severity. Various adverse event cases will be listed in detail. The specific analyses are described in the statistical analysis plan.

In addition, for the various immune-related AEs and adverse reactions under various categories, the aforementioned AE summary analyses are also applicable. Refer to the statistical analysis plan for details.

The clinical laboratory markers, vital signs, physical examinations, and ECG examination results will be summarized and analyzed according to the observed values at the study visit and the changes from baseline. And the results of clinical significance judgment of laboratory tests will be summarized and analyzed in the form of a cross table involving pre-treatment and post-treatment values. Patients with abnormal laboratory markers, ECG, and abnormal physical examination results as well as the clinical interpretation will be presented as listings. For details, please refer to the statistical analysis plan.

### Efficacy analysis

Objective response rate (ORR): Calculate the number and percentage of subjects (with CR + PR) and determine the 95% CI using the Clopper-Pearson method.

Progression-free survival (PFS): Calculate median PFS and the 95% CI and censoring rate using the Kaplan-Meier method, then plot the survival curve.

Overall survival (OS): Same analytical method as that used for PFS.

Disease control rate (DCR): Calculate the number of cases and percentages of "CR + PR + SD". Same analytical method as that used for ORR.

Duration of Response (DOR): Same analytical method as that used for PFS.

### Evaluation of pharmacokinetics/pharmacodynamics data

Plasma concentration of QL1706 obtained in this study will be pooled with concentration data from other studies to construct a QL1706 population pharmacokinetic model using a nonlinear mixed-effects modeling for exploratory analysis. Evaluate the impact of various intrinsic/extrinsic factors on the pharmacokinetic properties of QL1706. Evaluate the potential correlation between AEs and QL1706 exposure and efficacy endpoints as derived from population pharmacokinetic analysis. For detailed analysis, refer to the population pharmacokinetic analysis plan. Analytical results will be reported separately.

### Immunogenicity analysis

The number and percentage of subjects who are ADA-positive and Nab-positive will be summarized by various scheduled collection time points defined in the protocol. For ADA-positive subjects, descriptive analysis of their antibody titer test results and changes relative to baseline will be carried out further.

ADA and Nab results of all subjects will be tabulated.

### Other analyses

Analysis of QL1706-related markers, such as certain biomarkers in tumor tissue samples. The analysis will be conducted on the full analysis set.

### End of study

Statistical analysis and trial summary can be carried out 18 weeks after the enrollment of the last subject. The study ends when 12 months have passed since the last subject has received the final dose or has reached 2 years of QL1706 treatment; study end time may be adjusted according to the actual progress of the project. In the event that subjects are still receiving the investigational drug at the end of the study, the sponsor shall continue to provide the investigational drug to these subjects, on the condition that there are clinical benefits as determined by the investigator and the subjects agree to continue treatment, until it is no longer clinically beneficial or the subjects develop adverse reactions due to intolerance. However, the sponsor will no longer provide the relevant examinations.

# INFORMED CONSENT, ETHICAL STANDARDS, AND REGULATIONS

## Informed Consent

The investigator or a designated representative must explain the clinical study in detail to the subject or his/her guardian and obtain a written ICF that is signed by the subject himself/herself or his/her guardian and the study physician. The final ICF should include the following content: study objective, study process and duration, study procedure that subjects must comply with (including invasive medical procedures), expected benefits and possibility of failing to receive benefits, potential risks or inconveniences to subjects, different groups that subjects may be assigned to, compensation and treatments that will be given when study-related injuries occur, confidentiality principles for personal data, etc. According to GCP, written approval for the ICF must be obtained from the corresponding Institutional Review Board. The informed consent process must adopt a language and texts that the subject or his/her guardian is able to understand and the subject must have access to the self-related information and data at any time during the study. The subject or his/her guardian will sign and date on the ICF; the investigator who implements the informed consent process is also required to sign and date on the ICF. If any important new data involving the investigational drug are found, written amendments to the ICF must be made and approved by the Institutional Review Board, and then the subject's consent must be re-obtained. The subject or his/her guardian should obtain the original signed and dated ICF or its copy and other provided written materials, including the original or copy of the amended ICF. Please refer to the GCP for other content about informed consent which is not covered in the protocol.

## Ethical Standards

This study protocol must be reviewed and approved in writing by the Institutional Review Board of the hospital before implementation. The Institutional Review Board should be provided with study protocols, protocol amendments, ICF, and other relevant documents such as recruitment advertisements. Written approval by the Institutional Review Board of the hospital must be obtained before the start of this study.

Without mutual consent, neither the sponsor nor the investigator may unilaterally modify this study protocol. Only changes or deviations intended to eliminate direct and immediate harm to the subjects may be made by the investigator without prior approval from the Institutional Review Board. At the same time, such changes or deviations and the corresponding reasons, as well as the proposed amendments to the protocol should be submitted to the Institutional Review Board for consideration at the earliest possible time. The investigator is required to explain and document any protocol deviations.

Any amendments to this protocol during the course of the clinical study should be submitted to the Institutional Review Board, and other study documents should be amended accordingly when necessary, and submitted and/or approved in accordance with the requirements of the Institutional Review Board. The investigator is responsible for submitting an interim report of the study regularly as required by the Institutional Review Board, and notifying the Institutional Review Board when the study has been completed.

## Regulations

According to corresponding Chinese regulations, an IND application to the NMPA must be made before starting a new drug clinical study and the clinical study can only be carried out after the IND approval is obtained. The clinical trial receiving number for QL1706 is CXSL1900118 (China).

This protocol has been designed in accordance with the following regulations:

- - 1. "Provisions for Drug Registration"
    2. "Good Clinical Practice"
    3. Consensus on ethical principles based on international ethical guidelines, including the "Declaration of Helsinki" and the ethical guidelines of the Council for International Organizations of Medical Sciences (CIOMS)
    4. ICH guidelines
    5. Other applicable laws and regulations

# STUDY MANAGEMENT

## Quality Control and Assurance

Study site qualification: The clinical study site should be a qualified institution for clinical trials of drugs.

Study staff qualification: Study staff must receive GCP training and work under the guidance of senior professionals.

Laboratory quality control measures: The laboratory must establish standard operating procedures and quality control procedures for laboratory observation parameters.

Clinical trial ward quality control measures: 1) Investigators (including nursing staff) must receive study protocol training before the start of the clinical trial. 2) The instrument is functional without defect, and can operate normally. 3) The drug is administered by professional nursing staff to subjects. 4) Dedicated staff are responsible for keeping the investigational products, which will be stored according to the conditions stipulated in the protocol. Remaining investigational products will be stored separately and the quantity of remaining drugs will be recorded. These drugs will be collectively returned to the sponsor or destroyed at the end of the clinical trial.

Monitoring: The sponsor will appoint a monitor to ensure that the rights and interests of subjects are protected, data in trial records and reports are authentic, accurate, and complete, and the trial complies with the approved protocol, GCP, and pertinent regulations.

Audit: The sponsor may entrust a third party or the sponsor's quality assurance department to audit the trial. The audit includes the supply of the drug, all trial documents required, documentation of the informed consent process, and consistency between case report forms and source documents. The content and scope of the audit can also be expanded accordingly. The investigators must agree to participate in the audit at a reasonable time and in a reasonable manner.

## Document Storage

According to GCP requirements, the sponsor as well as the investigators and medical institutions where they work at shall confirm that the necessary facilities and conditions for storing necessary clinical trial documents are possessed by both parties. The facilities for document storage should be protected from direct exposure to light, and be water-resistant, with an environment favorable for long-term storage. Regulations and SOPs for document management should also be established. The stored documents must be easily identified, searched, retrieved, and returned. If some documents generated during the clinical trial are not listed in the mandatory document management list created at various clinical trial stages, the sponsor, investigators, and study sites must also include them in their own mandatory document files for storage. Mandatory clinical trial documents must be archived for 5 years after the investigational drug is approved for marketing or 5 years after the clinical trial is completed.

## Publication of Study Results

All data and results of this trial belong to the sponsor. The investigator should strictly abide by the confidentiality agreement of this trial, and any paper or abstract related to the data of this trial must be submitted to the sponsor for review and written approval must be obtained from the sponsor before publication.

# RISK CONTROL

## Measures for Minimizing Conventional Risks

During the clinical trial, the rights, interests and safety of the subjects outweigh scientific and social benefits and the protection of subjects' rights and interests is strengthened in various aspects of the clinical trial.

1. Stringent protocol design

The protocol provides inclusion/exclusion criteria for subjects based on preclinical data and safety data from clinical studies of similar drugs, as well as interim safety data from Phase I study to ensure that subject selection is based on age, suitable histological type, performance status, physical status, organ function, laboratory tests, medical history, and comorbidities. Subjects with potential high risk from receiving the treatment are excluded. Such subjects include those with clinically significant cerebro-cardiovascular diseases, known human immunodeficiency virus infection, and pregnant and lactating women. Please refer to the preceding text for details on inclusion and exclusion criteria.

With regards to any suspected investigational drug-related adverse events that occur during the clinical study, the investigator should fully consider the benefit/risk ratio of the subject and carry out dose interruption/discontinuation. In case the dosing is interrupted, treatment with the investigational drug should continue after the subject has recovered to the stipulated level. The investigator will manage adverse events according to local medical practices. The investigator should also consider the risks/benefits of each subject when deciding whether to interrupt/permanently discontinue/resume treatment, or the investigator and the sponsor may decide together.

This study uses approved chemotherapy drugs and formulated recommended dose adjustments. During the clinical study, the investigator should fully consider the benefit/risk ratio of the subject and carry out dose modification/suspension/termination with reference to the package insert and local medical practices. If dosing is suspended, treatment with the investigational drug should continue after the subject has recovered to the stipulated level.

As stipulated in the protocol, subjects are allowed to withdraw from the study treatment when any confirmed PD (unless the investigator deems that there are persistent benefits) or intolerable toxicity is observed, when the subject requests for the withdrawal from the study, or when treatment discontinuation is judged by the investigator. The investigator can suggest or provide new or alternative treatment modalities to the subject based on the subject's actual condition. Subjects with no PD must undergo follow-up imaging evaluation until PD, death, loss to follow-up, withdrawal of informed consent, start of a new anti-cancer treatment, end of study, or 12 months after the last dose of the investigational products, whichever comes first.

Based on preclinical safety findings and safety data from clinical trials of similar drugs, we focus on immune-related AEs in the protocol design, such as the potential risks of developing immune-related hepatotoxicity, gastrointestinal toxicity, pulmonary toxicity, nephrotoxicity, cardiotoxicity, skin toxicity, endocrine toxicity, rheumatoid/skeletal muscle toxicity, neurotoxicity, and other immune-related adverse reactions. The protocol will exclude subjects with a high risk of such adverse reactions and provide risk prevention and recommended management measures for the study.

The design of safety evaluation parameters in the protocol covers adverse events reported with similar drugs and observations for toxicities in major organs such as the heart, liver, and kidneys. The criteria for individual subject withdrawal from the study and for clinical trial termination are also clearly stipulated in the protocol.

1. Selection of study sites

Medical institutions that have registered on clinical trial platforms will be selected and an expert with extensive clinical practice and clinical trial experience will be selected as the principal investigator. The principal investigator will guide the drafting of the study protocol and physicians with extensive clinical experience and relevant qualifications will be selected to care for subjects during the entire trial.

During the study, the investigator will prepare sufficient and essential treatment equipment, particularly resuscitation equipment, including drugs, devices, as well as staff, and maintain effective communication with the laboratory department and radiology department.

1. Pre-study training

Before the commencement of the study, the sponsor will conduct training for the investigators, study nurses, clinical research coordinators, and all relevant staff. The training content includes the Investigator's Brochure, study protocol, management and reporting of common adverse events, drug management, and SAE reporting requirements. In addition, the sponsor will dispatch professional clinical research associates to the participating sites for clinical monitoring to ensure that the study complies with the approved protocol and pertinent regulations.

1. Management of potential adverse events

During the study, the investigator will conduct subject consultation, physical examinations and/or laboratory tests at the time points stipulated in the protocol, and conduct additional consultations, physical examinations and/or laboratory tests when necessary.

For abnormal clinical performance and/or abnormal laboratory test results that occur after dosing, the investigator must determine the clinical significance in a timely manner. For these abnormalities, AE assessment of their correlations with the investigational drug, as well as whether corresponding treatment measures are required must be recorded.

The investigator must provide aggressive and effective treatments for all adverse events, regardless of their correlations with the investigational drug.

The investigator should inform the sponsor as soon as possible if any SAE occurs during the collection period for AEs. At the same time, the investigator should complete and sign the report form specified by the sponsor, which provides detailed descriptions on the time of onset of the SAE, severity, its relationship with the investigational drug, and the measures taken. At the same time, aggressive, effective, and necessary treatment measures are employed to ensure subject safety.

For unexpected serious adverse reactions that are determined to be definitely related or suspected to be related to the investigational drug according to the "Rapid Reporting Criteria and Procedure for Safety Data During Clinical Drug Trials", the sponsor will follow the above criteria and procedure to rapidly report to the relevant department through Case Safety Reports.

1. Notification and timely updating of drug safety information

The sponsor's pharmacovigilance department is responsible for monitoring clinical trial safety information and managing SAE reports, and formulating the standard operating procedure for monitoring clinical trial safety information and reporting SAEs. The pharmacovigilance department will obtain the latest safety information during the clinical trial and carry out safety risk assessment in a timely manner and report relevant information to trial-related parties.

## Possible Important Risks and Risk Minimization Measures

### Possible important risks

The management of immune-related adverse event is a key safety aspect, and when they occur they should be treated and managed promptly in accordance with medical practice and relevant guidelines. Local medical practices are used to manage immune-related AEs. This is combined with the risk/benefit ratio determined by the investigator to make a decision on drug interruption/permanent discontinuation. It is recommended that the suggestions proposed in the 2018 management practice guidelines (or newer versions) for immune-related AEs in patients receiving immune checkpoint inhibitors by the American Society of Clinical Oncology (ASCO) be used as reference. The protocol has listed in detail the dose modification suggestions when certain immune-related AEs occur. The grading criteria in the ASCO guidelines are mostly consistent with CTCAE v5.0. For detailed grade management recommendations, please refer to the ASCO guidelines. The following table lists possible risks and risk minimization measures. The grade-based management measures of corresponding immune-related AEs are organized mainly according to grading criteria from ASCO guidelines and correspond to dose modification content in relevant sections in the protocol.

Table 31. Possible risks and risk minimization measures

| **Class** | **Risk minimization measures** |
| --- | --- |
| Infusion-related reaction | - The infusion rate for QL1706 should be reduced when grade 1 infusion-related reactions occur. If symptoms resolve, the infusion rate for QL1706 can be adjusted to the original rate. - QL1706 infusion should be suspended when grade 2 infusion-related reactions occur. After symptoms disappear, 50% of the original infusion rate is used to restart infusion and it is recommended that prophylactics be used before infusion. - Suspend QL1706 infusion if grade 3 infusion-related reactions occur. If the investigator decides to continue QL1706 administration for the subject, prophylactic medication is recommended before each infusion. - When grade 4 infusion-related reactions occur, QL1706 infusion must be immediately stopped and subsequent administration of QL1706 will be canceled. |
| Immune-related hepatotoxicity | - Blood biochemistry tests are conducted during the screening period and total bilirubin should be ≤ 1.5×ULN and ALT and AST should be ≤ 2.5×ULN. For subjects with liver cancer or liver metastasis, only those with ALT and AST ≤ 5×ULN can be enrolled in the clinical study. - Patients with hepatitis B (HBV) DNA ≥ 2000 IU/mL or 10^4^ copies/mL and patients with positive HCV antibodies and positive HCV RNA are excluded from this study. - Hepatic function will be closely monitored during the study. If hepatic dysfunction occurs, the investigational drug should be suspended or discontinued and aggressive symptomatic treatment should be given. - For subjects with active hepatitis B, it is recommended to commence antiviral treatment prior to the first dose of QL1706 and continue until the patient has recovered to a stable state. |
| Immune-related diarrhea/colitis | - The investigator must make a detailed inquiry into the medical history of the subject during the screening period. Subjects with autoimmune disease or history of autoimmune disease that do not meet the inclusion criteria should not be enrolled in the clinical study. - During the clinical study, monitoring of signs and symptoms of immune-related colitis (such as abdominal pain, diarrhea, and hematochezia) should be strengthened. - QL1706 should be suspended when grade 2 immune-related diarrhea/colitis occurs. The investigator should consider whether permanent discontinuation of QL1706 is required when grade 3 immune-related diarrhea/colitis occurs. - QL1706 will be permanently discontinued when grade 4 immune-related diarrhea/colitis occurs. |
| Immune-related pneumonia | - The investigator must make a detailed inquiry into the medical history of the subject during the screening period. Subjects with autoimmune disease or history of autoimmune disease that do not meet the inclusion criteria should not be enrolled in the clinical study. - During the clinical study, monitoring of signs and symptoms of immune-related pneumonia (such as cough and chest discomfort) should be strengthened. Imaging methods (such as X-rays) will be used for examinations. - QL1706 will be suspended when grade 2 immune-related pneumonia occurs. - QL1706 will be permanently discontinued when grade 3/4 immune-related pneumonia occurs. |
| Immune-related nephritis | - The investigator must make a detailed inquiry into the medical history of the subject during the screening period. Subjects with autoimmune disease or history of autoimmune disease that do not meet the inclusion criteria should not be enrolled in the clinical study. - During the clinical study, monitoring of signs and symptoms of immune-related nephritis in patients should be strengthened. - QL1706 will be suspended when grade 2 immune-related nephritis occurs. - QL1706 will be permanently discontinued for patients who develop grade 3/4 immune-related nephritis. |
| Immune-related skin toxicity | - The investigator must make a detailed inquiry into the medical history of the subject during the screening period. Subjects with autoimmune disease or history of autoimmune disease that do not meet the inclusion criteria should not be enrolled in the clinical study. - During the clinical study, monitoring of signs and symptoms of immune-related skin toxicity should be strengthened. - QL1706 will be permanently discontinued for patients who develop grade 4 bullous dermatitis, Stevens-Johnson syndrome, or toxic epidermal necrolysis. |
| Immune-related endocrine toxicity | - The investigator must make a detailed inquiry on the medical history of the subject during the screening period. Subjects with autoimmune disease or history of autoimmune disease that do not meet the inclusion criteria should not be enrolled in the clinical study. - During the clinical study, monitoring of signs and symptoms of immune-related hypophysitis should be strengthened. - QL1706 will be suspended when immune-related hypophysitis of any grade occurs. |
| Immune-related musculoskeletal toxicity | - The investigator must make a detailed inquiry into the medical history of the subject during the screening period. Subjects with autoimmune disease or history of autoimmune disease that do not meet the inclusion criteria should not be enrolled in the clinical study. - During the clinical study, monitoring of signs and symptoms of immune-related musculoskeletal toxicity should be strengthened. - QL1706 will be suspended when grade 2–4 immune-related myositis occurs. |
| Other immune-related adverse reactions | - The ASCO guidelines will be used as a reference for other toxicity events. For items that are not listed in ASCO guidelines, the overall toxicity grade-based management principles in ASCO guidelines will be used as reference. - According to the overall toxicity grade-based management principles in ASCO guidelines, QL1706 should be suspended when grade 2–3 toxicity occurs. As for grade 3 toxicity, QL1706 treatment should be suspended and resumption of QL1706 should be based on a discussion about the subject's risk/benefit ratio. Grade 4 toxicity usually means that QL1706 should be permanently discontinued except when hormone replacement therapy is used to control endocrine AEs. |
| Pregnant or lactating women | - The protocol stipulates that pregnant or lactating women (who do not agree to stop breastfeeding) should not be enrolled in the clinical study. |
| Young patients | - The protocol stipulates that patients aged < 18 years should not be enrolled in the clinical study. |
| Patients with hepatic insufficiency | - Blood biochemistry tests are conducted during the screening period and total bilirubin should be ≤ 1.5×ULN and ALT and AST should be ≤ 2.5×ULN. For subjects with liver cancer or liver metastasis, only those with ALT and AST ≤ 5×ULN can be enrolled in the clinical study. - Patients with hepatitis B (HBV) DNA ≥ 2000 IU/mL or 10^4^ copies/mL and patients with positive HCV antibodies and positive HCV RNA are excluded from this study. - Hepatic function will be closely monitored during the study. If hepatic dysfunction occurs, the investigational drug should be suspended or discontinued and aggressive symptomatic treatment should be given. |
| Patients with renal insufficiency | - Blood biochemical tests are conducted during the screening period. Only subjects with creatinine ≤ 1.5×ULN or creatinine clearance ≥ 50 mL/min can be enrolled in the clinical study. - Renal function will be closely monitored during the study. If renal dysfunction occurs, the investigational drug should be suspended or discontinued and aggressive symptomatic treatment should be given. |

# REFERENCES

1. Sung H, Ferlay J, Siegel RL, et al. Global cancer statistics 2020: GLOBOCAN estimates of incidence and mortality worldwide for 36 cancers in 185 countries. CA Cancer J Clin, 2021.
2. Howlader N, Noone AM, Krapcho M, et al. SEER Cancer Statistics Review. 1975-2016, based on November 2018 SEER data submission, posted to the SEER web site, April 2019. Bethesda, MD: National Cancer Institute; 2019.
3. Rosell R, Karachaliou N. Large-scale screening for somatic mutations in lung cancer. Lancet, 2016, 387(10026): 1354-1356.
4. Chen DS, Mellman I. Oncology meets immunology: the cancer-immunity cycle. Immunity, 2013, 39(1): 1-10.
5. Beatty GL, Gladney WL. Immune escape mechanisms as a guide for cancer immunotherapy. Clin Cancer Res, 2015, 21(4): 687-692.
6. Grupp SA, Kalos M, Barrett D, et al. Chimeric antigen receptor-modified T cells for acute lymphoid leukemia. N Engl J Med, 2013, 368(16): 1509-1518.
7. Newick K, O'Brien S, Moon E, et al. CAR T cell therapy for solid tumors. Annu Rev Med, 2017, 68: 139-152.
8. Pitt JM, Marabelle A, Eggermont A, et al. Targeting the tumor microenvironment: removing obstruction to anticancer immune responses and immunotherapy. Ann Oncol, 2016, 27(8): 1482-1492
9. Postow MA, Callahan MK, Wolchok JD. Immune checkpoint blockade in cancer therapy. J Clin Oncol, 2015, 33(17): 1974-1982.
10. Baumeister SH, Freeman GJ, Dranoff G, et al. Coinhibitory pathways in immunotherapy for cancer. Annu Rev Immunol, 2016, 34: 539–573
11. Socinski MA, Bondarenko I, Karaseva NA, et al. Weekly nab-paclitaxel in combination with carboplatin versus solvent-based paclitaxel plus carboplatin as first-line therapy in patients with advanced non-small cell lung cancer: final results of a phase III trial. J Clin Oncol, 2012, 30(17): 2055-2062.
12. Hayashi H, Okamoto I, Morita S, et al. Postprogression survival for first-line chemotherapy of patients with advanced non-small-cell lung cancer. Ann Oncol, 2012, 23(6): 1537-1541.
13. Scagliotti GV, Parikh P, von Pawel J, et al. Phase III study comparing cisplatin plus gemcitabine with cisplatin plus pemetrexed in chemotherapy-naive patients with advanced-stage non-small-cell lung cancer. J Clin Oncol, 2008, 26(21): 3543-3551.
14. Paz-Ares LG, de Marinis F, Dediu M, et al. PARAMOUNT: Final overall survival results of the phase III study of maintenance pemetrexed versus placebo immediately after induction treatment with pemetrexed plus cisplatin for advanced nonsquamous non-small-cell lung cancer. J Clin Oncol, 2013, 31(23): 2895-2902.
15. Gandhi L, Rodriguez-Abreu D, Gadgeel S, et al. Pembrolizumab plus Chemotherapy in Metastatic Non-Small-Cell Lung Cancer. N Engl J Med 2018:378:2078-2092.
16. Garassino M, Rodriguez-Abreu D, Gadgeel s, et al. OA0406 Evaluation of TMB IN KEYNOTE-189 pembrolizumab plus chemotherapy vs placebo plus chemotherapy for nonsquamous NSCLC [abstract]. 2019 World Conference on Lung Cancer (WCLC). Barcelona, Spain: International Association for the Study of Lung Cancer (IASLC) 2019: Abstract: OA04.06.
17. Paz-Ares LG, Luft A, Tafreshi A, et al. Phase 3 study of carboplatin-paclitaxel/nab-paclitaxel (Chemo) with or without pembrolizumab for patients with metastatic squamous non-small cell lung cancer[abstract]. J Clin Oncol 2018; 36: abstract 105.
18. Socinski MA, Jotte RM, Cappuzzo f, et al. Atezolizumab for First-Line Treatment of Metastatic Nonsquamous NSCLC. N Engl J Med 2018:378:22882301.
19. Hellmann MD, Paz-Ares L, Bernabe Caro R, et al. Nivolumab plus Ipilimumab in Advanced Non-Small-Cell Lung Cancer. N Engl J Med 2019:381:2020-2031.
20. Reck M, Ciuleanu T-E, Dols MC, et al. Nivolumab (Nvo) +ipilimumab (IPD) + 2 cycles of platinum-doublet chemotherapy (chemo) vs 4 cycles chemo as first-line (1L) treatment (tx) for stage IV/recurrent non-small cell lung cancer(NSCLC): CheckMate 9LA [abstract]. J Clin Oncol 2020; 38:Abstract 9501-9501.
21. Hirsch FR, Bunn PA, Jr. EGFR testing in lung cancer is ready for prime time. Lancet Oncol 2009; 10:432-433.
22. Reck M, Mok TSK, Nishio M, et al. Atezolizumab plus bevacizumab and chemotherapy in non-small-cell lung cancer (IMpower150): key subgroup analyses of patients with EGFR mutations or baseline liver metastases in a randomised, open-label phase 3 trial. Lancet Respir Med 2019:7:387401.
23. Topalian SL, Hodi FS, Brahmer JR et al. Safety, Activity, and Immune Correlates of anti-PD-1 Antibody in Cancer. N. Engl. J.Med. 366(26), 2443–2454 (2012).
24. Yamamoto N, Nokihara H, Yamada Y, et al. Phase I study of Nivolumab, an Anti-PD-1 Antibody, in Patients with Malignant Solid Tumors. Invest New Drugs 35(2), 207–216 (2017).
25. Feng Y, Roy A, Masson E, et al. Exposure-Response Relationships of the Efficacy and Safety of Ipilimumab in Patients with Advanced Melanoma. Clinical Cancer Research An Official Journal of the American Association for Cancer Research, 2013, 19(14):3977-3986.
26. Hellmann M D, Rizvi N A , Goldman J W , et al. Nivolumab plus Ipilimumab as First-line Treatment for Advanced Non-small-cell Lung Cancer (CheckMate 012): Results of an Open-label, Phase 1, Multicohort Study. The Lancet Oncology, 2016, 18(1):31-41.
27. Brahmer JR, Lachetti C, Schneider BJ et al. Management of Immune-Related Adverse Events in Patients Treated With Immune Checkpoint Inhibitor Therapy: American Society of Clinical Oncology Clinical Practice Guideline. J Clin Oncol 36:1714-1768, 2018.

# APPENDICES

See below for details.

## Appendix 1 Eastern Cooperative Oncology Group Performance Status (ECOG PS) Score

| Scoring | Performance status |
| --- | --- |
| 0 | Fully active, able to carry on all pre-disease performance without restriction |
| 1 | Ambulatory and able to carry out work of a light or sedentary nature, e.g., light household work, office work, but not heavy physical activity |
| 2 | Ambulatory and capable of all self-care but unable to carry out any work activities; up and about more than 50% of waking hours |
| 3 | Capable of only limited self-care; confined to bed or chair for more than 50% of waking hours |
| 4 | Completely disabled, cannot carry on any self-care, totally confined to bed or chair |
| 5 | Death |

Oken M, Creech R, Tormey D, et al. Toxicity and response criteria of the Eastern Cooperative Oncology Group. Am J Clin Oncol. 1982;5:649-655.

## Appendix 2 New York Heart Association (NYHA) Functional Classification

Class I: Patients with cardiac disease but without resulting in limitation of physical activity. Ordinary physical activity does not cause fatigue, palpitation, dyspnea, or anginal pain.

Class II: Patients with cardiac disease resulting in slight limitation of physical activity. They are comfortable at rest, but ordinary physical activity results in fatigue, palpitation, dyspnea, or anginal pain.

Class III: Patients with cardiac disease resulting in marked limitation of physical activity. Less than ordinary activity causes fatigue, palpitation, dyspnea, or anginal pain.

Class IV: Patients with cardiac disease resulting in the inability to carry on any physical activity. Symptoms of heart failure may be present even at rest. Discomfort is increased when physical activity is undertaken.

## Appendix 3 Cockcroft-Gault Equation and Body Surface Area Calculation Formula

**Cockcroft-Gault Equation**

**Please choose the formula according to the test unit of serum creatinine:**

**If serum creatinine concentration is in mg/dL**

Creatinine clearance in men (mL/min) = $\frac{\text{（}\text{140-}\text{age)}\text{×body weight}}{\text{72×}\text{serum creatinine}}$

Creatinine clearance in women (mL/min) = $\frac{\text{0.85 ×}\text{（}\text{140-age)×body weight}}{\text{72×serum creatinine}}$

**If serum creatinine concentration is in μmol/L**

Creatinine clearance in men (mL/min) = $\frac{\left（ \text{140-}\text{age} \right）\text{×body weight}}{\text{0.81 ×serum creatinine}}$

Creatinine clearance in women (mL/min) =$\frac{\text{0.85 ×}\text{(}\text{140-age)×body weight}}{\text{0.81 ×serum creatinine}}$

**Note: Age is recorded in number of years; body weight is recorded as kilograms.**

**Calculation of body surface area (Xu Wensheng's formula)**

Body surface area (m^2^) = 0.0061 × height (cm) + 0.0128 × weight (kg) - 0.1529

Note: For results that exceed 2 m^2^, it is recommended to take 2 m^2^

## Appendix 4 Response Evaluation Criteria in Solid Tumors Version 1.1 (RECIST 1.1)

(Response Evaluation Criteria in Solid Tumors, RECIST v1.1)

(This appendix contains information that has been internally translated and shall be used for reference only. Please refer to the English original version during actual operations)

1 MEASURABILITY OF TUMOR AT BASELINE

- 1. Definitions

At baseline, tumor lesions/lymph nodes will be categorized as measurable or non-measurable as follows:

1.1.1 Measurable lesions

Tumor lesions: Must be accurately measured in at least one dimension (longest diameter in the plane of measurement is to be recorded) with a minimum size of:

- 10 mm by CT scan (CT scan slice thickness no greater than 5 mm)
- 10 mm caliper measurement by clinical exam (lesions that cannot be accurately measured with calipers should be recorded as non-measurable)
- 20 mm by chest X-ray
- Malignant lymph nodes: To be considered pathologically enlarged and measurable, a lymph node must be ≥ 15 mm in the short axis when assessed by CT scan (CT scan slice thickness recommended to be no greater than 5 mm). At baseline and in follow-up, only the short axis will be measured and followed.

1.1.2 Non-measurable

All other lesions, including small lesions (the longest diameter < 10 mm or pathological lymph nodes with ≥ 10 to < 15 mm short axis) as well as truly non-measurable lesions. Lesions considered truly non-measurable include: leptomeningeal disease, ascites, pleural or pericardial effusion, inflammatory breast disease, carcinomatous lymphangitis of skin or lung, abdominal masses that cannot be diagnosed and followed by imaging, and cystic lesions.

1.1.3. Special considerations regarding lesion measurability

Bone lesions, cystic lesions, and lesions previously treated with local therapy require particular comment:

Bone lesions:

- Bone scan, PET scan or plain films are not suitable for measuring bone lesions, but these techniques can be used to confirm the presence or disappearance of bone lesions;
- Lytic bone lesions or mixed lytic-blastic lesions, with identifiable soft tissue components, that can be evaluated by cross-sectional imaging techniques such as CT or MRI can be considered as measurable lesions if the soft tissue component meets the definition of measurability described above;
- Blastic bone lesions are non-measurable.

Cystic lesions:

- Lesions that meet the criteria for radiographically defined simple cysts should not be considered as malignant lesions (neither measurable nor non-measurable) since they are, by definition, simple cysts.
- 'Cystic lesions' thought to represent cystic metastases can be considered as measurable lesions, if they meet the definition of measurability described above. However, if non-cystic lesions are present in the same patient, these are preferred for selection as target lesions.

Lesions with prior local treatment:

- Tumor lesions situated in a previously irradiated area, or in an area subjected to other loco-regional therapy, are usually not considered measurable unless there has demonstrated progression in the lesion. Study protocols should detail the conditions under which such lesions would be considered measurable.

1.2 Specifications by Methods of Measurements

1.2.1 Measurement of lesions

All measurements should be recorded in metric notation, using calipers if clinically assessed. All baseline evaluations should be performed as close as possible to the treatment start and never more than 28 days (4 weeks) before the beginning of the treatment.

1.2.2 Method of assessment

The same method of assessment and the same technique should be used to characterize each identified and reported lesion at baseline and during follow-up. Imaging-based evaluation should always be done rather than clinical examination unless the lesion(s) being followed cannot be imaged but are assessable by clinical exam.

Clinical lesions: Clinical lesions will only be considered measurable when they are superficial and ≥ 10 mm diameter as assessed using calipers (e.g. skin nodules). For the case of skin lesions, documentation by color photography including a ruler to estimate the size of the lesion is suggested. As noted above, when lesions can be evaluated by both clinical exam and imaging, imaging evaluation should be undertaken since it is more objective and may also be reviewed at the end of the study.

Chest X-ray: Chest CT is preferred over chest X-ray, particularly when progression is an important endpoint, since CT is more sensitive than X-ray, particularly in identifying new lesions. However, lesions on chest X-ray may be considered measurable if they are clearly defined and surrounded by aerated lung.

CT, MRI: CT is the best currently available and reproducible method to measure lesions selected for response assessment. This guideline has defined measurability of lesions on CT scan based on the assumption that CT slice thickness is ≤ 5 mm. Measurable lesions should be at least twice the slice thickness if the CT slice thickness is greater than 5 mm. MRI is also acceptable in certain situations (e.g., for body scans).

Ultrasound: Ultrasound should not be used as a method to measure lesion size. Ultrasound examinations cannot be reproduced in their entirety for independent review at a later date and, because they are operator dependent, it cannot be guaranteed that the same technique and measurements will be taken from one assessment to the next. If new lesions are identified by ultrasound in the course of the trial, confirmation by CT or MRI is advised. MRI may be used instead if radiation exposure from CT is taken into account.

Endoscopy, laparoscopy: The utilization of these techniques for objective tumor evaluation is not advised. However, they can be useful to confirm complete pathological response when biopsies are obtained or to determine relapse in trials where recurrence following complete response or surgical resection is an endpoint.

Tumor markers: Tumor markers alone cannot be used to assess objective tumor response. If markers are initially above the upper normal limit, however, they must normalize for a patient to be considered in complete response. Because tumor markers are disease-specific, instructions for their measurement should be incorporated into protocols on a disease-specific basis. Specific guidelines for both CA-125 response (in recurrent ovarian cancer) and PSA response (in recurrent prostate cancer) have been published. In addition, the Gynecologic Cancer Intergroup has developed CA-125 progression criteria which are to be integrated with objective tumor assessment for use in first-line trials in ovarian cancer.

Cytology, histology: These techniques can be used to differentiate PR and CR in rare cases required by protocol (e.g., residual lesions in tumor types such as germ cell tumors, where known residual benign tumors can remain). When effusions are possibly to be a potential adverse effect of treatment (e.g. with certain taxane compounds or angiogenesis inhibitors) and the measurable tumor has met criteria for response or stable disease, the cytological confirmation of the neoplastic origin of any effusion that appears or worsens during treatment can be considered, in order to differentiate response (or stable disease) and disease progression.

2 ASSESSMENT OF TUMOR RESPONSE

2.1 Assessment of Total Tumor Load and Measurable Lesions

To assess the objective response or future progression, it is necessary to estimate the overall tumor burden at baseline and use this as a comparator for subsequent measurements. Only patients with measurable disease at baseline should be included in protocols where objective tumor response is the primary endpoint. Measurable disease is defined by the presence of at least one measurable lesion. In studies where the primary endpoint is tumor progression (either time to progression or proportion with progression at a fixed date), the protocol must specify if entry is restricted to those with measurable disease or whether patients having non-measurable disease only are also eligible.

2.2 Baseline Documentation of 'Target' and 'Non-target' Lesions

In the presence of more than one measurable lesion at baseline period, a maximum of 5 lesions in total (and a maximum of 2 lesions per organ) representing all involved organs should be identified as target lesions, and should be measured and recorded at baseline (this means that if the patient has only 1 or 2 involved organs, only a maximum of 2 or 4 lesions may be recorded separately).

Target lesions should be selected on the basis of their size (those with the longest diameter) and their suitability (in representing all involved organs) for accurate repeated measurements. If the largest lesion does not lend itself to reproducible measurement, the next largest lesion which can be measured reproducibly should be selected.

Lymph nodes merit special mention since they are normal anatomical structures that may be visible by imaging even if not involved by tumor. Pathological nodes defined as measurable and may be identified as target lesions must meet the criterion of a short axis of ≥ 15 mm by CT scan. Only the short axis of these nodes will contribute to the baseline sum. The short axis of the node is the diameter normally used by radiologists to judge if a node is involved by solid tumor. Nodal size is normally reported as two dimensions in the plane in which the image is obtained (for CT scan this is almost always the axial plane; for MRI the plane of acquisition may be axial, sagittal or coronal), with the short axis as the minimum value. For example, an abdominal node which is reported as being 20 mm × 30 mm has a short axis of 20 mm and can be qualified as a malignant, measurable node. In this example, 20 mm should be recorded as the measurement value of the node. All other pathological nodes (those with short axis ≥ 10 mm but < 15 mm) should be considered non-target lesions. Nodes that have a short axis < 10 mm are considered non-pathological and should not be recorded or followed.

The sum of diameters (longest for non-lymph node target lesions, shortest for lymph node target lesions) of all target lesions will be calculated and reported as the sum of baseline diameters. If lymph nodes are to be included in the sum, then as noted above, only the short axis is included in the sum. The sum of baseline diameters shall serve as a reference for the further characterization of any objective tumor regression in the measurable dimension of the disease.

All other lesions (or sites of disease) including pathological lymph nodes should be identified as non-target lesions and should also be recorded at baseline. Measurements are not required and these lesions should be followed as 'present', 'disappear', or in rare cases 'unequivocal progression'. In addition, it is possible to record multiple non-target lesions involving the same organ as a single item on the case record form (e.g. 'multiple enlarged pelvic lymph nodes' or 'multiple liver metastases').

2.3 Response Evaluation Criteria

2.3.1 Evaluation of target lesions

Complete Response (CR): Disappearance of all target lesions. Any pathological lymph nodes (whether target or non-target) must have reduction in short axis to < 10 mm.

Partial Response (PR): At least a 30% decrease in the sum of diameters of all target lesions, taking as reference the baseline sum diameters.

Progressive Disease (PD): At least a 20% increase in the sum of diameters of target lesions, taking as reference the smallest sum on study (this includes the baseline sum if that is the smallest on study). In addition, the sum must also demonstrate an absolute increase of at least 5 mm (the appearance of one or more new lesions is also considered progression).

Stable Disease (SD): Neither sufficient shrinkage to qualify for PR nor sufficient increase to qualify for PD, taking as reference the smallest sum diameters while on study.

2.3.2 Special notes on the assessment of target lesions

Lymph nodes: Lymph nodes identified as target lesions should always have the actual short axis measurement recorded (measured in the same anatomical plane as the baseline examination), even if the nodes regress to below 10 mm on study. This means that when lymph nodes are included as target lesions, the 'sum' of lesions may not be zero even if complete response criteria are met, since a normal lymph node is defined as having a short axis of < 10 mm. Target lymph nodal lesions should be recorded in a specific section of the case report form or other data collection methods: Each node must achieve a short axis < 10 mm to qualify for CR; For PR, SD, and PD, the actual short axis measurement of the nodes is to be included in the sum of diameters of target lesions.

Target lesions that become 'too small to be measured': While on study, all lesions (nodal and non-nodal) recorded at baseline should have their actual measurements recorded at each subsequent evaluation, even when they are very small (e.g. 2 mm). However, there are occasions when lesions or lymph nodes which are recorded as target lesions at baseline become so faint on CT scan that the radiologist may not feel comfortable assigning an exact measure and may report them as being 'too small to be measured'. In such a case, it is important that a value is recorded on the case report form. If it is the opinion of the radiologist that the lesion has likely disappeared, the measurement may be recorded as 0 mm. If the lesion is believed to be present and is faintly seen, a default value of 5 mm may be recorded (It is less likely that this rule will be used for lymph nodes since normal lymph nodes usually have definite sizes and are frequently surrounded by fat, such as in the retroperitoneum; however, if a lymph node is believed to be present, and is faintly seen but hard to measure, a default value of 5 mm should be recorded as well). The default value of 5 mm is derived from the CT slice thickness (but the default value of 5 mm should not be changed with varying CT slice thickness). The measurement of these lesions (that are too small and not measurable) is potentially non-reproducible, therefore providing this default value will prevent false responses or progressions based upon measurement error. To reiterate, however, if the radiologist is able to provide an actual measure, that should be recorded, even if it is less than 5 mm.

Lesions that split or coalesce on treatment. When non-nodal lesions split into 'fragments', the longest diameters of the fragmented portions should be added together to calculate the sum of (diameters of) target lesions. Similarly, as lesions coalesce, a plane between them may be maintained to help provide the maximal diameter measurements of each lesion. If the lesions have truly coalesced such that they are no longer separable, the vector of the longest diameter in this instance should be the maximal longest diameter for the 'coalesced lesion'.

2.3.3 Evaluation of non-target lesions

This section provides the definitions of the criteria used to determine tumor response for non-target lesions. While some non-target lesions may be measurable, they need not be measured and instead should be assessed only qualitatively at the time points specified in the protocol.

Complete Response (CR): Disappearance of all non-target lesions and normal levels of tumor markers. All lymph nodes must be non-pathological in size (i.e. < 10 mm short axis).

Non-CR/Non-PD: Persistence of one or more non-target lesion(s) and/or maintenance of tumor marker level above the normal limits.

Progressive Disease (PD): Unequivocal progression of existing non-target lesions. Note: the appearance of one or more new lesions is also considered as progression.

2.3.4 Special notes on assessment of progression of non-target lesions

The concept of progression of non-target lesions requires additional explanation as follows:

When the patient has measurable non-target lesions, even when target disease has been assessed as SD or PR, the total tumor burden must have increased sufficiently to merit discontinuation of therapy for the case to be defined as "unequivocal progression". A modest 'increase' in the size of one or more non-target lesions is usually not sufficient to qualify for unequivocal progression status. The designation of overall progression solely on the basis of change in non-target disease in the face of SD or PR of target disease will therefore be extremely rare.

When the patient has only unmeasurable non-target disease: This circumstance arises in some phase III trials when it is not a criterion of study entry to have measurable disease. The same general concepts mentioned above are also applicable here. However, in this instance, there is a lack of measurable disease assessment and worsening in non-target disease cannot be easily quantified (by definition: if all lesions are truly non-measurable), hence a useful test can be applied to assess unequivocal progression if the increase in overall disease burden based on the change in non-measurable disease is comparable in magnitude to the increase that would be required to declare PD for measurable disease. E.g., an increase in tumor burden representing an additional 73% increase in 'volume' (which is equivalent to a 20% increase in diameter in a measurable lesion). An increase in ascites from "slight" to "large", an increase in lymphangitic disease from "localized" to "widespread", or may be described in protocols as "sufficient to require a change in therapy". Examples include an increase in pleural effusion from trace to large, an increase in lymphangitic disease from localized to widespread, or may be described in protocols as "sufficient to require a change in therapy". If unequivocal progression is seen, the patient should be considered to have had overall PD at that point. While it would be ideal to have objective criteria to apply to non-measurable lesions, the very nature of that disease makes it impossible to do so, and therefore the increase must be substantial.

2.3.5 New lesions

The appearance of new malignant lesions denotes disease progression; therefore, some comments on detection of new lesions are important. There are no specific criteria for the identification of new radiographic lesions; however, the finding of a new lesion should be unequivocal: E.g., disease progression should not be attributable to differences in scanning technique, changes in imaging modality, or findings thought to represent something other than tumor (for example, some 'new' bone lesions may be simply healing or relapse of pre-existing lesions). This is particularly important when the patient's baseline lesions show partial or complete response. For example, necrosis of a liver lesion may be reported on a CT scan report as a 'new' cystic lesion, which it is not.

A lesion identified on a follow-up study in an anatomical location that was not scanned at baseline is considered a new lesion and will indicate disease progression. An example of this is the patient who has visceral disease at baseline and while on study has a CT or MRI brain ordered which reveals metastases. The patient's brain metastases are considered to be evidence of PD even if he/she did not have brain imaging at baseline.

If a new lesion is equivocal, for example, because of its small size, continued therapy and follow-up evaluation will clarify if it represents truly a new disease. If repeat scans confirm there is definitely a new lesion, then progression should be declared using the date of the initial scan.

While FDG-PET response assessments require additional study, it is sometimes reasonable to incorporate the use of FDG-PET scanning to complement CT scanning in assessment of progression (particularly in possible 'new' diseases). New lesions on the basis of FDG-PET imaging can be identified according to the following algorithm:

a. Negative FDG-PET at baseline, with a positive FDG-PET at follow-up indicates new lesion and should be assessed as PD.

b. No FDG-PET at baseline and a positive FDG-PET at follow-up: If the positive FDG-PET at follow-up corresponds to a new site of lesion confirmed by CT, this is PD. If the positive FDG-PET at follow-up is not confirmed as a new site of lesion on CT, additional follow-up CT scans are needed to determine if there is truly a progression occurring at that site (if so, the date of PD will be the date of the initial abnormal FDG-PET scan). If the positive FDG-PET at follow-up corresponds to an existing site of lesion on CT that is not progressing on the basis of the anatomic images, this is not PD.

2.4 Evaluation of Best Overall Response

The best overall response is the best response recorded from the start of the study treatment until the end of treatment, taking into account any requirement for confirmation. When a response may not be documented until after the end of therapy, protocols should clarify if post-treatment assessments are to be considered in the determination of best overall response. Protocols must specify how any new therapy introduced before progression will affect the determination of best response. Determination of best overall response will depend on the findings of both target and non-target lesions, as well as the appearance of new lesions. Furthermore, depending on the nature of the study and the protocol requirements, confirmatory measurement may also be required. Specifically, in non-randomized trials where tumor response is the primary endpoint, confirmation of PR or CR is needed to determine which is the 'best overall response'.

2.4.1 Time point response

It is assumed that response assessment occurs at each time point specified in the protocol. Table 1 on the following page summarizes the calculation of overall response status at each time point for patients who have measurable disease at baseline. Refer to Table 2 when patients have non-measurable (i.e. non-target) lesions only.

Table 1. Time point response: Patients with target (+/– non-target) lesion

| **Target lesions** | **Non-target lesions** | **New lesions** | **Overall response** |
| --- | --- | --- | --- |
| CR | CR | None | CR |
| CR | Non-CR/Non-PD | None | PR |
| CR | Not evaluated | None | PR |
| PR | Non-PD or not all evaluated | None | PR |
| SD | Non-PD or not all evaluated | None | SD |
| Not all evaluated | Non-PD | None | NE |
| PD  Any  Any | Any  PD  Any | Yes or No  Yes or No  Yes | PD  PD  PD |
| Note: CR = complete response, PR = partial response, SD = stable disease, PD = PD, NE = not evaluable. | | | |

Table 2. Time point response: Patients with non-target lesions only

| **Non-target lesions** | **New lesions** | **Overall response** |
| --- | --- | --- |
| CR | None | CR |
| Non-CR/Non-PD | None | Non-CR or Non-PD^a^ |
| Not all evaluated | None | Not evaluated |
| Unequivocal PD | Yes or No | PD |
| Any | Yes | PD |

a: 'Non-CR/non-PD' is preferred over 'SD' for non-target lesion. Since SD is increasingly used as endpoint for assessment of efficacy in some trials, so to assign this category when no lesions can be measured is not advised.

2.4.2 Missing assessments and inevaluable designation

When imaging/measurement is not conducted at a particular time point, then patients are deemed "not evaluable" (NE) at such time point. When partial lesion measurement is carried out during one assessment, such assessment is deemed "not evaluable" (NE) at that time point, unless a convincing argument can be made that the contribution of the individual missing lesion(s) would not change the assigned time point response. This is very likely to happen in the case of PD. For example, if a patient had a baseline sum of 50 mm with three measured lesions and at follow-up only two lesions were assessed, but those reached a sum of 80 mm, the patient will have achieved PD status, regardless of the contribution of the missing lesion.

2.4.3 Best overall response: All time points

The best overall response is determined once all the data for the patient is known.

Best response determination in trials where confirmation of CR or PR is not required: Best response in these trials is defined as the best response across all time points (for example, a patient who has SD at the first assessment, PR at the second assessment, and PD on last assessment has a best overall response of PR). When SD is deemed to be best response, the assessment time must also meet the minimum time from baseline as specified in the protocol. If SD is the best response but assessment time fails to reach the specified minimum duration, the patient's best response will depend on subsequent assessments. For example, a patient who has SD at the first assessment, PD at the second and does not meet the minimum duration for SD, will be assigned a best response of PD. If the same patient is lost to follow-up after the first SD assessment, the patient would be deemed NE.

Best response determination in trials where confirmation of complete or partial response is required: Complete response or partial response may be claimed only if the criteria for each are met at a subsequent time point as specified in the protocol (generally 4 weeks later). In such a circumstance, the best overall response can be interpreted as in Table 3.

Table 3. Best overall response when confirmation of CR and PR required

| **Overall response First time point** | **Overall response Subsequent time point** | **Best overall response** |
| --- | --- | --- |
| CR | CR | CR |
| CR | PR | SD, PD or PR^a^ |
| CR | SD | SD provided minimum criteria for SD duration met, otherwise, PD |
| CR | PD | SD provided minimum criteria for SD duration met, otherwise, PD |
| CR | NE | SD provided minimum criteria for SD duration met, otherwise, NE |
| PR | CR | PR |
| PR | PR | PR |
| PR | SD | SD |
| PR | PD | SD provided minimum criteria for SD duration met, otherwise, PD |
| PR | NE | SD provided minimum criteria for SD duration met, otherwise, NE |
| NE | NE | NE |

Note: CR = complete response, PR = partial response, SD = stable disease, PD = PD, NE = not evaluable.

a: If a CR is truly met at the first time point, then any disease seen at a subsequent time point, even disease meeting PR criteria relative to baseline, makes the disease PD after that point (since disease must have reappeared after CR). Best response would depend on whether the minimum duration for SD was met. However, sometimes CR may be claimed when subsequent scans suggest small lesions were likely still present and in fact the patient had PR, not CR at the first time point. Under these circumstances, the initial CR should be changed to PR and the best response is PR.

2.4.4 Special notes on response assessment

When nodal disease is included in the sum of target lesions and the nodes decrease to 'normal' size
(< 10 mm), they may still have a measurement reported on scans. This measurement should be recorded even though the nodes are normal in order not to overstate progression should it be based on the increase in size of the nodes. As noted earlier, this means that patients with CR may not have a total sum of zero on the case report form (CRF).

In trials where confirmation of response is required, repeated "NE" time point assessments may complicate best response determination. The analysis plan for the trial must specify how missing data/assessments will be addressed in determination of response and progression. For example, in most trials, it is reasonable to consider a patient with time point responses of PR-NE-PR as a confirmed response.

Patients with a global deterioration of health status requiring discontinuation of treatment without objective evidence of disease progression at that time should be reported as symptomatic deterioration. Every effort should be made to evaluate objective progression even after discontinuation of treatment. Symptomatic deterioration is not a descriptor of an objective response: it is a reason for stopping study therapy. The response status of such patients is to be determined by assessment of target and non-target lesions as shown in Attached Tables 1-3.

Conditions that be defined as 'early progression, early death, and NE' are study specific and should be clearly described in each protocol (depending on treatment duration and treatment periodicity).

In some circumstances, it may be difficult to distinguish residual disease from normal tissues. When the evaluation of complete response depends upon such a definition, it is recommended that the residual lesion be investigated (fine needle aspirate/biopsy) before assigning a status of complete response. FDG-PET may be used to upgrade a response to a CR in a manner similar to a biopsy in cases where a residual radiographic abnormality is thought to represent fibrosis or scarring. The use of FDG-PET in this circumstance should be prospectively described in the protocol and supported by disease-specific medical literature for the indication. However, it must be acknowledged that both approaches may lead to false positive CR due to limitations of FDG-PET and biopsy (including their resolution/sensitivity).

For equivocal findings of progression (e.g. very small and uncertain new lesions; cystic changes or necrosis in existing lesions), treatment may continue until the next scheduled assessment. If at the next scheduled assessment, progression is confirmed, the date of progression should be the earlier date when progression was suspected.

2.5 Frequency of Tumor Re-evaluation

Frequency of tumor re-evaluation while on treatment should be based on the study plan, type of tumor and schedule of treatment. However, in Phase II clinical trials where the efficacy of therapy is not known, follow-up every 6–8 weeks (timed to coincide with the end of a cycle) is reasonable. A shorter or longer evaluation cycle could be determined according to specific regimens or circumstances. Protocols should specify which organ sites are to be evaluated at baseline (usually those most likely to be involved with metastatic disease for the tumor type under study) and the frequency of re-evaluations. Normally, all target and non-target sites are evaluated at each assessment. In selected circumstances, certain non-target organs may be evaluated less frequently. For example, bone scans may need to be performed only when complete response is identified in target lesion or when progression in bone is suspected.

After the end of the treatment, the need for repetitive tumor evaluations depends on whether the trial has response rate or the time to an event (progression/death) is considered as the study endpoint. If 'time to an event' (e.g., time to progression, disease-free survival, progression-free survival) is the primary endpoint of the study, then routine scheduled re-evaluation of protocol-specified sites of disease is warranted. In randomized controlled trials in particular, scheduled assessments should be performed according to calendar schedule (e.g., every 6–8 weeks during treatment or every 3–4 months post-treatment) and should not be affected by delays in therapy, drug holidays or any other events that might lead to imbalance in a treatment arm in the timing of disease assessment.

2.6 Confirmation of Measurement/Duration of Response

2.6.1 Confirmation

In non-randomized trials where response is the primary endpoint, the confirmation of PR and CR is required to ensure responses identified are not the result of measurement error. This will also allow appropriate interpretation of results in the context of historical data where confirmation of response has been traditionally required. However, in all other circumstances, e.g. in randomized trials (Phase II or III) or studies where stable disease or progression are the primary endpoints, confirmation of response is not required since it will not add value to the interpretation of trial results. However, elimination of the requirement for response confirmation may increase the importance of a central review to protect against bias, particularly in studies that are not blinded.

When assessed as SD, measurements must have met the SD criteria defined in the study protocol at least once after study entry at a minimum interval (in general not less than 6–8 weeks).

2.6.2 Duration of overall response

The duration of overall response is measured from the time evaluation criteria are first met for CR/PR (whichever is first recorded) until the first date that recurrent or PD is objectively recorded (taking as reference for PD the smallest measurements recorded on study). The duration of overall complete response is measured from the time evaluation criteria are met for CR until the first date that recurrent disease is objectively recorded.

2.6.3 Duration of stable disease

Stable disease is measured from the start of the treatment (in randomized trials, from the date of randomization) until the progression, taking as reference the smallest sum on study (if the baseline sum is the smallest, this is the reference for calculation of PD). The clinical relevance of the duration of stable disease varies in different studies and diseases. If the proportion of patients achieving stable disease for a minimum period of time is an endpoint in a particular trial, the protocol should specify the minimum time interval required between two measurements for determination of stable disease.

Note: The duration of response and stable disease as well as the progression-free survival are influenced by the frequency of follow-up after baseline evaluation. It is not in the scope of this guideline to define a standard follow-up frequency. The frequency should take into account many parameters including disease types and stages, treatment periodicity and standard practice. However, these limitations of the precision of the measured endpoint should be taken into account if comparisons between trials are to be made.

2.7 PFS/TTP

2.7.1 Phase II clinical trials

This guideline focuses primarily on the use of objective response as the endpoint in Phase II clinical trials. In some circumstances, response rate may not be the optimal method to assess the potential anticancer activity of new agents/regimens. In such cases, 'progression-free survival' (PFS) or the 'proportion progression-free' (PPF) at landmark time points might be considered appropriate alternatives to provide an initial signal of biologic effect of new agents. It is clear, however, that in an uncontrolled trial, these measures are subject to criticism since an apparently promising observation may be related to biological factors such as patient selection and not the impact of drug intervention. Thus, Phase II clinical trials utilizing these endpoints are best designed with a randomized control. Exceptions may exist where the behavior patterns of certain cancers are so consistent (and usually consistently poor), that a non-randomized trial is justifiable. However, in these cases, it will be essential to document with care the basis for estimating the expected PFS or PPF in the absence of positive control.

2.7.2 Phase III clinical trials

Phase III trials in advanced cancers are increasingly designed to evaluate progression-free survival or time to progression as the primary outcome of interest. Assessment of PD is relatively straightforward if the protocol requires all patients to have measurable lesion. However, standards are generally adopted to restrict the enrollment of some subjects: This may lead to a lack of general application of the test results as well as difficulties in subject recruitment. Therefore, an increasing number of trials allow entry of patients only with measurable lesion, or only those with non-measurable lesion. When doing so, care must be taken to explicitly describe the findings which would qualify for PD for those patients without measurable lesions. Furthermore, under such circumstances, the protocol must indicate that the number of target lesions may be relaxed from five to three. Furthermore, if available, effective tumor markers may be used to measure PD (as has been proposed for ovarian cancer) to help determine the PD. Centralized blinded review of imaging studies or source imaging reports may be required to verify 'unequivocal progression' if important drug development or drug approval decisions are to be based on the study outcome. Finally, as noted earlier, because the date of PD is subject to evaluation bias, so the timing of evaluation in each treatment arm should be the same.

2.8 Independent Review of Response and Progression

For clinical trials where objective response (CR + PR) is the primary endpoint, and in particular where key drug development decisions are based on the observation of a minimum number of responders, it is recommended to have independent experts review all responses. If the study is a randomized trial, it would be ideal for reviewers to be blinded. The best approach would be to conduct a simultaneous review of the patients' files and radiological images.

Independent review of PD presents some additional complex issues: For example, there are statistical problems with the use of central-review-based progression time in place of investigator-based progression time due to the potential introduction of informative censoring when the former precedes the latter.

2.9 Reporting Best Response Results

2.9.1 Phase II clinical trials

When response is the primary endpoint, all enrolled patients must have measurable lesions.

All enrolled subjects must be accounted for in the results report, even in the event of major protocol deviations or if the subjects whose efficacy is deemed not evaluable. Each patient will be assigned to one of the following:

1. Complete response

2. Partial response

3. Stable disease

4. Progressive disease

5. Not evaluable: Specify reasons (e.g., early death due to tumor/toxicity; tumor assessments not repeated/incomplete; others).

Under normal circumstances, all eligible patients should be included in the denominator for the calculation of the response rate in Phase II clinical trials (some protocols will require the inclusion of all treated patients).

Response rates are usually assessed using a two-sided 95% CI. Trial conclusions should be based on the response rate for all eligible or all treated patients, instead of being based on a selected evaluable subgroup.

2.9.2 Phase III clinical trials

In Phase III clinical trials, response rate may be an evaluation indicator of the anti-tumor activity and is almost always a secondary endpoint. Observed differences in response rate may not predict the clinical benefits for the subject population. If objective response rate is selected as a primary endpoint for a Phase III clinical trial (only in circumstances where a direct relationship between objective tumor response rate and a clinically relevant therapeutic benefit can be unambiguously demonstrated for the subject population), the same criteria as those applying to Phase II trials may be used and all enrolled patients should have at least one measurable lesion.

In cases where response rate is a secondary endpoint and not all enrolled patients have measurable lesion, the protocol must specify the method for reporting overall best response rates in advance. In practice, response rate may be reported using either an "intention-to-treat" analysis (including all patients) or an analysis where only the subgroup of patients with measurable lesion at baseline are included.

The protocol should specify how response results will be reported, including any subgroup analyses that are planned. The original version of RECIST suggested that a "relaxed" interpretation of the RECIST guidelines (for example, reducing the number of lesions measured) may be adopted in Phase III clinical trials. However, this is no longer recommended in the revised guidelines. The revised guidelines stated that RECIST should be applied to all clinical trials, and these clinical trials should use the anatomical evaluation of tumor response or PD as the endpoint.

## Appendix 5 Comparison between iRECIST and RECIST 1.1

The following contents are internally translated information that has been obtained from the iRECIST website. Please refer to the original version in English at <https://recist.eortc.org/irecist/>.

The RECIST Working Group developed a guideline for the use of modified RECIST in immunotherapy (iRECIST). These guidelines are not intended to define or guide clinical practice or treatment decisions, but rather to provide a consistent framework for the management of data collected in clinical trials of immune-based therapies.

|  | **RECIST 1.1** | **iRECIST** |
| --- | --- | --- |
| Definition of measurable and non-measurable lesions; number and site of target lesions | Diameter of measurable lesions ≥ 10 mm (nodal lesions ≥ 15 mm); maximum of 5 lesions (2 per organ); all other lesions are considered non-target (short-axis must be ≥ 10 mm for nodal lesions) | Same as RECIST 1.1; new lesions are evaluated according to RECIST 1.1 and recorded separately in the case report form (but not included in the sum of target lesions identified at baseline) |
| CR, PR, or SD | No PD prior to CR, PR, or SD | iUPD (one or more instances), but not iCPD, may appear before iCR, iPR, or iSD |
| Confirmation of CR or PR | Only required for non-randomized trials | As per RECIST 1.1 |
| Confirmation of SD | Not required | As per RECIST 1.1 |
| New lesions | Deemed as PD; recorded but not measured | Deemed as iUPD, but only when there are other new lesions or increase in size of new lesions at the next assessment (sum of diameters of new target lesions ≥ 5 mm or any increase of new non-target lesions), will iCPD be confirmed according to this category; new lesions, where none have previously been recorded can also confirm iCPD |
| Independent blinded review and central collection of scans | Recommended in certain circumstances — for example, in trials planned for marketing approval (determining endpoints based on progress) | Collection of scans (but not independent review) recommended for all trials |
| Confirmation of PD | Not required (unless equivocal) | Required |
| Consideration of clinical status | Not included in assessment | Consider clinical stability when deciding whether to continue treatment after iUPD |


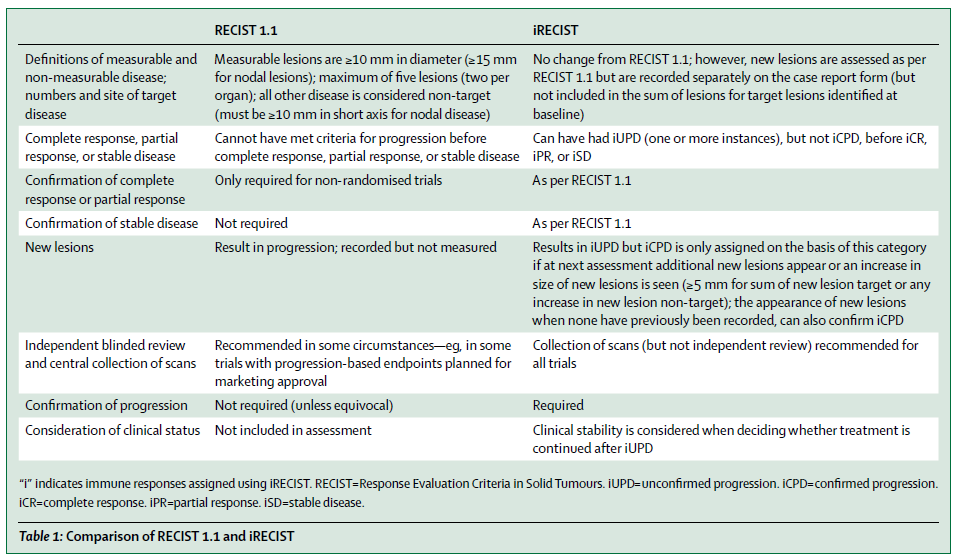


|  | **RECIST 1.1** | **iRECIST** |
| --- | --- | --- |
| Definitions of measurable/non-measurable lesions | Same | Same |
| Definitions of target and non-target lesions | Same | Same |
| Measurement and management of nodal lesions | Same | Same |
| Sum of measures of lesions | Same | Same |
| Definitions and duration of CR/PR/SD | Same | Same |
| Confirmation of CR and PR when appropriate | Same | Same |
| Definitions of progression of target and non-target lesions (iUPD in iRECIST) | Same | Same |
| Management of new lesions | None | New |
| Time point response after determination of PD as per RECIST 1.1 | None | New |
| Confirmation of PD | None | New |
| Document reasons for unconfirmed PD | None | New |
| Consideration and documentation of clinical status | None | New |

The main differences between iRECIST and RECIST 1.1 are in the evaluation of PD.

The evaluations of iCR (immune complete response), iPR (immune partial response), and iSD (immune stable disease) in iRECIST are identical to the evaluations of CR (complete response), PR (partial response), and SD (stable disease) in RECIST vl.1.

There are changes in the determination of iPD (immune PD). The first PD determination based on RECIST 1.1 should be iUPD (immune unconfirmed PD) in iRECIST. Following that, an efficacy confirmation (at least 4 weeks, not more than 8 weeks) is carried out. If the PD is found during confirmatory evaluation, then response is determined to be iCPD (immune confirmed PD). If the criteria for CR, PR, and SD in RECIST 1.1 are met during confirmatory evaluation, then response is determined to be iCR, iPR, and iSD. If there is no change in tumor status compared with iUPD determination during confirmatory evaluation, then response is determined to be iUPD.

**New lesions**

iRECIST is an updated evaluation of new lesions. If there is more than 1 measurable lesion during assessment, all lesions should be recorded and measured. If the total number of lesions does not exceed 5 (not more than 2 per organ), the lesions will be considered as new lesions-target (NLT). However, NLTs should not be included in the baseline target lesion SOM. New lesions other than NLTs are considered as new lesions-non-target (NLNT).

**iRECIST Time Point Response (English)**

| **Target lesions*** | **Non-target lesions*** | **New lesions*** | **Time point response** | |
| --- | --- | --- | --- | --- |
|  |  |  | **No prior iUPD**** | **Prior iUPD**; ***** |
| iCR | iCR | No | iCR | iCR |
| iCR | Non-iCR/Non-iUPD | No | iPR | iPR |
| iPR | Non-iCR/Non-iUPD | No | iPR | iPR |
| iSD | Non-iCR/Non-iUPD | No | iSD | iSD |
| iUPD with no change OR decrease from last TP | iUPD with no change OR decrease from last TP | Yes | NA | NLs confirms iCPD if NLs were previously identified and increase in size (≥ 5 mm in SOM for NLT or ≥ 5 mm in SNL for NLNT) or number.  If no change in NLs (size or number) from last TP, remains iUPD |
| iSD | iUPD | No | iUPD | Remains iUPD unless iCPD confirmed based in further increase in size of NT disease (need not meet RECIST 1.1 criteria for unequivocal PD) |
| iUPD | Non-iCR/Non-iUPD | No | iUPD | Remains iUPD unless iCPD confirmed based on: further increase in SOM of ≥ 5 mm, otherwise remains iUPD |
| iUPD | iUPD | No | iUPD | Remains iUPD, unless iCPD confirmed based on further increase in:   - previously identified T lesion iUPD ≥ 5 mm and/or - NT lesion iUPD (prior assessment - need not be unequivocal PD) |
| iUPD | iUPD | Yes | iUPD | Remains iUPD, unless iCPD confirmed based on further increase in:   - previously identified T lesion iUPD ≥ 5 mm and/or - previously identified NT lesion iUPD (need not be unequivocal) and/or - size or number of new lesions previously identified |
| Non-iUPD/PD | Non-iUPD/PD | Yes | iUPD | Remains iUPD unless iCPD confirmed based on increase in size or number of new lesions previously identified |

Note:

*Using RECIST 1.1 principles. If no PSPD occurs, RECIST 1.1 and iRECIST categories for CR, PR, and SD would be the same.

**in any lesion category.

***previously identified in assessment immediately prior to this TP.

**iRECIST Time Point Response**

| **Target lesions*** | **Non-target lesions*** | **New lesions*** | **Time point response, TPR** | |
| --- | --- | --- | --- | --- |
|  |  |  | **No prior iUPD**** | **Prior iUPD**; ***** |
| iCR | iCR | No | iCR | iCR |
| iCR | Non-iCR/Non-iUPD | No | iPR | iPR |
| iPR | Non-iCR/Non-iUPD | No | iPR | iPR |
| iSD | Non-iCR/Non-iUPD | No | iSD | iSD |
| iUPD with no change OR  decrease from last  TP | iUPD with no change OR decrease from last TP | Yes | NA | NLs confirms iCPD if NLs were previously identified and increase in size (≥ 5 mm in SOM for NLT or any increase for NLNT) or number. If no change in NLs (size or number) from last TP, remains iUPD |
| iSD | iUPD | No | iUPD | Remains iUPD unless iCPD confirmed based in further increase in size of NT disease (need not meet RECIST 1.1 criteria for unequivocal PD) |
| iUPD | Non-iCR/Non-iUPD | No | iUPD | Remains iUPD unless iCPD confirmed based on:   - further increase in SOM of at least 5 mm, otherwise remains iUPD |
| iUPD | iUPD | No | iUPD | Remains iUPD unless iCPD confirmed based on further increase in:   - previously identified T lesion iUPD SOM ≥ 5 mm and/or - NT lesion iUPD (prior assessment - need not be unequivocal PD) |
| iUPD | iUPD | Yes | iUPD | Remains iUPD unless iCPD confirmed based on further increase in:   - previously identified T lesion iUPD ≥ 5 mm and/or - previously identified NT lesion iUPD (need not be unequivocal) and/or - size or number of new lesions previously identified |
| Non-iUPD/PD | Non-iUPD/PD | Yes | iUPD | Remains iUPD unless iCPD confirmed based on   - increase in size or number of new lesions previously identified |
| * Using RECIST 1.1 principles. If no PSPD occurs, RECIST 1.1 and iRECIST categories for CR, PR and SD would  be the same. ** in any lesion category. *** previously identified in assessment immediately prior to this TP. | | | | |

**iRECIST Best Overall Response (iBOR)**


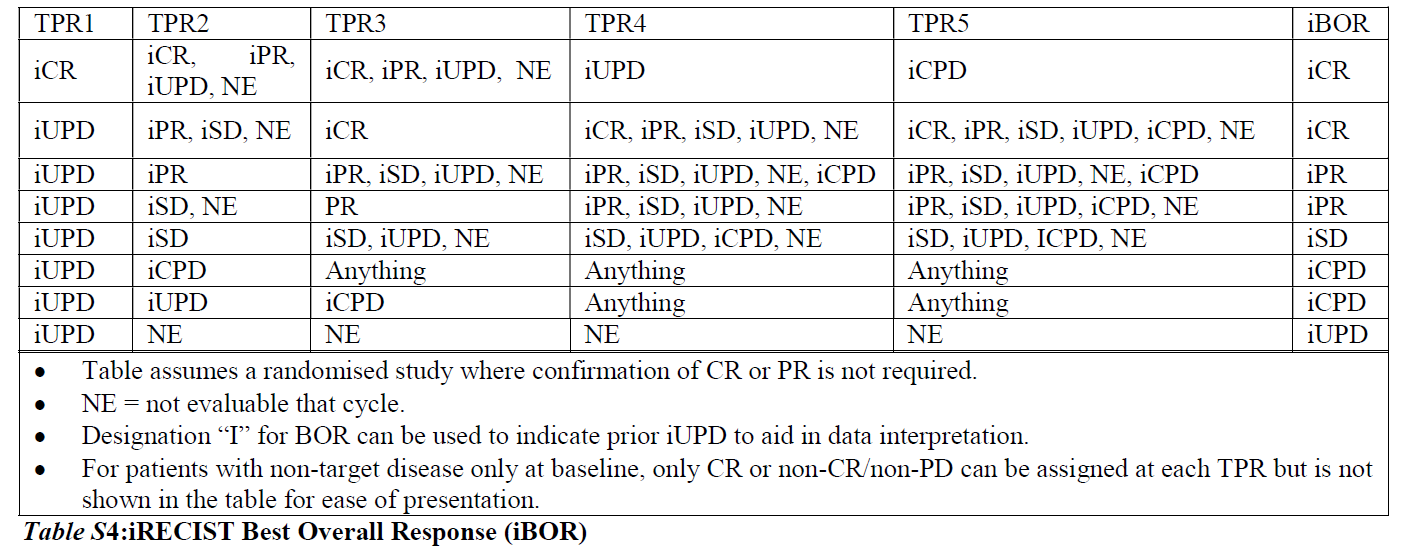


Note:

iCR: immune complete response; iPR: immune partial response; iSD: immune stable disease; iUPD: immune unconfirmed PD; iCPD: immune confirmed PD; NL: new lesion; NLT: new lesion target; NLNT: new lesion nontarget; T: target; TP: time point; NA: not applicable; NE: not evaluable
